# Bayesian Inference Associates Rare *KDR* Variants With Specific Phenotypes in Pulmonary Arterial Hypertension

**DOI:** 10.1161/CIRCGEN.120.003155

**Published:** 2020-12-15

**Authors:** Emilia M. Swietlik, Daniel Greene, Na Zhu, Karyn Megy, Marcella Cogliano, Smitha Rajaram, Divya Pandya, Tobias Tilly, Katie A. Lutz, Carrie C.L. Welch, Michael W. Pauciulo, Laura Southgate, Jennifer M. Martin, Carmen M. Treacy, Christopher J. Penkett, Jonathan C. Stephens, Harm J. Bogaard, Colin Church, Gerry Coghlan, Anna W. Coleman, Robin Condliffe, Christina A. Eichstaedt, Mélanie Eyries, Henning Gall, Stefano Ghio, Barbara Girerd, Ekkehard Grünig, Simon Holden, Luke Howard, Marc Humbert, David G. Kiely, Gabor Kovacs, Jim Lordan, Rajiv D. Machado, Robert V. MacKenzie Ross, Colm McCabe, Shahin Moledina, David Montani, Horst Olschewski, Joanna Pepke-Zaba, Laura Price, Christopher J. Rhodes, Werner Seeger, Florent Soubrier, Jay Suntharalingam, Mark R. Toshner, Anton Vonk Noordegraaf, John Wharton, James M. Wild, Stephen John Wort, NIHR BioResource for Translational Research - Rare Diseases, National Cohort Study of Idiopathic and Heritable PAH, PAH Biobank Enrolling Centers’ Investigators, Allan Lawrie, Martin R. Wilkins, Richard C. Trembath, Yufeng Shen, Wendy K. Chung, Andrew J. Swift, William C. Nichols, Nicholas W. Morrell, Stefan Gräf

**Affiliations:** 1 Department of Medicine (E.M.S., D.P., T.T., C.M.T., M.R.T., N.W.M., S. Gräf), University of Cambridge.; 2 Department of Haematology (D.G., K.M., C.J.P., J.C.S., S. Gräf), University of Cambridge.; 3 NIHR BioResource for Translational Research, Cambridge, United Kingdom (D.G., K.M., J.M.M., C.J.P., J.C.S., N.W.M., S. Gräf).; 4 Department of Pediatrics (N.Z., C.C.L.W.), Columbia University, NY.; 5 Department of Systems Biology (N.Z., Y.S.), Columbia University, NY.; 6 Department of Biomedical Informatics (Y.S.), Columbia University, NY.; 7 Department of Infection, Immunity & Cardiovascular Disease, University of Sheffield (M.C., J.M.W., A.L., A.J.S.).; 8 Sheffield Teaching Hospitals NHS Foundation Trust, United Kingdom (S.R.).; 9 Division of Human Genetics, Cincinnati Children’s Hospital Medical Center (K.A.L., M.W.P., A.W.C., W.C.N.).; 10 Department of Pediatrics, University of Cincinnati College of Medicine, OH (M.W.P., W.C.N.).; 11 Molecular & Clinical Sciences Research Institute, St George’s, University of London, United Kingdom (L.S., R.D.M.).; 12 Department of Clinical Genetics, Amsterdam UMC, Vrije Universiteit Amsterdam, the Netherlands (H.J.B., A.V.N.).; 13 Golden Jubilee National Hospital, Glasgow (C.C.).; 14 Royal Free Hospital, London (G.C.).; 15 Sheffield Pulmonary Vascular Disease Unit, Royal Hallamshire Hospital, United Kingdom (R.C., D.G.K.).; 16 Laboratory for Molecular Genetic Diagnostics, Institute of Human Genetics, Heidelberg University (C.A.E.).; 17 Center for Pulmonary Hypertension, Thoraxklinik gGmbH Heidelberg at Heidelberg University Hospital (C.A.E., E.G.).; 18 Translational Lung Research Center Heidelberg (TLRC), German Center for Lung Research (DZL), Heidelberg, Germany (C.A.E., E.G.).; 19 Département de génétique, hôpital Pitié-Salpêtrière, Assistance Publique-Hôpitaux de Paris & UMR_S 1166-ICAN, INSERM, UPMC Sorbonne Universités, Paris, France (M.E., F.S.).; 20 University of Giessen & Marburg Lung Center (UGMLC), member of the German Center for Lung Research (DZL) and of the Excellence Cluster Cardio-Pulmonary Institute (CPI), Giessen, Germany (H.G., W.S.).; 21 Fondazione IRCCS Policlinico San Matteo, Pavia, Italy (S. Ghio).; 22 Université Paris-Sud, Faculté de Médecine, Université Paris-Saclay (B.G., M.H., D.M.).; 23 AP-HP, Service de Pneumologie, Centre de référence de l’hypertension pulmonaire (B.G., M.H., D.M.).; 24 INSERM UMR_S 999, Hôpital Bicêtre, Le Kremlin-Bicêtre, Paris, France (B.G., M.H., D.M.).; 25 Addenbrooke’s Hospital NHS Foundation Trust, Cambridge (S.H., N.W.M.).; 26 National Heart & Lung Institute, Imperial College London, United Kingdom (L.H., C.M., L.P., C.J.R., J.W., S.J.W., M.R.W.).; 27 Ludwig Boltzmann Institute for Lung Vascular Research (G.K., H.O.).; 28 Medical University of Graz, Austria (G.K., H.O.).; 29 Freeman Hospital, Newcastle upon Tyne (J.L.).; 30 Royal United Hospitals Bath NHS Foundation Trust, Bath (R.V.M.R., J.S.).; 31 Royal Brompton & Harefield NHS Foundation Trust (C.M., L.P., S.J.W.).; 32 Great Ormond Street Hospital, London (S.M.).; 33 Royal Papworth Hospital NHS Foundation Trust (J.P.-Z., M.R.T., N.W.M.).; 34 Department of Medical & Molecular Genetics, King’s College London, United Kingdom (R.C.T.).; 35 Columbia University Irving Medical Center, NY (W.K.C.).

**Keywords:** computed tomography, family history, genetic association studies, pulmonary hypertension, vascular endothelial growth factor receptor

## Abstract

**Background:**

Approximately 25% of patients with pulmonary arterial hypertension (PAH) have been found to harbor rare mutations in disease-causing genes. To identify missing heritability in PAH, we integrated deep phenotyping with whole-genome sequencing data using Bayesian statistics.

**Methods:**

We analyzed 13 037 participants enrolled in the NBR study (NIHR BioResource—Rare Diseases), of which 1148 were recruited to the PAH domain. To test for genetic associations between genes and selected phenotypes of pulmonary hypertension, we used the Bayesian rare variant association method BeviMed.

**Results:**

Heterozygous, high impact, likely loss-of-function variants in the kinase insert domain receptor (*KDR*) gene were strongly associated with significantly reduced transfer coefficient for carbon monoxide (posterior probability=0.989) and older age at diagnosis (posterior probability=0.912). We also provide evidence for familial segregation of a rare nonsense *KDR* variant with these phenotypes. On computed tomographic imaging of the lungs, a range of parenchymal abnormalities were observed in the 5 patients harboring these predicted deleterious variants in *KDR*. Four additional PAH cases with rare likely loss-of-function variants in *KDR* were independently identified in the US PAH Biobank cohort with similar phenotypic characteristics.

**Conclusions:**

The Bayesian inference approach allowed us to independently validate *KDR*, which encodes for the VEGFR2 (vascular endothelial growth factor receptor 2), as a novel PAH candidate gene. Furthermore, this approach specifically associated high impact likely loss-of-function variants in the genetically constrained gene with distinct phenotypes. These findings provide evidence for *KDR* being a clinically actionable PAH gene and further support the central role of the vascular endothelium in the pathobiology of PAH.

## Abstract 2

Supplemental Digital Content is available in the text.

Pulmonary arterial hypertension (PAH) is characterized by pulmonary vascular constriction and obliteration, causing elevation of pulmonary vascular resistance and ultimately, right ventricular failure. Molecular mechanisms, such as aberrant angiogenesis,^1^ metabolic reprogramming, and resistance to apoptosis,^2^ have been proposed to explain pulmonary vessel remodeling. A breakthrough in our understanding of the pathobiology underlying PAH was the discovery of heterozygous germline mutations in the gene encoding the bone morphogenetic protein receptor type 2 (*BMPR2*),^3^ responsible for over 70% of familial PAH cases and 15% to 20% of idiopathic PAH (IPAH) cases. A smaller proportion (up to 10%) of PAH cases are caused by mutations in activin-like kinase 1 (*ACVRL1*),^4^ endoglin (*ENG*),^5^ SMAD family member 9 (*SMAD9*),^6^ caveolin-1 (*CAV1*), involved in colocalization of BMP receptors,^7^ and the potassium channel *KCNK3*, responsible for membrane potential and vascular tone.^8^ We recently identified rare pathogenic variants in growth differentiation factor 2 (*GDF2*), which encodes BMP9 (bone morphogenetic protein 9), a major ligand of the BMPR2/ALK1 (activin receptor-like kinase 1) receptor complex, as well as rare variants in ATPase 13A3 (*ATP13A3*), aquaporin 1 (*AQP1*), and SRY-box 17 (*SOX17*) and reported a list of additional putative genes potentially contributing to the pathobiology of PAH.^9^ Together, the established genes explain ≈25% of cases with IPAH, allowing their reclassification as heritable PAH cases. To identify additional genes harboring potentially causal rare variants in IPAH cases, we increased the cohort size^10^ and deployed a recently developed Bayesian methodology^11^ that incorporates phenotypic data to increase the power to detect rare risk variants.

## Methods

**Figure 1.**
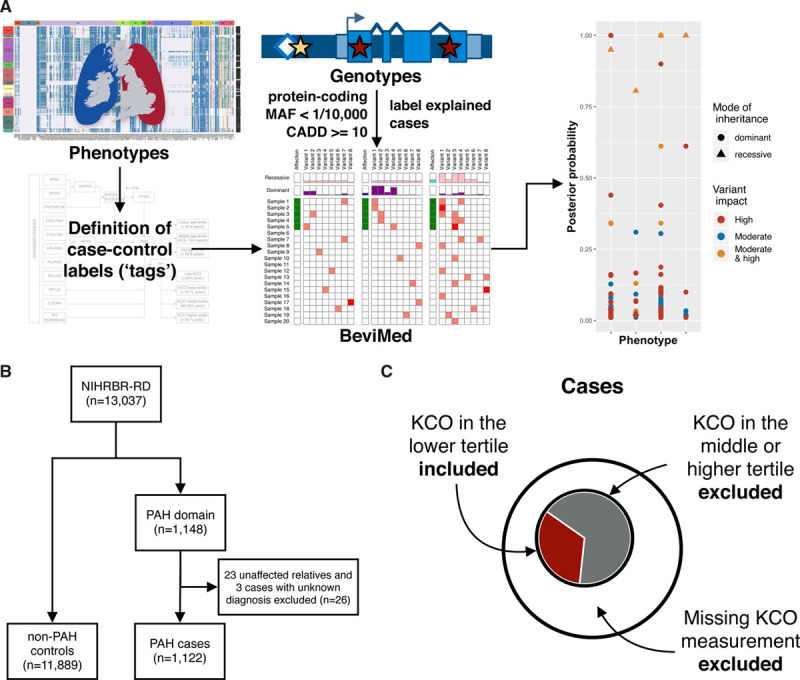
**Design of the genetic association study.** **A**, Overview of the analytical approach. Using deep phenotyping, data tags were assigned to patients who shared phenotypic features. Rare sequence variants, called from whole-genome sequencing data, were filtered, and explained cases were labeled. BeviMed was applied to a set of unrelated individuals to estimate the posterior probability of gene-tag associations. **B**, Consort diagram summarizing the size of the study cohort. **C**, Schematic representation of the definition of cases, exemplified by the transfer coefficient for carbon monoxide (KCO) lower tertile tag. Cases were defined as individuals carrying a particular tag, whereas patients with missing information or those without a tag were removed from the gene-tag association testing. Individuals from non-pulmonary arterial hypertension (PAH) domains served as controls. CADD indicates combined annotation dependent depletion; and MAF, minor allele frequency.

Figure 1A provides an overview of the analysis strategy. The method details are described in the Data Supplement. The data of the NBR study (National Institute for Health Research BioResource—Rare Diseases) have been deposited in the European Genome-Phenome Archive.^10^ The data from the US PAH Biobank and the Columbia University Irving Medical Center are available via an application.^12^

Patients recruited to the NBR study provided informed consent for genetic analysis and clinical data capture (REC REF: 13/EE/0325); patients recruited by European collaborators consented to genetic testing and clinical data collection locally. Institutional review boards at Cincinnati Children’s Hospital Medical Center and Columbia University Irving Medical Center, and the US PAH Biobank Centers approved the validation cohort studies, and written informed consent was obtained at enrollment.

## Results

### Characterization of Study Cohorts and Tag Definition

Whole-genome sequencing was performed in 13 037 participants of the NBR study, of which 1148 were recruited to the PAH domain.^10^ The PAH domain included 23 unaffected parents and 3 cases with an unknown phenotype, which were removed from the analysis (Figure 1B). Of the remaining 1122 participants, 972 (86.6%) had a clinical diagnosis of IPAH, 73 (6.5%) of heritable PAH, and 20 (1.8%) were diagnosed with pulmonary veno-occlusive disease (PVOD)/pulmonary capillary hemangiomatosis (PCH). Diagnosis verification revealed that 57 participants (5%) had a diagnosis other than IPAH, heritable PAH, or PVOD/PCH. These cases were subsequently relabelled and moved to the respective tag group for analysis (Table 1). The comprehensive clinical characterisation of the study cohort is shown in Table I in the Data Supplement. In summary, the median age at diagnosis was 49 [35;63] years with a female predominance of 68%. Europeans constituted 84% of the study cohort. Overall survival in the studied population was 97% at one year, 91% at 3 years, and 84% at 5 years. As expected, there was a significant difference in survival between prevalent and incident cases. In prevalent cases, survival at 1, 3, and 5 years was 98%, 93%, and 87%, whereas in incident cases it was 97%, 84%, and 72%, respectively. Median transfer coefficient for carbon monoxide (KCO) in the entire studied population was 71 [52;86]% predicted. Cases in the lower tertile or below the KCO threshold of 50% predicted were more commonly male, older at diagnosis, had a current or past history of cigarette smoking and an increased number of cardiorespiratory comorbidities (Tables II, III, and IV in the Data Supplement). Survival in these groups was significantly worse than in those with preserved or mildly reduced KCO (Figure IA through ID in the Data Supplement). After adjusting for confounding factors (age, sex, comorbidities, smoking status and whether the case was prevalent or incident), KCO remained an independent predictor of survival (Table V in the Data Supplement).

Age at diagnosis was calculated as age at the time of diagnostic right heart catheterisation and was available in all but 10 cases. Patients in the higher age tertile showed more functional impairment despite milder hemodynamics, lower forced expiratory volume in one second/forced vital capacity ratio, and KCO (% predicted), as well as mild emphysematous and fibrotic changes on computerized tomography (CT scans; Figure IE and IF and Table VI in the Data Supplement).

### Rare Variants in Previously Established Genes

We identified variants in previously established genes (namely, *BMPR2*, *ACVRL1*, *ENG*, *SMAD1*, *SMAD4*, *SMAD9*, *KCNK3*, *TBX4*, *EIF2AK4*, *AQP1*, *ATP13A3*, *GDF2*, *SOX17*) in 271 (24.2%) of the 1122 cases and interpreted them based on the American College of Medical Genetics and Genomics standards and guidelines.^13^ The majority of these variants have already been described in Gräf et al^9^ (Material in the Data Supplement).

### Rare Variant Association Testing

**Figure 2.**
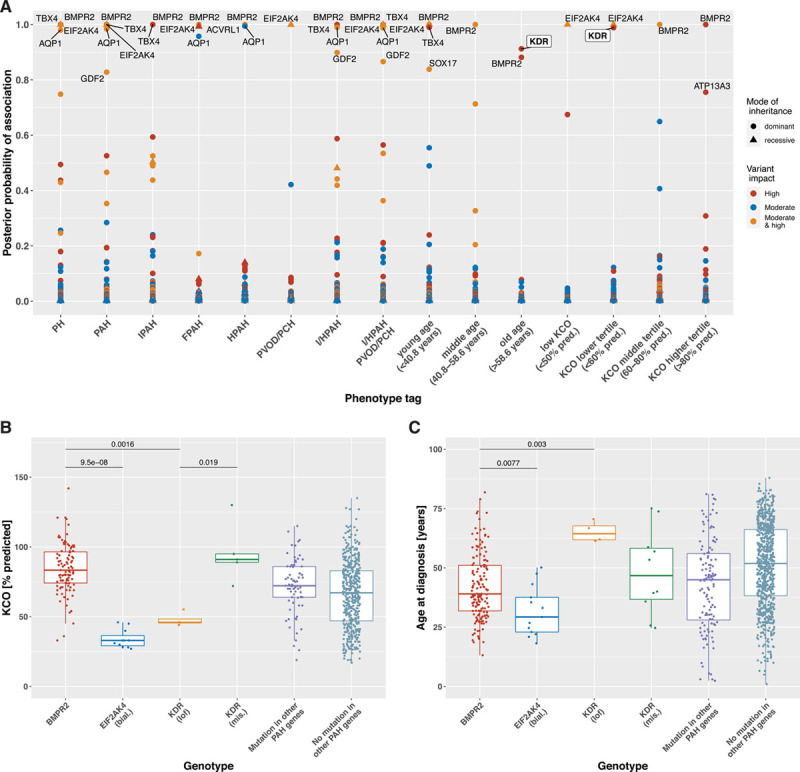
**Rare variant association study results revealing established and novel genotype-phenotype links.** **A**, Figure showing phenotype tags on the *x* axis and corresponding posterior probability of genotype-phenotype association on the *y* axis, as calculated by BeviMed. The definitions of the tags are listed in Table 1. Shape and colour of points indicate the mode of inheritance and impact/consequence type of variants driving the association. Box-and-whisker plots showing the distribution of (**B**) the transfer coefficient for carbon monoxide (KCO) and (**C**) the age at diagnosis stratified by genotype across the pulmonary arterial hypertension (PAH) domain. The 2-tailed Wilcoxon signed-rank test was used to determine differences in the medians of the distributions, which are indicated by the bars at the **top** of the figures providing the respective *P* values. *ACVRL1* indicates activin-like kinase 1; *AQP1*, aquaporin 1; bial, biallelic; *BMPR2*, bone morphogenetic protein receptor type 2; *EIF2AK4*, eukaryotic translation initiation factor 2 alpha kinase 4; FPAH, familial PAH; *GDF2*, growth differentiation factor 2; I/HPAH, idiopathic/hereditary pulmonary arterial hypertension; lof, loss-of-function; mis, missense; PAH, pulmonary arterial hypertension; PH, pulmonary hypertension; PVOD/PCH, pulmonary veno-occlusive disease/pulmonary capillary hemangiomatosis; and *TBX4*, T-box transcription factor 4.

We used Bayesian methodology to consolidate previously reported PAH genes and to discover novel genotype-phenotype associations. Of note, cases explained by rare deleterious variants in previously established genes were only included for the association testing with the respective disease gene (Methods in the Data Supplement). This analysis identified 40 significant gene-tag associations with posterior probability (PP) above 0.75 (Table 2 and Figure 2A). *BMPR2*, *TBX4*, *EIF2AK4*, *ACVRL1*, and *AQP1* showed the highest association (PP ≥0.99), but we also confirmed significant associations in the majority of other previously identified genes. Individuals with rare variants in *BMPR2*, *TBX4* (high impact), *EIF2AK4* (biallelic), and *SOX17* had a significantly younger age of disease onset (tag: young age). We also confirmed the association of rare variants in *AQP1* with familial PAH (log [BF]=10.023, PP=0.958). The refined phenotype approach corroborated the association between high impact variants in *BMPR2* and preserved KCO (KCO higher tertile, log [BF]=99.923, PP=1) together with an association of biallelic *EIF2AK4* mutations with significantly reduced KCO (KCO <50% predicted, log [BF]=29.741, PP=1).

**Table 2.**
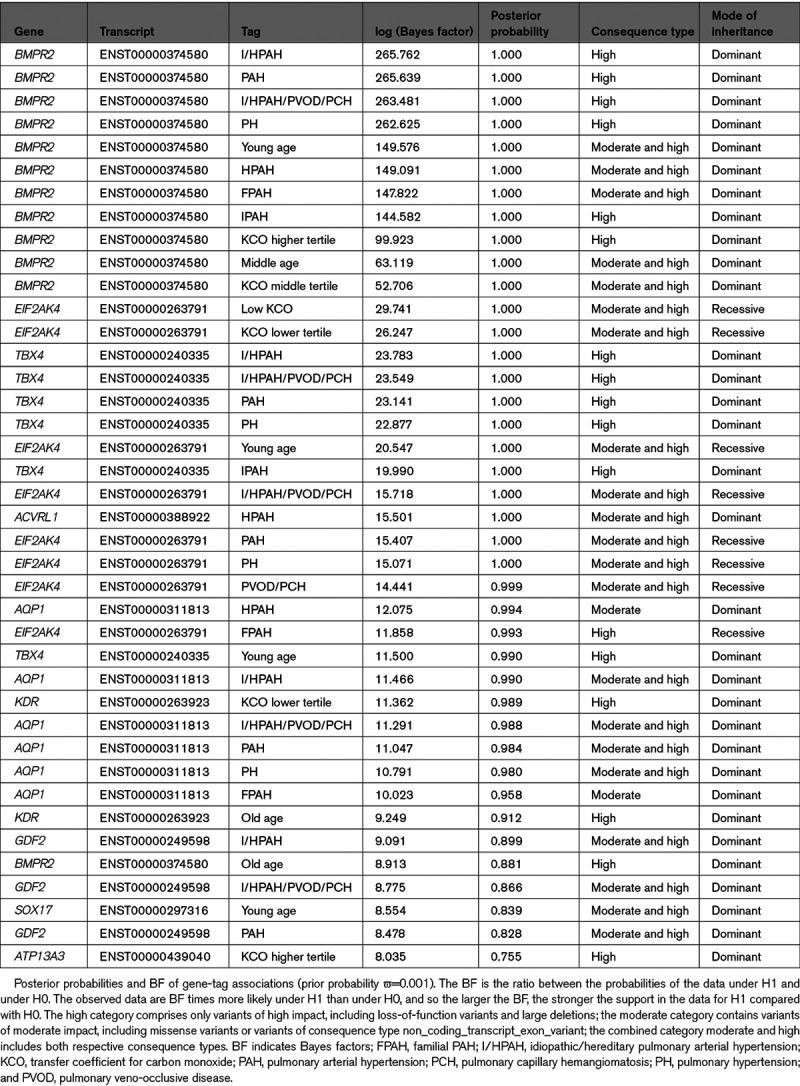
BeviMed Analysis Results

**Table 1.**
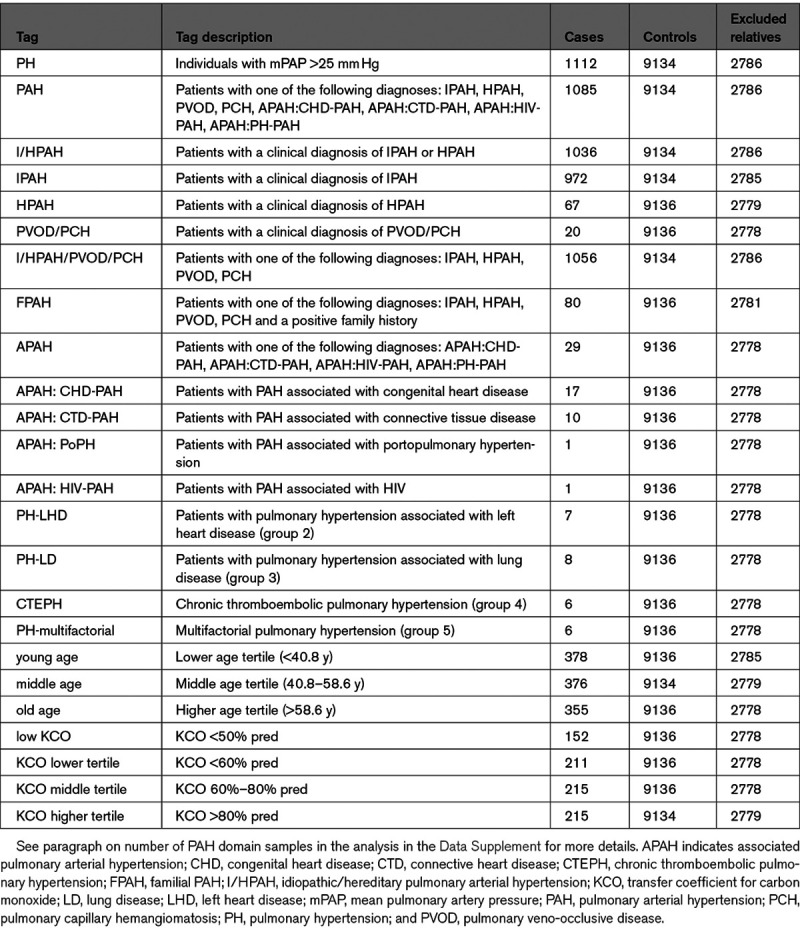
Definitions of Labels and the Number of Unrelated Cases and Controls in the Rare Variant Association Analysis With BeviMed

Under an autosomal dominant mode of inheritance, high impact variants in the kinase insert domain receptor (*KDR*) were associated with a significantly reduced KCO (KCO lower tertile, log [BF]=11.362, PP=0.989) and older age at diagnosis (tag: old age, log [BF]=9.249, PP=0.912).

### Rare High Impact Variants in the New PAH Candidate Gene *KDR*

**Figure 3.**
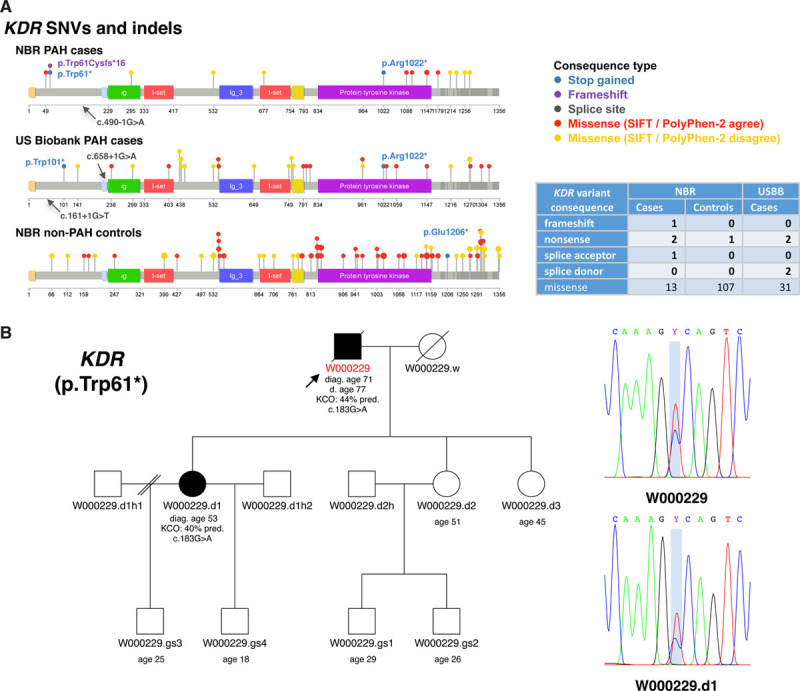
**Summary of rare single nucleotide variants (SNVs) and small insertions and deletions (indels) identified in the novel pulmonary arterial hypertension (PAH) candidate gene KDR (kinase insert domain receptor).** **A**, Only rare predicted deleterious variants in KDR are shown (minor allele frequency [MAF] <1/10 000 and combined annotation dependent depletion [CADD] ≥10). SNVs and indels are represented by colored lollipops on **top** of the protein sequence. The domain annotations were retrieved from Uniprot (accession number P35968). Lollipop colors indicate the consequence type, and sizes represent the variant frequency within a cohort. Missense variants that are predicted to be deleterious (Sorting Intolerant From Tolerant prediction score [SIFT]) and damaging (PolyPhen-2) are colored in red, otherwise in yellow (ie, SIFT and PolyPhen-2 disagree). High impact variants are labelled with the respective Human Genome Variation Society notation. The number of variants by predicted consequence type and cohort is provided in the table. **B**, Familial segregation of KDR nonsense variant c.183G>A (p.Trp61*) with PAH (ie, reduced KCO and late onset) from father (W000229) to daughter (W000229.d). Sanger sequencing results are shown in the chromatograms. NBR indicates NIHR BioResource—Rare Diseases; and USBB, US PAH Biobank.

We identified 5 ultrarare high impact variants in *KDR* in the study cohort. Ultrarare variants exist in the general population only at a frequency of <1 in 10 000 (0.01%). Four of these were in PAH cases: one frameshift variant in exon 3 of 30 (c.183del, p.Tryp61CysfsTer16), 2 nonsense variants, one in exon 3 (c.183G>A, p.Trp61Ter) and one in exon 22 (c.3064C>T, p.Arg1022Ter), and one splice acceptor variant in intron 4 of 29 (c.490-1G>A). In addition, one nonsense variant was identified in exon 27 (p.Glu1206Ter) in a non-PAH control subject (Table 3). This latter nonsense variant appears late in the amino acid sequence, in exon 27 of 30, and hence is likely to escape nonsense-mediated decay, but this remains to be studied functionally. All loss-of-function variants were confirmed by Sanger sequencing (Figure 3 and Figure II in the Data Supplement). Furthermore, 13 PAH cases (1%) and 108 non-PAH controls (0.9%) harbored rare, predicted-deleterious *KDR* missense variants of moderate impact (Figure 3). The missense variant carriers, however, did not exhibit a reduced KCO or older age at diagnosis. Instead, these patients show the opposite trend in KCO (Figure 2B and 2C). Importantly, 7 of the 13 *KDR* missense variants seen in PAH cases were also detected in several non-PAH controls and thus, are of unknown significance. Furthermore, 3 of these missense variants co-occurred with a predicted-deleterious variant in an established PAH risk gene (2 patients carried also a variant in *BMPR2* and one a variant in *AQP1*).

**Table 3.**
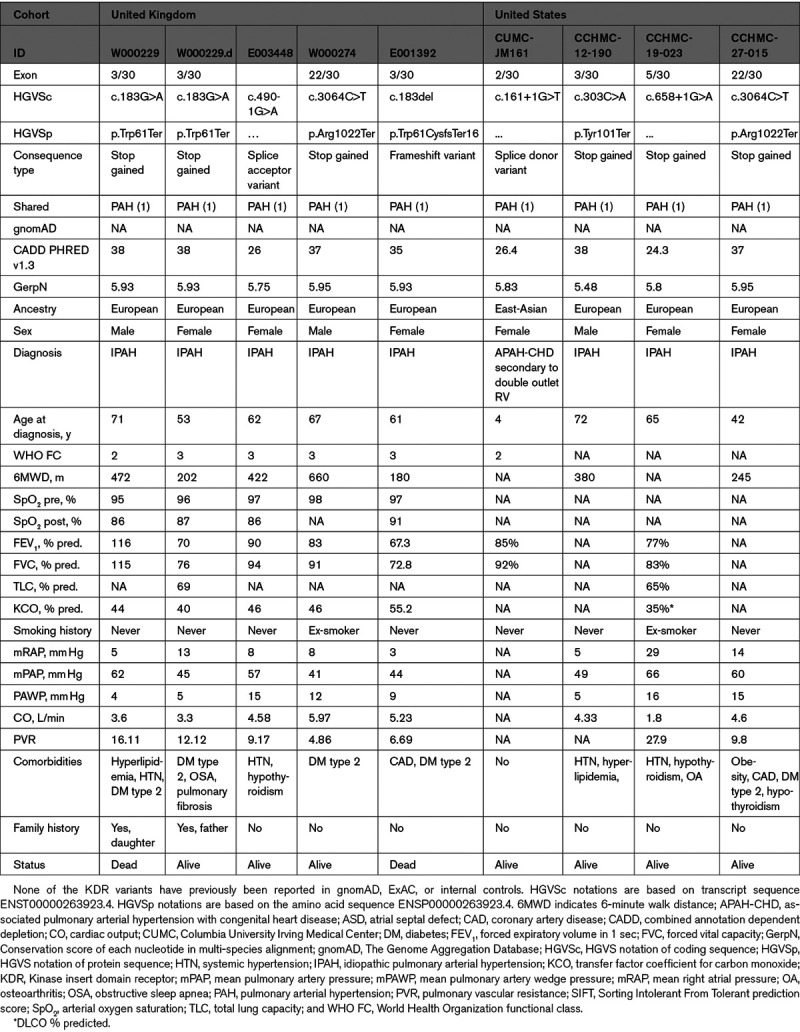
Gene Changes for Patients With IPAH Harboring Likely Loss-of-Function Variants in the KDR Gene

### Clinical Characterization of *KDR* Mutation Carriers

**Figure 4.**
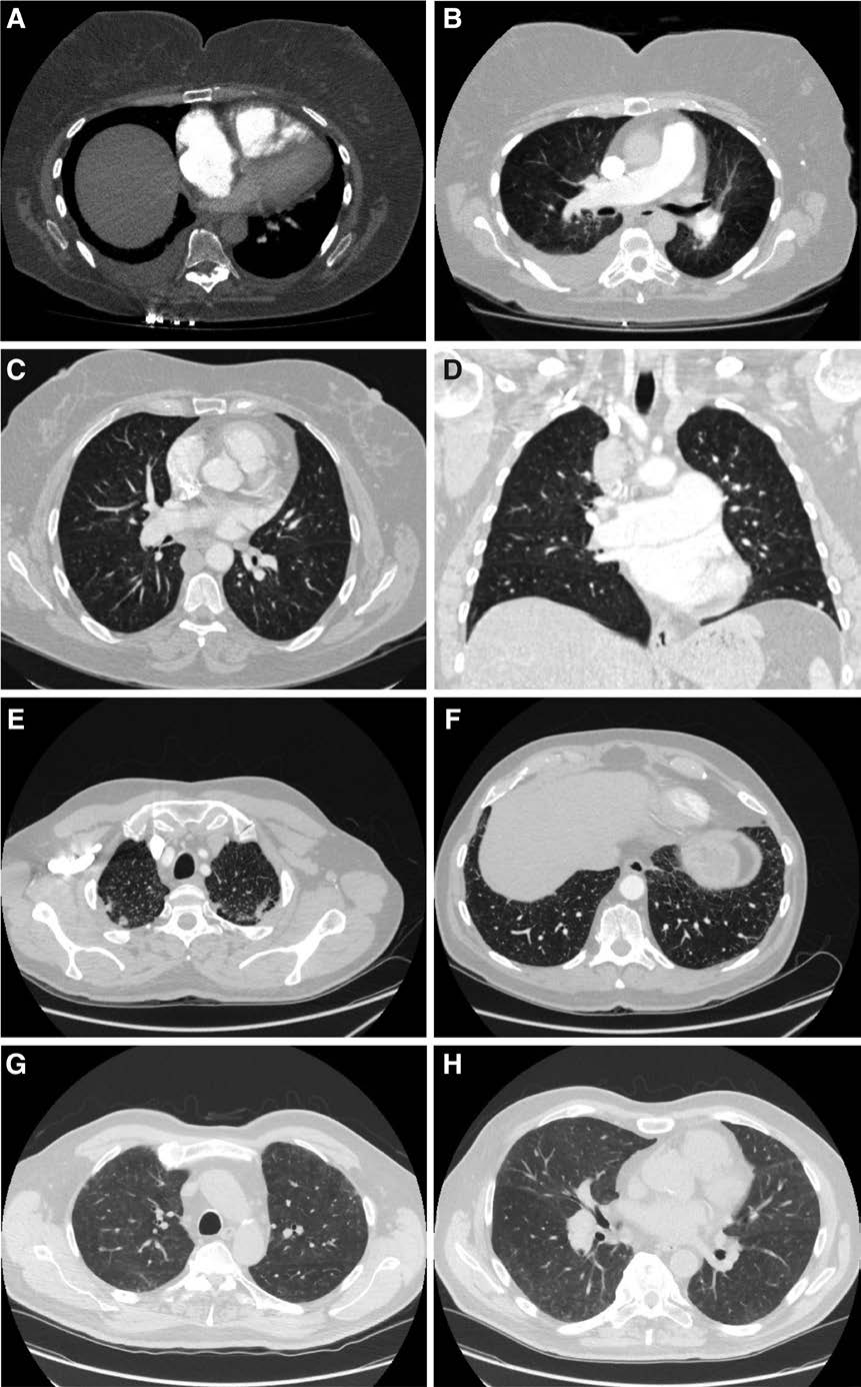
**Chest computerized tomography (CT) scans of patients carrying high impact kinase insert domain receptor (*KDR*) mutations.** **A**, Axial image of CT pulmonary angiogram at the level of the right ventricle (RV) moderator band, showing flattening of interventricular septum, leftwards bowing of the interatrial septum and the enlargement of the right atrium (RA) and RV, indicative of RV strain; bilateral pleural effusion, larger on the right side. **B**, Axial image of a pulmonary CT angiogram demonstrating enlarged pulmonary artery and mild central lung ground-glass opacity (GGO). **C**, Axial high-resolution CT slice of the chest in the lung window showing a trace of non-specific GGO with a central distribution. **D**, Coronal image showing the trace of central GGO and enlarged central pulmonary arteries. Axial high-resolution CT slice of the chest in the lung window showing apical subpleural fibrosis (**E**), and very minor subpleural fibrosis at the lung bases (**F**). Axial high-resolution CT slice of the chest in the lung window showing subpleural GGO at apical level (**G**), and mild GGO at mid-thoracic level (**H**). Patients: E001392 (**A** and **B**), E003448 (**C** and **D**), W000229 (**E** and **F**), W000274 (**G** and **H**).

Patients with high impact variants in *KDR* were older and exhibited significantly reduced KCO similar to biallelic *EIF2AK4* mutation carriers and in contrast to *KDR* missense variant and *BMPR2* mutation carriers (Figure 2B and 2C). Three of the 4 cases did not have a history of smoking. CT scans for all 4 patients showed a range of mild lung parenchymal changes (Figure 4). W000229 had evidence of mild mainly subpleural interstitial lung disease (ILD), mild emphysema, and air trapping. W000274 had signs of ILD with traction bronchiectasis in the lower zones, mild air trapping, and mild diffuse ground-glass opacities and neovascularity. E001392 showed mild centrilobular ground-glass opacities in addition to moderate pleural effusion and a trace of air trapping, but no ILD. In these cases, it seemed likely that the observed parenchymal changes contributed to the low KCO. In contrast, E003448 had a low KCO despite only a trace of central nonspecific ground-glass opacities on the CT images. Comparisons of CT findings between patients harboring deleterious mutations in *BMPR2*, *EIF2AK4*, *KDR*, other PAH risk genes and patients without mutations are presented in Table VII in the Data Supplement. There were no differences in the frequency of comorbidities between patients harboring missense and loss-of-function variants in *KDR* although the frequency of systemic hypertension was high (44%; Table VIII in the Data Supplement). Survival analysis could not be conducted due to the small number of mutation carriers, as well as only 2 events occurring in this group. Following the death of W000229, his daughter, aged 53, was diagnosed with PAH and had a reduced KCO at 40% predicted. On the CT scan, mild interstitial fibrosis was observed (Figure III in the Data Supplement). Sanger sequencing confirmed that father and daughter carried the same deleterious *KDR* nonsense variant p.Trp61Ter (Figure 3B).

### Additional *KDR* Cases in US PAH Cohorts

To seek further evidence for *KDR* as a new candidate gene for PAH, we analyzed subjects recruited to the US PAH Biobank^12^ and the Columbia University Irving Medical Center^14^ to identify additional patients carrying predicted pathogenic rare variants. Four additional individuals harboring rare high impact *KDR* variants were identified. These comprised, 2 nonsense variants, one in exon 3 (c.303C>A, p.Tyr101Ter) and one in exon 22 (c.3064C>T, p.Arg1022Ter) and 2 splice donor variants, one in intron 2 of 29 (c.161+1G>T) and one in intron 5 (c.658+1G>A). Interestingly, the nonsense variant p.Arg1022Ter appeared in both cohorts (Figure 3). Patient-level data for these individuals are summarized in Table 3. Three of the 4 patients were diagnosed with IPAH at 72, 65, and 42 years, respectively, whereas one patient was diagnosed at age 4 with PAH associated with double outlet right ventricle. The diffusing capacity of carbon monoxide was available for one patient and was decreased at 35% predicted, with minor pleural scarring in the left upper lobe found on CT imaging. Two out of 4 patients (50%) harboring a high impact variant in *KDR* had been diagnosed with systemic hypertension.

## Discussion

One of the critical steps in identifying novel, causative genes in rare disorders is the discovery of genotype-phenotype associations to inform patient care and outcomes. A pragmatic focus on deeply phenotyped individuals and smart experimental design provides additional leverage to identify novel risk variants.^15^ To deploy this approach in PAH, we brought together phenotypic and genetic data using Bayesian methodology.^11^ This Bayesian framework allows the inclusion of prior information regarding the hypothesis being tested in a flexible manner and compares a range of possible genetic models in a single analysis. To generate case-control labels, we tagged PAH cases with diagnostic labels and stratified them by age at diagnosis and KCO. Analyses were then performed to identify associations between tags and ultrarare gene variants under dominant and recessive modes of inheritance and different variant impact categories.

Our Bayesian methodology analysis provided strong statistical evidence of an association between ultrarare, high impact variants in *KDR* and PAH with significantly reduced KCO and older age at diagnosis under a dominant mode of inheritance. Strikingly, likely loss-of-function variants in *KDR* exist in the general population with a frequency of only 4 to 7 per 100 000 (see Table 4). In contrast, we identified 4 PAH cases in the NBR cohort which equates to almost 2 in 1000. Additionally, the statistical constraint metrics provided by Genome Aggregation Database^16^ strongly suggest that loss-of-function variants in *KDR* are not tolerated (pLI=1; o/e=0.15 [0.09;0.25]). Besides the statistical evidence, we also identified one additional case with a family history, which together with a recently published case report of 2 families in which loss-of-function variants in *KDR* segregated with PAH and significantly reduced KCO,^17^ amounts to 3 reported familial cases with a distinct phenotype.

**Table 4.**
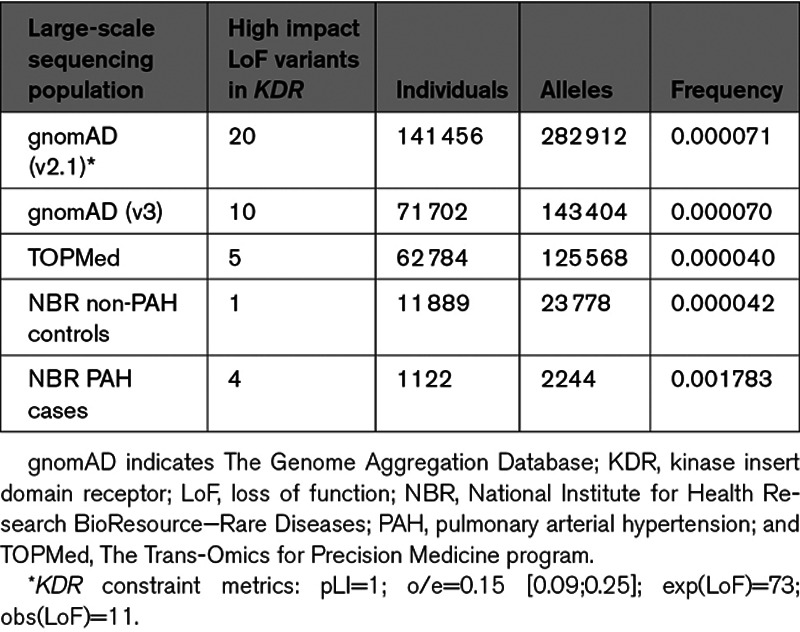
Comparison of High Impact Likely Loss-of-Function Variants in KDR in the Human Large-Scale Sequencing Reference Populations gnomAD and TOPMed With the NBR Non-PAH Controls and PAH Cases

Vascular endothelial growth factor receptor 2 (VEGFR2), which is encoded by *KDR*,^18^ binds VEGFA, a critical growth factor for physiological and pathological angiogenesis in vascular endothelial cells. In mice, even though *VegfA* haploinsufficiency is embryonically lethal,^19^ heterozygosity of its receptor, Vegfr2, is compatible with life and unperturbed vascular development.^20^ The role of VEGF signaling in the pathogenesis of PAH has been an area of intense interest since increased expression of VEGF, VEGFR1, and VEGFR2 were reported in rat lung tissue in response to acute and chronic hypoxia.^21^ An increase in lung VEGF has also been reported in rats with pulmonary hypertension (PH) following monocrotaline exposure.^22^ In humans, VEGF-A is highly expressed in plexiform lesions in patients with IPAH.^23^ In addition, inhibition of VEGF signaling by SU5416 (sugen) combined with chronic hypoxia triggers severe angioproliferative PH.^24^ SU5416, a small-molecule inhibitor of the tyrosine kinase segment of VEGF receptors, inhibits VEGFR1,^25^ and VEGFR2^26^ causing endothelial cell apoptosis, loss of lung capillaries, and emphysema.^27^ Further evidence supporting the role of VEGF inhibition in the pathobiology of PAH comes from reports of PH in patients treated with bevacizumab^28^ and the multi-tyrosine kinase inhibitors.^29,30^ Mutations in *KDR* have also been linked to congenital heart diseases. Bleyl et al^31^ reported that *KDR* might be a candidate for familial total anomalous pulmonary venous return. In addition, haploinsufficiency at the *KDR* locus has also been associated with tetralogy of Fallot.^32^ We identified one patient in the Columbia University Irving Medical Center cohort with PAH associated with congenital heart disease harboring a *KDR* likely protein-truncating splice donor variant (c.161+1G>T).

In the present study, we highlight that deep clinical phenotyping, in combination with genotype data, can improve the identification of novel disease risk genes and disease subtypes. *KDR* was already identified as a possible candidate gene, which did not achieve genome-wide significance, in our previous rare variant association study.^9^ In combination with deep phenotyping data, *KDR* reached in the present study a significance level comparable to the most commonly affected genes in PAH. Reduced KCO, which reflects impairment of alveolar-capillary membrane function, has been noted in the analysis of early PAH registry data^33^ to be an independent predictor of survival. Decreased KCO was also found in patients with PVOD/PCH with or without biallelic *EIF2AK4* mutations.^34^ Although some reduction in KCO is one of the typical features of pulmonary vascular disease, patients with PVOD show the lowest KCO values when compared with IPAH or chronic thromboembolic pulmonary hypertension. In contrast, KCO is relatively preserved in *BMPR2* mutation carriers.^35^ Strong association with survival and a link with other causative mutations makes the KCO phenotype particularly attractive for stratification in genetic studies.

As lung disease should always be taken under consideration as a cause of low KCO, we applied the World Symposium on PH criteria^36^ to exclude lung disease as a cause of PH: total lung capacity ≥70% pred., forced vital capacity ≥70% pred., forced expiratory volume in one second ≥60% pred., and no severe fibrosis and emphysema on chest CT. None of the cases carrying a high impact variant in *KDR* met these criteria, although 2 of the 4 patients did show evidence of early ILD. Another potential reason for low KCO in the PAH population is the diagnosis of PVOD/PCH.^37,38^ Careful analysis of CT scans and clinical data did not reveal convincing evidence for this diagnosis in *KDR* mutation carriers. Cigarette smoking is a well-known factor leading to the decrease of KCO. Only one of the 4 *KDR* high impact variant carriers had a significant 15 pack-years smoking history, but with no signs of emphysema on CT. These findings suggest that loss-of-function variants in *KDR* are associated with a form of PAH characterized by a range of lung parenchymal abnormalities, including small airways disease, emphysema and ILD, as 2 of the 4 patients harboring a high impact variant in *KDR* had mild fibrotic lung changes. Notably, patients with mutations in other PAH risk genes, or those without the identified genetic mutation, showed <10% incidence of fibrotic changes on CT imaging. Further larger studies are needed to determine the full range of lung parenchymal abnormalities in PAH cases with deleterious variants in *KDR*.

In this study, we have assumed that PAH is a monogenic condition, which is caused by either deleterious heterozygous or biallelic variants in a single gene. This assumption, although widely supported by the literature, may not be entirely accurate. Alternatively, some cases of PAH might represent an oligogenic inheritance involving 2 or more genes. Although not statistically explored in the current analysis, we found a total of 22 PAH cases carrying deleterious variants in more than one PAH gene. These variants could contribute as genetic modifiers, impacting penetrance and/or expressivity. In this analysis, we have explored only a limited number of clinical phenotypes. Further studies with larger numbers of phenotypic tags derived from clinical and molecular data will increase the power to detect new associations. Finally, KCO measurements were missing for a proportion of patients which could introduce a selection bias, although all the deleterious variants in *KDR* had phenotypic data available in the UK cohort.

In summary, this study shows that deep phenotyping enables patient stratification into subgroups with shared pathobiology and with increased power to detect new genotype-phenotype associations. We provide statistical evidence for an association between high impact, likely loss-of-function variants in *KDR* and significantly decreased KCO and later disease onset, further supported by familial segregation.

## Acknowledgments

We thank National Institute for Health Research (NIHR) BioResource volunteers for their participation, and gratefully acknowledge NIHR BioResource centers, NHS Trusts and staff for their contribution. We thank the National Institute for Health Research and NHS Blood and Transplant. The views expressed are those of the author(s) and not necessarily those of the NHS, the NIHR or the Department of Health and Social Care. We thank the research nurses and coordinators at the specialist pulmonary hypertension centers involved in this study. We acknowledge the support of the Imperial NIHR Clinical Research Facility, the Netherlands CardioVascular Research Initiative, the Dutch Heart Foundation, Dutch Federation of University Medical Centres, the Netherlands Organization for Health Research and Development and the Royal Netherlands Academy of Sciences. We thank all the patients and their families who contributed to this research and the Pulmonary Hypertension Association (United Kingdom) for their support. We also thank Kathryn Auckland for proofreading the article. We thank contributors, including the Pulmonary Hypertension Centers who collected samples used in this study, as well as patients and their families, whose help and participation made this work possible. Exome sequencing and genotyping data were generated by the Regeneron Genetics Center.

## Sources of Funding

The UK National Cohort of Idiopathic and Heritable Pulmonary Arterial Hypertension (PAH) is supported by the National Institute for Health Research (NIHR), the British Heart Foundation (BHF; SP/12/12/29836 and SP/18/10/33975), the BHF Cambridge Centre of Cardiovascular Research Excellence, and the UK Medical Research Council (MR/K020919/1), the Dinosaur Trust, BHF Programme grants to R.C. Trembath (RG/08/006/25302) and Dr Morrell (RG/13/4/30107), and the UK NIHR National Institute for Health Research Cambridge Biomedical Research Centre. Dr Morrell is a BHF Professor and NIHR Senior Investigator.Dr Lawrie is supported by a BHF Senior Basic Science Research Fellowship (FS/13/48/30453). All research at Great Ormond Street Hospital NHS Foundation Trust and UCL Great Ormond Street Institute of Child Health is made possible by the NIHR Great Ormond Street Hospital Biomedical Research Centre. Samples and data from the National Biological Sample and Data Repository for PAH, funded by an NIH investigator-initiated resources grant (R24 HL105333 to Dr Nichols), were used in this study.

## Disclosures

Dr Morrell is a Director and Co-founder of Morphogen-IX. Dr Wharton received personal fees from Actelion Pharmaceuticals. Dr Kovacs reports personal fees and nonfinancial support from Actelion Pharmaceuticals, Bayer, GlaxoSmithKline, Merck Sharp & Dohme Corp., Boehringer Ingelheim, Novartis, Chiesi, and Vitalaire outside the submitted work. Dr Penkett declares fees from Actelion Pharmaceuticals and United Therapeutics. Dr Lawrie received support and fees from GlaxoSmithKline and Actelion Pharmaceuticals.

## Supplementary Material



## Appendix

### NIHR BioResource for Translational Research-Rare Diseases/National Cohort Study of Idiopathic and Heritable PAH collabor

Stephen Abbs, Lara Abulhoul, Julian Adlard, Munaza Ahmed, Timothy J. Aitman, Hana Alachkar, David J. Allsup, Philip Ancliff, Richard Antrobus, Ruth Armstrong, Gavin Arno, Sofie Ashford, William J. Astle, Anthony Attwood, Paul Aurora, Christian Babbs, Chiara Bacchelli, Tamam Bakchoul, Siddharth Banka, Tadbir Bariana, Julian Barwell, Joana Batista, Helen E. Baxendale, Phil L. Beales, David L. Bennett, Agnieszka Bierzynska, Tina Biss, Maria A.K. Bitner-Glindzicz, Graeme C. Black, Marta Bleda, Iulia Blesneac, Detlef Bockenhauer, Harm Bogaard, Sara Boyce, John R. Bradley, Gerome Breen, Paul Brennan, Carole Brewer, Matthew Brown, Andrew C. Browning, Michael J. Browning, Rachel J. Buchan, Matthew S. Buckland, Teofila Bueser, Carmen Bugarin Diz, John Burn, Siobhan O. Burns, Oliver S. Burren, Nigel Burrows, Carolyn Campbell, Gerald Carr-White, Keren Carss, Ruth Casey, Mark J. Caulfield, Jenny Chambers, John Chambers, Melanie M.Y. Chan, Floria Cheng, Patrick F. Chinnery, Manali Chitre, Martin T. Christian, Colin Church, Jill Clayton-Smith, Maureen Cleary, Naomi Clements Brod, Gerry Coghlan, Elizabeth Colby, Trevor R.P. Cole, Janine Collins, Peter W. Collins, Cecilia J. Compton, Robin Condliffe, H. Terence Cook, Stuart Cook, Nichola Cooper, Paul A. Corris, Nicola S. Curry, Matthew J. Daniels, Mehul Dattani, Louise C. Daugherty, John Davis, Anthony De Soyza, Sri V.V. Deevi, Timothy Dent, Charu Deshpande, Eleanor F. Dewhurst, Peter H. Dixon, Sofia Douzgou, Kate Downes, Anna M. Drazyk, Elizabeth Drewe, Daniel Duarte, Tina Dutt, J. David M. Edgar, Karen Edwards, William Egner, Melanie N. Ekani, Perry Elliott, Wendy N. Erber, Marie Erwood, Maria C. Estiu, Dafydd Gareth Evans, Gillian Evans, Tamara Everington, Mèlanie Eyries, Hiva Fassihi, Remi Favier, Debra Fletcher, Frances A. Flinter, R. Andres Floto, Tom Fowler, James Fox, Amy J. Frary, Courtney E. French, Kathleen Freson, Mattia Frontini, Abigail Furnell, Daniel P. Gale, Henning Gall, Vijeya Ganesan, Michael Gattens, Stefano Ghio, Hossein-Ardeschir Ghofrani, J. Simon R. Gibbs, Kate Gibson, Kimberly C. Gilmour, Barbara Girerd, Nicholas S. Gleadall, Sarah Goddard, Keith Gomez, Pavels Gordins, David Gosal, Stefan Gräf, Jodie Graham, Luigi Grassi, Daniel Greene, Lynn Greenhalgh, Andreas Greinacher, Paolo Gresele, Philip Griffiths, Sofia Grigoriadou, Detelina Grozeva, Mark Gurnell, Scott Hackett, Charaka Hadinnapola, Rosie Hague, William M. Hague, Matthias Haimel, Matthew Hall, Helen L. Hanson, Eshika Haque, Kirsty Harkness, Andrew R. Harper, Claire L. Harris, Daniel Hart, Ahamad Hassan, Grant Hayman, Alex Henderson, Archana Herwadkar, Jonathan Hoffman, Simon Holden, Rita Horvath, Henry Houlden, Arjan C. Houweling, Luke S. Howard, Fengyuan Hu, Gavin Hudson, Aarnoud P. Huissoon, Marc Humbert, Matthew Hurles, Melita Irving, Louise Izatt, Roger James, Sally A. Johnson, Stephen Jolles, Jennifer Jolley, Dragana Josifova, Neringa Jurkute, Mary A. Kasanicki, Hanadi Kazkaz, Rashid Kazmi, Peter Kelleher, Anne M Kelly, Wilf Kelsall, Carly Kempster, David G Kiely, Nathalie Kingston, Nils Koelling, Myrto Kostadima, Gabor Kovacs, Ania Koziell, Roman Kreuzhuber, Taco W. Kuijpers, Ajith Kumar, Dinakantha Kumararatne, Manju A. Kurian, Michael A. Laffan, Fiona Lalloo, Michele Lambert, Hana Lango Allen, Allan Lawrie, D. Mark Layton, Claire Lentaigne, Tracy Lester, Adam P. Levine, Rachel Linger, Hilary Longhurst, Lorena E. Lorenzo, Eleni Louka, Paul A. Lyons, Rajiv D. Machado, Robert V. MacKenzie Ross, Bella Madan, Eamonn R. Maher, Jesmeen Maimaris, Samantha Malka, Sarah Mangles, Rutendo Mapeta, Kevin J. Marchbank, Stephen Marks, Hugh S. Markus, Hanns-Ulrich Marschall, Andrew Marshall, Jennifer Martin, Mary Mathias, Emma Matthews, Heather Maxwell, Paul McAlinden, Mark I. McCarthy, Harriet McKinney, Stuart Meacham, Adam J. Mead, Karyn Megy, Sarju G. Mehta, Michel Michaelides, Carolyn Millar, Shehla N. Mohammed, Shahin Moledina, David Montani, Anthony T. Moore, Nicholas W. Morrell, Monika Mozere, Keith W. Muir, Andrew D. Mumford, Andrea H. Nemeth, William G. Newman, Michael Newnham, Sadia Noorani, Paquita Nurden, Jennifer O'Sullivan, Samya Obaji, Chris Odhams, Steven Okoli, Andrea Olschewski, Horst Olschewski, Kai Ren Ong, S. Helen Oram, Elizabeth Ormondroyd, Willem H. Ouwehand, Claire Palles, Sofia Papadia, Soo-Mi Park, David Parry, Smita Patel, Joan Paterson, Andrew Peacock, Simon H. Pearce, Kathelijne Peerlinck, Christopher J. Penkett, Joanna Pepke-Zaba, Romina Petersen, Clarissa Pilkington, Kenneth E.S. Poole, Bethan Psaila, Angela Pyle, Richard Quinton, Shamima Rahman, Anupama Rao, F. Lucy Raymond, Paula J. Rayner-Matthews, Augusto Rendon, Tara Renton, Christopher J. Rhodes, Andrew S.C. Rice, Alex Richter, Leema Robert, Irene Roberts, Sarah J. Rose, Robert Ross-Russell, Catherine Roughley, Noemi B.A. Roy, Deborah M. Ruddy, Omid Sadeghi-Alavijeh, Moin A. Saleem, Nilesh Samani, Crina Samarghitean, Alba Sanchis-Juan, Ravishankar B. Sargur, Robert N. Sarkany, Simon Satchell, Sinisa Savic, Genevieve Sayer, John A. Sayer, Laura Scelsi, Andrew M. Schaefer, Sol Schulman, Richard Scott, Marie Scully, Claire Searle, Werner Seeger, Arjune Sen, W.A. Carrock Sewell, Denis Seyres, Neil Shah, Olga Shamardina, Susan E. Shapiro, Adam C. Shaw, Keith Sibson, Lucy Side, Ilenia Simeoni, Michael A. Simpson, Matthew C. Sims, Suthesh Sivapalaratnam, Damian Smedley, Katherine R. Smith, Kenneth G.C. Smith, Katie Snape, Nicole Soranzo, Florent Soubrier, Laura Southgate, Olivera Spasic- Boskovic, Simon Staines, Emily Staples, Hannah Stark, Jonathan Stephens, Kathleen E. Stirrups, Alex Stuckey, Jay Suntharalingam, Emilia M. Swietlik, Petros Syrris, R. Campbell Tait, Kate Talks, Rhea Y.Y. Tan, Jenny C. Taylor, John M. Taylor, James E. Thaventhiran, Andreas C. Themistocleous, David Thomas, Ellen Thomas, Moira J. Thomas, Patrick Thomas, Kate Thomson, Adrian J. Thrasher, Chantal Thys, Tobias Tilly, Marc Tischkowitz, Catherine Titterton, Cheng-Hock Toh, Ian P. Tomlinson, Mark Toshner, Matthew Traylor, Carmen Treacy, Paul Treadaway, Richard Trembath, Salih Tuna, Ernest Turro, Philip Twiss, Tom Vale, Chris Van Geet, Natalie van Zuydam, Anthony M Vandersteen, Marta Vazquez-Lopez, Julie von Ziegenweidt, Anton Vonk Noordegraaf, Annette Wagner, Quinten Waisfisz, Neil Walker, Suellen M. Walker, James S. Ware, Hugh Watkins, Christopher Watt, Andrew R. Webster, Lucy Wedderburn, Wei Wei, Steven B. Welch, Julie Wessels, Sarah K. Westbury, John-Paul Westwood, John Wharton, Deborah Whitehorn, James Whitworth, Andrew O.M. Wilkie, Martin R. Wilkins, Catherine Williamson, Brian T. Wilson, Edwin K.S. Wong, Nicholas Wood, Yvette Wood, Christopher Geoffrey Woods, Emma R. Woodward, Stephen J. Wort, Austen Worth, Michael Wright, Katherine Yates, Patrick F.K. Yong, Timothy Young, Ping Yu, Patrick Yu-Wai-Man, Eliska Zlamalova: University of Cambridge & Cambridge University Hospitals NHS Foundation Trust; www.ipahcohort.com, Cambridge, United Kingdom.

### PAH Biobank Enrolling Centers' Investigators

Russel Hirsch, MD; R. James White, MD, PhD; Marc Simon, MD; David Badesch, MD; Erika Rosenzweig, MD; Charles Burger, MD; Murali Chakinala, MD; Thenappan Thenappan, MD; Greg Elliott, MD; Robert Simms, MD; Harrison Farber, MD; Robert Frantz, MD; Jean Elwing, MD; Nicholas Hill, MD; Dunbar Ivy, MD; James Klinger, MD; Steven Nathan, MD; Ronald Oudiz, MD; Ivan Robbins, MD; Robert Schilz, DO, PhD; Terry Fortin, MD; Jeffrey Wilt, MD; Delphine Yung, MD; Eric Austin, MD; Ferhaan Ahmad, MD, PhD; Nitin Bhatt, MD; Tim Lahm, MD; Adaani Frost, MD; Zeenat Safdar, MD; Zia Rehman, MD; Robert Walter, MD; Fernando Torres, MD; Sahil Bakshi, DO; Stephen Archer, MD; Rahul Argula, MD; Christopher Barnett, MD; Raymond Benza, MD; Ankit Desai, MD; Veeranna Maddipati, MD: www.pahbiobank.org, Cincinnati, OH.

## References

[1] Tuder RM Groves B Badesch DB Voelkel NF Exuberant endothelial cell growth and elements of inflammation are present in plexiform lesions of pulmonary hypertension. Am J Pathol. 1994;144:275–285. 7508683 PMC1887146

[2] Pullamsetti SS Savai R Seeger W Goncharova EA Translational advances in the field of pulmonary hypertension. From cancer biology to new pulmonary arterial hypertension therapeutics. Targeting cell growth and proliferation signaling hubs. Am J Respir Crit Care Med. 2017;195:425–437. doi: 10.1164/rccm.201606-1226PP 27627135 10.1164/rccm.201606-1226PP PMC5803657

[3] Lane KB Machado RD Pauciulo MW Thomson JR Phillips JA III Loyd JE Nichols WC Trembath RC; International PPH Consortium. Heterozygous germline mutations in BMPR2, encoding a TGF-beta receptor, cause familial primary pulmonary hypertension Nat Genet. 2000;26:81–84. 10973254 10.1038/79226

[4] Trembath RC Mutations in the TGF-beta type 1 receptor, ALK1, in combined primary pulmonary hypertension and hereditary haemorrhagic telangiectasia, implies pathway specificity. J Heart Lung Transplant. 2001;20:175 doi: 10.1016/s1053-2498(00)00352-1 10.1016/s1053-2498(00)00352-1 11250282

[5] Chaouat A Coulet F Favre C Simonneau G Weitzenblum E Soubrier F Humbert M Endoglin germline mutation in a patient with hereditary haemorrhagic telangiectasia and dexfenfluramine associated pulmonary arterial hypertension. Thorax. 2004;59:446–448. doi: 10.1136/thx.2003.11890 15115879 10.1136/thx.2003.11890 PMC1746994

[6] Shintani M Yagi H Nakayama T Saji T Matsuoka R A new nonsense mutation of SMAD8 associated with pulmonary arterial hypertension. J Med Genet. 2009;46:331–337. doi: 10.1136/jmg.2008.062703 19211612 10.1136/jmg.2008.062703

[7] Austin ED Ma L LeDuc C Berman Rosenzweig E Borczuk A Phillips JA III Palomero T Sumazin P Kim HR Talati MH Whole exome sequencing to identify a novel gene (caveolin-1) associated with human pulmonary arterial hypertension. Circ Cardiovasc Genet. 2012;5:336–343. doi: 10.1161/CIRCGENETICS.111.961888 22474227 10.1161/CIRCGENETICS.111.961888 PMC3380156

[8] Ma L Roman-Campos D Austin ED Eyries M Sampson KS Soubrier F Germain M Trégouët DA Borczuk A Rosenzweig EB A novel channelopathy in pulmonary arterial hypertension. N Engl J Med. 2013;369:351–361. doi: 10.1056/NEJMoa1211097 23883380 10.1056/NEJMoa1211097 PMC3792227

[9] Gräf S Haimel M Bleda M Hadinnapola C Southgate L Li W Hodgson J Liu B Salmon RM Southwood M Identification of rare sequence variation underlying heritable pulmonary arterial hypertension. Nat Commun. 2018;9:1416 doi: 10.1038/s41467-018-03672-4 29650961 10.1038/s41467-018-03672-4 PMC5897357

[10] Turro E Astle WJ Megy K Gräf S Greene D Shamardina O Allen HL Sanchis-Juan A Frontini M Thys C; NIHR BioResource for the 100,000 Genomes Project. Whole-genome sequencing of patients with rare diseases in a national health system. Nature. 2020;583:96–102. doi: 10.1038/s41586-020-2434-2 32581362 10.1038/s41586-020-2434-2 PMC7610553

[11] Greene D Richardson S Turro E; NIHR BioResource. A fast association test for identifying pathogenic variants involved in rare diseases. Am J Hum Genet. 2017;101:104–114. doi: 10.1016/j.ajhg.2017.05.015 28669401 10.1016/j.ajhg.2017.05.015 PMC5501869

[12] Zhu N Pauciulo MW Welch CL Lutz KA Coleman AW Gonzaga-Jauregui C Wang J Grimes JM Martin LJ He H; PAH Biobank Enrolling Centers’ Investigators. Novel risk genes and mechanisms implicated by exome sequencing of 2572 individuals with pulmonary arterial hypertension. Genome Med. 2019;11:69 doi: 10.1186/s13073-019-0685-z 31727138 10.1186/s13073-019-0685-z PMC6857288

[13] Richards S Aziz N Bale S Bick D Das S Gastier-Foster J Grody WW Hegde M Lyon E Spector E; ACMG Laboratory Quality Assurance Committee. Standards and guidelines for the interpretation of sequence variants: a joint consensus recommendation of the American College of Medical Genetics and Genomics and the Association for Molecular Pathology. Genet Med. 2015;17:405–424. doi: 10.1038/gim.2015.30 25741868 10.1038/gim.2015.30 PMC4544753

[14] Zhu N Welch CL Wang J Allen PM Gonzaga-Jauregui C Ma L King AK Krishnan U Rosenzweig EB Ivy DD Rare variants in SOX17 are associated with pulmonary arterial hypertension with congenital heart disease. Genome Med. 2018;10:56 doi: 10.1186/s13073-018-0566-x 30029678 10.1186/s13073-018-0566-x PMC6054746

[15] FitzGerald G Botstein D Califf R Collins R Peters K Van Bruggen N Rader D The future of humans as model organisms. Science. 2018;361:552–553. doi: 10.1126/science.aau7779 30093589 10.1126/science.aau7779

[16] Karczewski KJ Francioli LC Tiao G Cummings BB Alföldi J Wang Q Collins RL Laricchia KM Ganna A Birnbaum DP; Genome Aggregation Database Consortium. The mutational constraint spectrum quantified from variation in 141,456 humans. Nature. 2020;581:434–443. doi: 10.1038/s41586-020-2308-7 32461654 10.1038/s41586-020-2308-7 PMC7334197

[17] Eyries M Montani D Girerd B Favrolt N Riou M Faivre L Manaud G Perros F Gräf S Morrell NW Familial pulmonary arterial hypertension by KDR heterozygous loss of function Eur Respir J. 2020;55:1902165. 31980491 10.1183/13993003.02165-2019

[18] Terman BI Carrion ME Kovacs E Rasmussen BA Eddy RL Shows TB Identification of a new endothelial cell growth factor receptor tyrosine kinase. Oncogene. 1991;6:1677–1683. 1656371

[19] Ferrara N Carver-Moore K Chen H Dowd M Lu L O’Shea KS Powell-Braxton L Hillan KJ Moore MW Heterozygous embryonic lethality induced by targeted inactivation of the VEGF gene. Nature. 1996;380:439–442. doi: 10.1038/380439a0 8602242 10.1038/380439a0

[20] Oladipupo SS Smith C Santeford A Park C Sene A Wiley LA Osei-Owusu P Hsu J Zapata N Liu F Endothelial cell FGF signaling is required for injury response but not for vascular homeostasis. Proc Natl Acad Sci U S A. 2014;111:13379–13384. doi: 10.1073/pnas.1324235111 25139991 10.1073/pnas.1324235111 PMC4169958

[21] Tuder RM Flook BE Voelkel NF Increased gene expression for VEGF and the VEGF receptors KDR/Flk and Flt in lungs exposed to acute or to chronic hypoxia. Modulation of gene expression by nitric oxide. J Clin Invest. 1995;95:1798–1807. doi: 10.1172/JCI117858 7706486 10.1172/JCI117858 PMC295709

[22] Cho YJ Han JY Lee SG Jeon BT Choi WS Hwang YS Roh GS Lee JD Temporal changes of angiopoietins and Tie2 expression in rat lungs after monocrotaline-induced pulmonary hypertension. Comp Med. 2009;59:350–356. 19712575 PMC2779210

[23] Tuder RM Chacon M Alger L Wang J Taraseviciene-Stewart L Kasahara Y Cool CD Bishop AE Geraci M Semenza GL Expression of angiogenesis-related molecules in plexiform lesions in severe pulmonary hypertension: evidence for a process of disordered angiogenesis. J Pathol. 2001;195:367–374. doi: 10.1002/path.953 11673836 10.1002/path.953

[24] Taraseviciene-Stewart L Kasahara Y Alger L Hirth P Mc Mahon G Waltenberger J Voelkel NF Tuder RM Inhibition of the VEGF receptor 2 combined with chronic hypoxia causes cell death-dependent pulmonary endothelial cell proliferation and severe pulmonary hypertension. FASEB J. 2001;15:427–438. doi: 10.1096/fj.00-0343com 11156958 10.1096/fj.00-0343com

[25] Itokawa T Nokihara H Nishioka Y Sone S Iwamoto Y Yamada Y Cherrington J McMahon G Shibuya M Kuwano M Antiangiogenic effect by SU5416 is partly attributable to inhibition of Flt-1 receptor signaling. Mol Cancer Ther. 2002;1:295–302. 12489845

[26] Fong TA Shawver LK Sun L Tang C App H Powell TJ Kim YH Schreck R Wang X Risau W SU5416 is a potent and selective inhibitor of the vascular endothelial growth factor receptor (Flk-1/KDR) that inhibits tyrosine kinase catalysis, tumor vascularization, and growth of multiple tumor types. Cancer Res. 1999;59:99–106. 9892193

[27] Kasahara Y Tuder RM Taraseviciene-Stewart L Le Cras TD Abman S Hirth PK Waltenberger J Voelkel NF Inhibition of VEGF receptors causes lung cell apoptosis and emphysema. J Clin Invest. 2000;106:1311–1319. doi: 10.1172/JCI10259 11104784 10.1172/JCI10259 PMC387249

[28] Garcia AA Hirte H Fleming G Yang D Tsao-Wei DD Roman L Groshen S Swenson S Markland F Gandara D Phase II clinical trial of bevacizumab and low-dose metronomic oral cyclophosphamide in recurrent ovarian cancer: a trial of the California, Chicago, and Princess Margaret Hospital phase II consortia. J Clin Oncol. 2008;26:76–82. doi: 10.1200/JCO.2007.12.1939 18165643 10.1200/JCO.2007.12.1939

[29] Montani D Bergot E Günther S Savale L Bergeron A Bourdin A Bouvaist H Canuet M Pison C Macro M Pulmonary arterial hypertension in patients treated by dasatinib. Circulation. 2012;125:2128–2137. doi: 10.1161/CIRCULATIONAHA.111.079921 22451584 10.1161/CIRCULATIONAHA.111.079921

[30] El-Dabh A Acharya D EXPRESS: pulmonary hypertension with dasatinib and other tyrosine kinase inhibitors Pulm Circ. 2019;9:2045894019865704. 10.1177/2045894019865704 PMC6664660 31274047

[31] Bleyl S Nelson L Odelberg SJ Ruttenberg HD Otterud B Leppert M Ward K A gene for familial total anomalous pulmonary venous return maps to chromosome 4p13-q12. Am J Hum Genet. 1995;56:408–415. 7847375 PMC1801122

[32] Reuter MS Jobling R Chaturvedi RR Manshaei R Costain G Heung T Curtis M Hosseini SM Liston E Lowther C Haploinsufficiency of vascular endothelial growth factor related signaling genes is associated with tetralogy of Fallot. Genet Med. 2019;21:1001–1007. doi: 10.1038/s41436-018-0260-9 30232381 10.1038/s41436-018-0260-9 PMC6752294

[33] Test VJ Farber HW McGoon MD Parsons L Channick RN Pulmonary arterial hypertension in the elderly: baseline characteristics and evaluation of therapeutics. An examination of the reveal registry Am J Respir Crit Care Med. 2020;201:A2649.

[34] Hadinnapola C Bleda M Haimel M Screaton N Swift A Dorfmüller P Preston SD Southwood M Hernandez-Sanchez J Martin J; NIHR BioResource–Rare Diseases Consortium; UK National Cohort Study of Idiopathic and Heritable PAH. Phenotypic characterization of EIF2AK4 mutation carriers in a large cohort of patients diagnosed clinically with pulmonary arterial hypertension. Circulation. 2017;136:2022–2033. doi: 10.1161/CIRCULATIONAHA.117.028351 28972005 10.1161/CIRCULATIONAHA.117.028351 PMC5700414

[35] Trip P Girerd B Bogaard HJ de Man FS Boonstra A Garcia G Humbert M Montani D Vonk-Noordegraaf A Diffusion capacity and BMPR2 mutations in pulmonary arterial hypertension. Eur Respir J. 2014;43:1195–1198. doi: 10.1183/09031936.00136413 24036241 10.1183/09031936.00136413

[36] Nathan SD Barbera JA Gaine SP Harari S Martinez FJ Olschewski H Olsson KM Peacock AJ Pepke-Zaba J Provencher S Pulmonary hypertension in chronic lung disease and hypoxia Eur Respir J. 2019;53:1801914. 30545980 10.1183/13993003.01914-2018 PMC6351338

[37] Montani D Girerd B Jaïs X Levy M Amar D Savale L Dorfmüller P Seferian A Lau EM Eyries M Clinical phenotypes and outcomes of heritable and sporadic pulmonary veno-occlusive disease: a population-based study. Lancet Respir Med. 2017;5:125–134. 28087362 10.1016/S2213-2600(16)30438-6

[38] Montani D Lau EM Dorfmüller P Girerd B Jaïs X Savale L Perros F Nossent E Garcia G Parent F Pulmonary veno-occlusive disease. Eur Respir J. 2016;47:1518–1534. 27009171 10.1183/13993003.00026-2016

