# Supplementary material for: Bayesian Inference Associates Rare KDR Variants With Specific Phenotypes in Pulmonary Arterial Hypertension
Source: Circ Genom Precis Med. 2020 Dec 15;14(1):e003155. doi: 10.1161/CIRCGEN.120.003155 (PMC7892262; doi:10.1161/CIRCGEN.120.003155)
Supplement: Supplementary file 1 [file hcg-14-e003155-s001.pdf]

SUPPLEMENTAL MATERIAL

Methods

Cohort description

Carriers of Val122Ile (NM_000371.3:c.424G>A) risk allele were selected from the Yale-Penn cohort,[1–4] whose medical history was obtained as part of the phenotyping effort, as previously reported[5]. In accordance with the International Society of Amyloidosis nomenclature committee [6] amyloid fibril protein variants are named according to the substitution or deletion in the mature amyloid protein (e.g., TTR V30M). The

We investigated two binary phenotypes – self-reported history of heart disease ("*Has a doctor ever told you that you have (had) a heart disease?*") and having had 10 or more outpatient surgeries. We previously reported that these two phenotypes were associated with the Val122Ile mutation in the Yale-Penn cohort[5]. We assayed 104 African Americans, samples from 8 of which failed the assay, leaving 96 carriers for analysis (mean age = 42.16 ± 9.9 [SD] yrs.). Epigenomic differences were tested with respect to the history of heart disease (N=90 controls; 6 cases; Males-47%) and 10 or more outpatient surgeries (N=94 controls, 2 cases; Males-46%).

The significant methylation sites were investigated in an independent cohort of Italian participants of European descent. This study included 48 carriers of *TTR* mutations (Val30Met, Phe64Leu, Ile68Leu, Ala120Ser, and Val122Ile) with the diagnosis of TTR amyloidosis confirmed via positive amyloid fibril deposition in addition to clinical symptoms[7]. Thirty-two healthy individuals with none of the *TTR* mutations or clinical symptoms from the same local area were recruited as controls. Detailed information regarding this cohort was previously reported[8,9]. The methylation array analysis was performed at the Connecting Bio-research and Industry Center, Trieste, Italy, using the same sample protocol implemented in the Yale-Penn cohort.

Sample and array processing

DNA was extracted from whole blood of Yale-Penn participants using the EZ-96 DNA methylation kit (Zymo Research, CA, USA). As previously described[4], the samples were genotyped at the Yale Center for Genome Analysis (YCGA), the Center for Inherited Disease Research, and the Gelernter laboratory at Yale (VA CT) using genome-wide arrays (Illumina HumanOmni1-Quad v1.0 and Illumina HumanCoreExome arrays), the imputation was performed with IMPUTE2.0, and principal components were derived on the QC'd genomic data . The Val122Ile – rs76992529 probe was genotyped in the HumanCoreExome array and imputed in the HumanOmni1-Quad v1.0 array with high imputation quality (INFO score = 0.98) to determine TTR carriers in the African-American Yale-Penn participants[5]. DNA methylation was assayed using the Illumina Infinium MethylationEPIC chip quantifying >850,000 CpG sites and imaged on the Illumina iScan system at YCGA. The methylation intensity data (*.idat files) was exported for analysis using the manufacturer's recommended protocol using the GenomeStudio methylation module.

DNA methylation analysis

All analyses were performed in R 3.6. The methylation intensity files (*.idat) were imported into *ChAMP*[10] for post-processing and normalization. The beta values, ranging from 0 to 1 were generated for all CpG sites representing the ratio of methylated to unmethylated fluorescent intensities. Primary QC removed CpG sites with low detection p-value, missing beads, sites near SNPs, multi-hit and non-autosomal sites. The beta values of the remaining 737,385 sites were normalized using the beta mixture quantile (BMIQ) method, followed by analysis of batch effects using singular vector decomposition. Technical batch effects for array and slide were corrected using ComBat. The blood cell-type composition and smoking status[11] was derived from methylation data. The association analysis for CpG sites was performed on M-values (transformed beta values) using empirical Bayes methods implemented in the *limma* package. The association was

adjusted for age, sex, tobacco use, smoking, genotype-derived principal components 1-10, and blood cell type proportions: CD8+T cells, CD4+ T cells, natural killer cells, B-cells, monocytes, and neutrophils. Genomic inflation was calculated using the *QQperm* package; both phenotypic associations had a lambda of 1 or less (Heart Disease – 1.01 and Outpatient Surgery – 0.92) indicating lack of population substructure bias. Due to the unbalanced case-control proportions, we also performed permutation of the association using the *CpGassoc* package[12]. Differential methylation of regions was calculated using *DMRcate*. The sites and regions were deemed significant considering a false discovery rate of <0.05 ($FDR_{p\text{-value}}<0.05$). Differentially expressed PPI network modules were investigated using the *FEM* package[13]. Gene ontology using significant genes mapped from significant CpG sites and regions was assessed with *ShinyGO* [14]. The cis-methylation QTL (association between SNPs and significant methylated CpG sites) was performed with all the aforementioned covariates using *MatrixQTL*[15], wherein the local distance was defined using the biologically expected and default value of 1 Mb between SNP and CpG sites. Significant associations were identified using genomic control adjustment (GC) p-values < 0.05, in addition to $FDR_{p\text{-value}} < 0.05$. Epigenetic age – defined as the cumulative score of specific CpG sites across the genome -- was calculated using Hannum and Horvath's clock from the *wateRmelon* package[16]. As suggested by the developers of each of the epigenetic clocks, biological age, and chronological age were significantly correlated, R≥0.90; p<0.001. The difference in the epigenetic (biological) age between cases and controls was tested using Student's t-test. The network connecting all of the significant genes detected from the above analyses was constructed using GeneMANIA[17]. The results were visualized using ggplot2, coMET[18], and FUMA[19].

Medical history of TTR Val122Ile carriers

	Heart Disease (N)		10 or more outpatient surgeries (N)	
	Yes |No	p-value	Yes|No	p-value
Medical history				
**Migraine**				
**No**	6|80	1.00	2|84	1.00
**Yes**	0|10		0|10	
**Brain Injury/ concussion**				
**No**	6|85	1.00	2|89	1.00
**Yes**	0|5		0|5	
**Been unconscious for longer than 5 min**				
**No**	4|82	0.11	2|84	1.00
**Yes**	2|8		0|10	
**Epilepsy/Seizure**				
**No**	6|85	1.00	2|89	1.00
**Yes**	0|5		0|5	
**Meningitis/ encephalitis**				
**No**	6|90	N.A	2|90	N.A.
**Yes**	0|0		0|0	
**Stroke**				
**No**	6|89	1.00	2|93	1.00
**Yes**	0|1		0|1	
**Liver disease**				
**No**	6|83		2|87	1.00
**Yes**	0|7		0|7	
**Thyroid disease**				
**No**	5|86	0.28	2|89	1.00
**Yes**	1|4		0|5	
**Asthma**				
**No**	4|74	0.31	2|76	1.00
**Yes**	2|16		0|18	
**Diabetes**				
**No**	4|81	0.13	2|83	1.00
**Yes**	2|9		0|11	
**Cancer**				
**No**	5|88	0.17	2|91	1.00
**Yes**	1|22		0|3	
**HIV**				
**No**	6|86	1	2|90	1.00
**Yes**	0|4		0|4	

References

1. Polimanti R, Meda SA, Pearlson GD, Zhao H, Sherva R, Farrer LA, Kranzler HR, Gelernter J. S100A10 identified in a genome-wide gene × cannabis dependence interaction analysis of risky sexual behaviours. *Journal of Psychiatry & Neuroscience*. 2017;42(4):252–261.

2. Polimanti R, Kaufman J, Zhao H, Kranzler HR, Ursano RJ, Kessler RC, Gelernter J, Stein MB. A genome-wide gene-by-trauma interaction study of alcohol misuse in two independent cohorts identifies PRKG1 as a risk locus. *Molecular Psychiatry*. 2018;23(1):154–160.

3. Polimanti R, Wang Q, Meda SA, Patel KT, Pearlson GD, Zhao H, Farrer LA, Kranzler HR, Gelernter J. The Interplay Between Risky Sexual Behaviors and Alcohol Dependence: Genome-Wide Association and Neuroimaging Support for LHPP as a Risk Gene. *Neuropsychopharmacology*. 2017;42(3):598–605.

4. Polimanti R, Zhang H, Smith AH, Zhao H, Farrer LA, Kranzler HR, Gelernter J. Genome-wide association study of body mass index in subjects with alcohol dependence. *Addiction Biology*. 2017;22(2):535–549.

5. Polimanti R, Nuñez YZ, Gelernter J. Increased Risk of Multiple Outpatient Surgeries in African-American Carriers of Transthyretin Val122Ile Mutation Is Modulated by Non-Coding Variants. *Journal of clinical medicine*. 2019;8(2).

6. Benson MD, Buxbaum JN, Eisenberg DS, Merlini G, Saraiva MJM, Sekijima Y, Sipe JD, Westermark P. Amyloid nomenclature 2018: recommendations by the International Society of Amyloidosis (ISA) nomenclature committee. *Amyloid*. 2018;25(4):215–219.

7. De Lillo A, Pathak GA, De Angelis F, Di Girolamo M, Luigetti M, Sabatelli M, Perfetto F, Frusconi S, Manfellotto D, Fuciarelli M, Polimanti R. Epigenetic profiling of Italian patients identified methylation sites associated with hereditary transthyretin amyloidosis. *Clinical epigenetics*. 2020;12(1):176.

8. Iorio A, De Angelis F, Di Girolamo M, Luigetti M, Pradotto L, Mauro A, Manfellotto D, Fuciarelli M, Polimanti R. Most recent common ancestor of TTR Val30Met mutation in Italian population and its potential role in genotype-phenotype correlation. *Amyloid*. 2015;22(2):73–78.

9. Iorio A, De Lillo A, De Angelis F, Di Girolamo M, Luigetti M, Sabatelli M, Pradotto L, Mauro A, Mazzeo A, Stancanelli C, Perfetto F, Frusconi S, My F, Manfellotto D, Fuciarelli M, et al. Non-coding variants contribute to the clinical heterogeneity of TTR amyloidosis. *European Journal of Human Genetics*. 2017;25(9):1055–1060.

10. Tian Y, Morris TJ, Webster AP, Yang Z, Beck S, Feber A, Teschendorff AE. ChAMP: updated methylation analysis pipeline for Illumina BeadChips. *Bioinformatics*. 2017;33(24):3982–3984.

11. McCartney DL, Stevenson AJ, Hillary RF, Walker RM, Bermingham ML, Morris SW, Clarke T-K, Campbell A, Murray AD, Whalley HC, Porteous DJ, Visscher PM, McIntosh AM, Evans KL, Deary IJ, et al. Epigenetic signatures of starting and stopping smoking. *EBioMedicine*. 2018;37:214–220.

12. Barfield RT, Kilaru V, Smith AK, Conneely KN. CpGassoc: an R function for analysis of DNA methylation microarray data. *Bioinformatics*. 2012;28(9):1280–1281.

13. Jiao Y, Widschwendter M, Teschendorff AE. A systems-level integrative framework for genome-wide DNA methylation and gene expression data identifies differential gene expression modules under epigenetic control. *Bioinformatics*. 2014;30(16):2360–2366.

14. Ge SX, Jung D, Yao R. ShinyGO: a graphical gene-set enrichment tool for animals and plants. *Bioinformatics*. 2020;36(8):2628–2629.

15. Shabalin AA. Matrix eQTL: ultra fast eQTL analysis via large matrix operations. *Bioinformatics*. 2012;28(10):1353–1358.

16. Pidsley R, Y Wong CC, Volta M, Lunnon K, Mill J, Schalkwyk LC. A data-driven approach to preprocessing Illumina 450K methylation array data. *BMC Genomics*. 2013;14:293.

17. Montojo J, Zuberi K, Rodriguez H, Bader GD, Morris Q. GeneMANIA: Fast gene network construction and function prediction for Cytoscape. [version 1; peer review: 2 approved]. *F1000Research*. 2014;3:153.

18. Martin TC, Yet I, Tsai P-C, Bell JT. coMET: visualisation of regional epigenome-wide association scan results and DNA co-methylation patterns. *BMC Bioinformatics*. 2015;16:131.

19. Watanabe K, Taskesen E, van Bochoven A, Posthuma D. Functional mapping and annotation of genetic associations with FUMA. *Nature Communications*. 2017;8(1):1826.

Table of Contents

Table S1: Differentially methylation sites 2

A. HEART DISEASE 2

B. OUTPATIENT SURGERIES ≥ 10 3

Table S2: Differentially methylated regions 4

Table S3: Over representated gene ontology for genes identified via differentially methylated sites and regions........ 5

Table S4: Functional PPI modules 5

A. HEART DISEASE 5

B. OUTPATIENT SURGERIES ≥ 10 7

Table S5: Local quantitative trait loci (SNPs) for methylated sites (mQTL) 7

Table S6: Epigenomic age 8

Table S7: Δ DNAm age (difference in epigenetic and chronological age) 8

Table S1: Differentially methylation sites

A. HEART DISEASE

chr	pos	strand	Name	Relation to Island	UCSC RefGene Group	HMM Island	GencodeBasicV12 NAME	DNase Hypersensitivity NAME	DNase Hypersensitivity Evidence Count	OpenChromatin NAME	OpenChromatin Evidence Count	logFC	AveExpr	t	P.Value	adj.P.Val	B	Permutation P-value	Permutation FDR P-value
**9**	130286880	-	cg06641417	N_Shelf	Body;Body		FAM129B	chr9:130286700-130287035	3			-1.822	3.671	-6.572	7.35E-09	0.0031	6.037	1.62027E-08	0.0053
**1**	2213966	+	cg26033908	S_Shore	Body	1:2201881-2203883	SKI			chr1:2213946-2216731	6	-1.615	4.348	-6.537	8.51E-09	0.0031	5.949	1.7385E-08	0.0053
**6**	170102056	+	cg14890866	Island	1stExon;1stExon;TSS200;5'UTR;5'UTR	6:169843601-169844984	WDR27;WDR27;WDR27;C6orf120;C6orf120;WDR27;WDR27			chr6:170101364-170103125	6	-2.028	-5.709	-6.434	1.30E-08	0.0032	5.693	2.96666E-08	0.0055
**2**	191745248	+	cg15522719	N_Shore	TSS1500	2:191453290-191455085	GLS;GLS			chr2:191744426-191748461	6	-1.731	-7.329	-6.301	2.25E-08	0.0042	5.361	4.6856E-08	0.0069
**8**	126391560	+	cg18546846	OpenSea						chr8:126390915-126391669	5	-0.786	3.859	-6.235	2.96E-08	0.0044	5.196	2.14499E-08	0.0053

B. OUTPATIENT SURGERIES ≥ 10

chr	pos	strand	Name	Relation to Island	UCSC RefGene Group	HMM Island	GencodeBasicV12 NAME	DNase Hypersensitivity NAME	DNase Hypersensitivity Evidence Count	OpenChromatin NAME	OpenChromatin Evidence Count	logFC	AveExpr	t	P.Value	adj.P.Val	B	Permutation P-value	Permutation FDR P-value
**22**	44756530	-	cg25814327	OpenSea						chr22:44756108-44757661	6	-2.075	4.465	-6.385	1.57E-08	0.012	-1.283	3.02092E-08	0.0223
**16**	5008289	-	cg03718655	OpenSea	TSS200	16:4947946-4948690	SEC14L5	chr16:5007725-5008690	3			-2.673	-4.698	-6.027	6.83E-08	0.015	-1.543	1.49374E-07	0.0332
**10**	127059333	-	cg05189127	OpenSea		10:127049298-127049395		chr10:127059185-127059435	3			1.885	0.619	5.986	0.000	8.09E-08	-1.574	1.41261E-07	0.0332
**2**	181846570	-	cg13998023	S Shore	5'UTR;5'UTR;5'UTR;5'UTR		UBE2E3;UBE2E3;UBE2E3;UBE2E3;UBE2E3;UBE2E3;UBE2E3	chr2:181846000-181846690	3			-2.632	-4.821	-5.978	8.35E-08	0.015	-1.579	1.80027E-07	0.0332

Table S2: Differentially methylated regions

A. HEART DISEASE

Chromosome	start	end	width	no.cpgs	min smoothed fdr	Overlapping Genes
**chr12**	4918335	4919081	747	6	1.08E-12	GALNT8, KCNA6

B. OUTPATIENT SURGERIES ≥ 10

Chromosome	start	end	width	no.cpgs	min smoothed fdr	Overlapping Genes
**chr10**	127059333	127059374	42	2	1.70E-08	N/A
**chr1**	36787932	36788627	696	5	1.30E-09	snoU13, Y RNA, SCARNA16, SCARNA21, U1, SCARNA17, SCARNA18, SCARNA24, SNORD112, SNORA62, SH3D21, SNORA63, SNORD46, SNORA2, SNORD81, U3, SNORA51, SNORA25, SCARNA20, SNORA67, U6, SNORA70, SNORA77, SNORA26, U8, SCARNA11, EVA1B, SNORA31, SNORA42, SNORA40, SNORD64, ACA64, snoU109, SNORD60
**chr14**	24780404	24780557	154	4	3.89E-07	LTB4R2, SNORA79, CIDEB

Table S3: Over representated gene ontology for genes identified via differentially methylated sites and regions

Enrichment FDR p-value	Genes in list	Total genes	Functional Category	Genes
0.00260277	4	395	Protein homooligomerization	GLS SKI GALNT8 KCNA6
0.00479721	2	39	Myotube cell development	FAM129B SKI
0.00479721	2	35	Skeletal muscle fiber development	FAM129B SKI
0.00479721	4	641	Protein complex oligomerization	GLS SKI GALNT8 KCNA6
0.01005811	2	63	Positive regulation of DNA binding	FAM129B SKI
0.01125224	2	73	Muscle fiber development	FAM129B SKI
0.02382091	2	115	Myotube differentiation	SKI FAM129B
0.02738267	2	132	Regulation of DNA binding	FAM129B SKI
0.03104622	2	152	Negative regulation of cellular response to growth factor stimulus	SKI FAM129B
0.03104622	2	171	Skeletal muscle tissue development	FAM129B SKI
0.03104622	2	178	Muscle cell development	FAM129B SKI
0.03104622	2	164	Striated muscle cell development	FAM129B SKI
0.03104622	2	180	Skeletal muscle organ development	FAM129B SKI
0.03172491	2	189	Positive regulation of binding	FAM129B SKI
0.04877586	2	244	Potassium ion transport	KCNA6 GALNT8

Table S4: Functional PPI modules

A. HEART DISEASE

Symbol (Seed)	Size	Mod	P	Genes
**ABCA1**	43	1.262	0.001	ABCA1 ARHGEF11 RAPGEF6 SNTA1 SNTB1 LIN7A LIN7B UTRN ARHGEF12 SNTB2 MPDZ UGP2 AOX1 SPTLC1 ADRA1D MAST1 LPAR1 SPTLC2 MAST2 LPAR2 PTPRN MRAS SNTG1 DTNB DTNA SNTG2 SSPN SYNC SYNM DTNBP1 BLOC1S2 BLOC1S4 BLOC1S1 PTPRT APBA1 CNTNAP4 SNX27 KIF17 MAPK12 ADRA1A NOS1 RASD1 VAC14

EntrezID	Symbol	stat(DNAm)	P(DNAm)	stat(Int)
**19**	ABCA1	-2.8	0.006	2.8
**6645**	SNTB2	2.52	0.013	2.52

**282991**	BLOC1S2	2.41	0.018	2.41
**64130**	LIN7B	2.22	0.029	2.22
**4842**	NOS1	-1.77	0.08	1.77
**1837**	DTNA	1.67	0.098	1.67
**7402**	UTRN	-1.66	0.101	1.66
**51735**	RAPGEF6	1.57	0.12	1.57
**6641**	SNTB1	1.55	0.123	1.55
**9826**	ARHGEF11	1.46	0.149	1.46
**146**	ADRA1D	-1.42	0.159	1.42
**9517**	SPTLC2	1.34	0.182	1.34
**84062**	DTNBP1	1.21	0.228	1.21
**10558**	SPTLC1	-1.17	0.244	1.17
**81609**	SNX27	-1.08	0.285	1.08
**85445**	CNTNAP4	1.01	0.314	1.01
**7360**	UGP2	1	0.318	1
**6300**	MAPK12	0.91	0.363	0.91
**8082**	SSPN	0.86	0.394	0.86
**1902**	LPAR1	0.8	0.428	0.8
**54212**	SNTG1	0.77	0.446	0.77
**6640**	SNTA1	0.7	0.487	0.7
**55330**	BLOC1S4	-0.58	0.561	0.58
**23139**	MAST2	-0.58	0.567	0.58
**22983**	MAST1	-0.57	0.57	0.57
**5798**	PTPRN	0.56	0.575	0.56
**54221**	SNTG2	0.53	0.594	0.53
**22808**	MRAS	-0.53	0.596	0.53
**81493**	SYNC	-0.53	0.598	0.53
**51655**	RASD1	0.5	0.619	0.5
**23365**	ARHGEF12	0.48	0.629	0.48
**57576**	KIF17	-0.45	0.654	0.45
**320**	APBA1	-0.41	0.686	0.41
**148**	ADRA1A	0.3	0.767	0.3
**8825**	LIN7A	0.25	0.807	0.25
**2647**	BLOC1S1	-0.19	0.85	0.19
**55697**	VAC14	0.17	0.861	0.17
**316**	AOX1	-0.16	0.87	0.16
**8777**	MPDZ	-0.14	0.886	0.14
**11122**	PTPRT	-0.09	0.926	0.09
**1838**	DTNB	-0.05	0.958	0.05
**9170**	LPAR2	0.04	0.972	0.04
**23336**	SYNM	0.03	0.977	0.03

- Highlighted rows in green show significant value of genes that serve as targets in the network.

B. OUTPATIENT SURGERIES ≥ 10

Symbol(Seed)	Size	Mod	P	Genes
**EXOSC4**	17	1.299737887	0.005	EXOSC4 AICDA EXOSC2 DCP2 MPHOSPH6 EXOSC9 EXOSC3 ZNF598 NEK1 AKR1A1 KHSRP A1CF KNSTRN ARHGAP18 ZNF350 CTNNAL1 THOP1

EntrezID	Symbol	stat(DNAm)	P(DNAm)	stat(Int)
**54512**	EXOSC4	-3.14	0.002	3.14
**90850**	ZNF598	-1.75	0.084	1.75
**51010**	EXOSC3	-1.7	0.092	1.7
**23404**	EXOSC2	-1.22	0.224	1.22
**8570**	KHSRP	-1.07	0.286	1.07
**10327**	AKR1A1	-0.87	0.387	0.87
**5393**	EXOSC9	-0.83	0.411	0.83
**4750**	NEK1	-0.79	0.429	0.79
**10200**	MPHOSPH6	-0.61	0.543	0.61
**8727**	CTNNAL1	-0.61	0.546	0.61
**167227**	DCP2	-0.48	0.63	0.48
**90417**	KNSTRN	0.43	0.667	0.43
**7064**	THOP1	-0.38	0.702	0.38
**29974**	A1CF	0.36	0.718	0.36
**93663**	ARHGAP18	0.36	0.722	0.36
**59348**	ZNF350	-0.23	0.818	0.23
**57379**	AICDA	0.07	0.94	0.07

Table S5: Local quantitative trait loci (SNPs) for methylated sites (mQTL)

Chr	Position	rsID	mSite	Statistic	P-value	FDR	P-value.gc
9	9:130148962	rs192528579	cg06641417	-16.0471	4.09E-24	5.50E-20	0.04
9	9:130223401	rs182192023	cg06641417	-16.0471	4.09E-24	5.50E-20	0.04
9	9:130225162	rs114553373	cg06641417	-16.0471	4.09E-24	5.50E-20	0.04
9	9:130225427	rs187644239	cg06641417	-16.0471	4.09E-24	5.50E-20	0.04
9	9:130264053	rs114896522	cg06641417	-16.0471	4.09E-24	5.50E-20	0.04
9	9:130286881	rs139996037	cg06641417	-16.0471	4.09E-24	5.50E-20	0.04
9	9:129748363	rs143219617	cg06641417	-9.90637	1.53E-14	1.12E-10	0.20
9	9:129995636	rs115397220	cg06641417	-9.7456	2.88E-14	2.03E-10	0.21
9	9:130013802	rs140087528	cg06641417	-9.7456	2.88E-14	2.03E-10	0.21

Table S6: Epigenomic age

Biological Age in Years (mean±SD)		95% CI	P-value
Horvath Epigenetic Clock for Heart Disease			
Cases	56.6 ± 12.9	3.8, 23.4	0.007055
Control	43 ± 11.7		
Hannum Epigenetic Clock for Heart Disease			
Cases	45.7± 11	5.6, 20.9	0.0009115
Controls	32.5 ± 9.07		

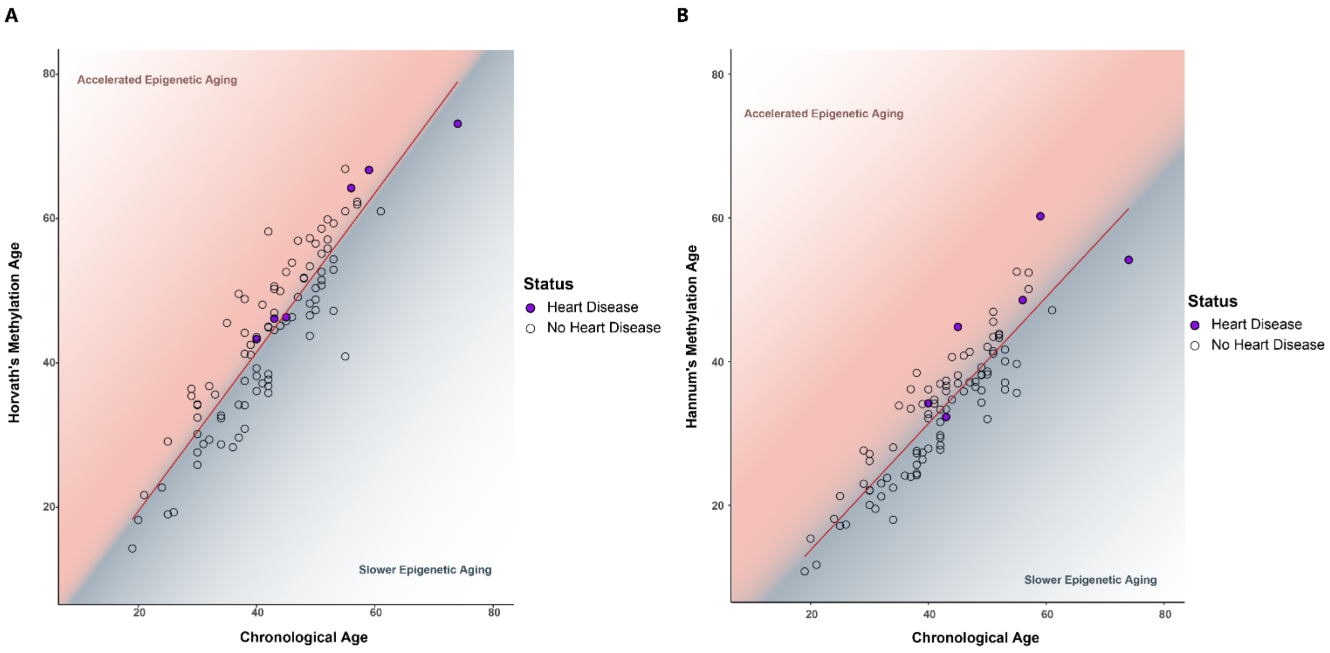

Table S7: Δ DNAm age (difference in epigenetic and chronological age)

Δ DNAm Age in Years (mean±SD)		95% CI	P-value
ΔHorvath Epigenetic Clock for Heart Disease			
Cases	3.81 ± 3.56	-2.15, 6.65	0.31
Control	1.57 ± 5.34		
ΔHannum Epigenetic Clock for Heart Disease			
Cases	-7.12± 7.68	-6.15, 9.92	0.57
Controls	-9.00 ± 4.02		

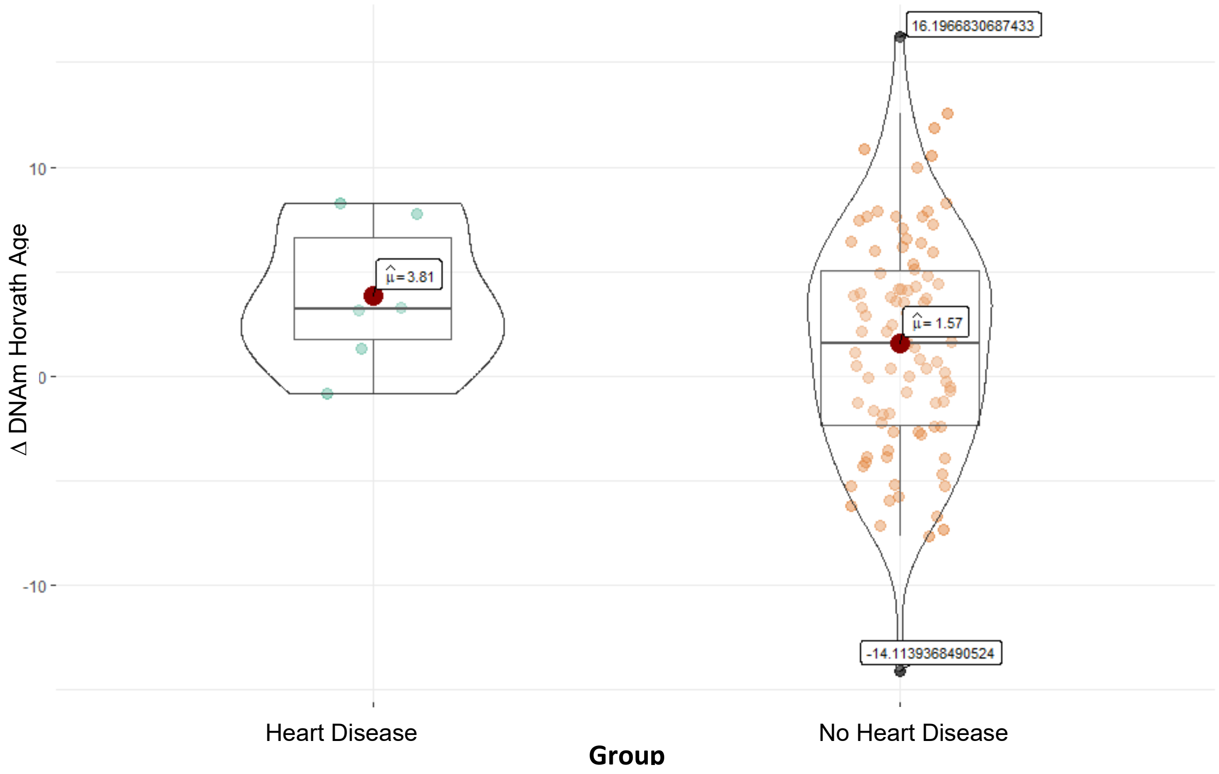
16.1966830687433
10
0
-10
Δ DNAm Horvath Age
μ̂=3.81
μ̂= 1.57
-14.1139368490524
Heart Disease
No Heart Disease
Group

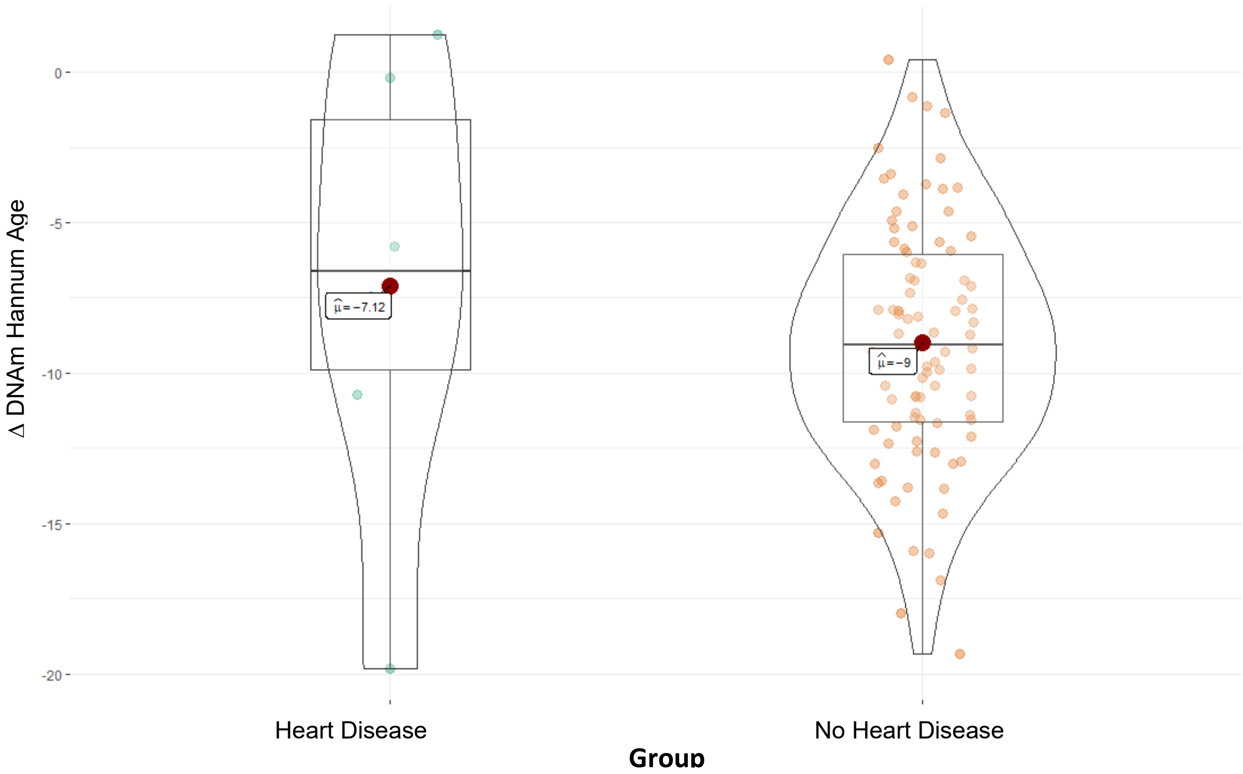
0
-5
-10
-15
-20
Δ DNAm Hannum Age
μ̂=−7.12
μ̂=−9
Heart Disease
No Heart Disease
Group

GeneMANIA report

Created on : 19 February 2020 09:25:42
Last database update : 13 March 2017 00:00:00
Application version : 3.6.0

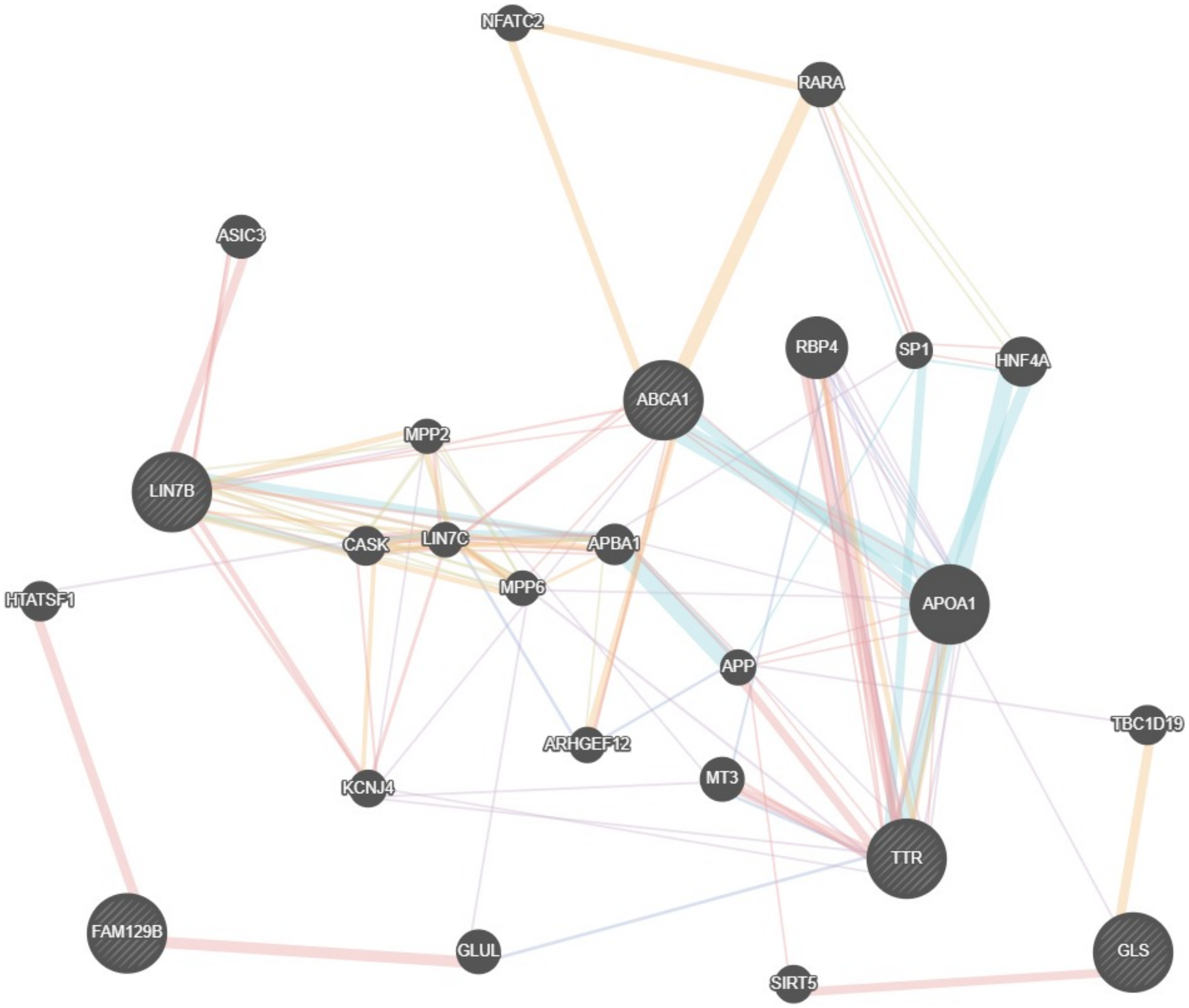

Networks

- Physical Interactions
- Co-expression
- Predicted
- Co-localization
- Pathway
- Genetic Interactions
- Shared protein domains

Functions

N/A

Search parameters

Organism Homo sapiens (human)

Genes LIN7B , GLS , TTR , FAM129B , ABCA1

Network weighting Automatically selected weighting method

Networks

A

Abu-Odeh-Aqeilan-2014 , Agrawal-Sedivy-2010 , Aichem-Groettrup-2012 , Albers-Koegl-2005 , Alexandru-Deshaies-2008 , Alizadeh-Staudt-2000 , Andresen-Flores-Morales-2014 , Arbuckle-Grant-2010 , Arroyo-Aloy-2014 , Arroyo-Aloy-2015

B

Bahr-Bowler-2013 , Bailey-Hieter-2015 , Bandyopadhyay-Ideker-2010 , Bantscheff-Drewes-2011 , Barr-Knapp-2009 , Barrios-Rodiles-Wrana-2005 , Behrends-Harper-2010 , Behzadnia-Lührmann-2007 , Bennett-Harper-2010 , Benzinger-Hermeking-2005 , Berggård-James-2006 , Bett-Hay-2013 , Bhatnagar-Attie-2014 , Bild-Nevins-2006 B , BIOGRID-SMALL-SCALE-STUDIES , BIOGRID-SMALL-SCALE-STUDIES , Blandin-Richard-2013 , Blomen-Brummelkamp-2015 , Blomen-Brummelkamp-2015 , Bogachek-Weigel-2014 , Boldrick-Relman-2002 , Bonacci-Soubeyran-2014 , Bouwmeester-Superti-Furga-2004 , Brajenovic-Drewes-2004 , Brehme-Superti-Furga-2009 , Bruderer-Hay-2011 , Burington-Shaughnessy-2008 , Butland-Hayden-2014 , Byron-Humphries-2012

C

Cai-Conaway-2007 , Camargo-Brandon-2007 , Campos-Reinberg-2015 , Cao-Chinnaiyan-2014 , Carmon-Liu-2014 , CELL_MAP , Chen-Brown-2002 , Chen-Ge-2013 , Chen-Huang-2014 , Chen-Zhang-2013 , Christianson-Kopito-2011 , Cloutier-Coulombe-2013 , Colland-Gauthier-2004 , Corominas-Iakoucheva-2014 , Couzens-Gingras-2013 , Cox-Rizzino-2013 , Coyaud-Raught-2015

D

Danielsen-Nielsen-2011 , Dart-Wells-2015 , de Hoog-Mann-2004 , Diner-Cristea-2015 , Dobbin-Giordano-2005 , Drissi-Boisvert-2015 , Dyer-Sobral-2010

E

Emanuele-Elledge-2011 , Emdal-Olsen-2015 , Ewing-Figeys-2007

F

Fenner-Prehn-2010 , Floyd-Pagliarini-2016 , Foerster-Ritter-2013 , Fogeron-Lange-2013 , Foster-Marshall-2013 , Freibaum-Taylor-2010

G

Gabriel-Baumgrass-2016 , Galligan-Howley-2015 , Gao-Reinberg-2012 , Gautier-Hall-2009 , Giannone-Liu-2010 , Glatter-Gstaiger-2009 , Gloeckner-Ueffing-2007 ,

G

Goehler-Wanker-2004 , Golebiowski-Hay-2009 , Goudreault-Gingras-2009 , Grant-2010 , Greco-Cristea-2011 , Grossmann-Stelzl-2015 , Guarani-Harper-2014 , Gupta-Pelletier-2015

H

Hanson-Clayton-2014 , Hauri-Gstaiger-2013 , Havrylov-Redowicz-2009 , Havugimana-Emili-2012 , Hayes-Urbé-2012 , Hegele-Stelzl-2012 A , Hegele-Stelzl-2012 B , Hein-Mann-2015 , Hill-Livingston-2014 , HUMANCYC , Humphries-Humphries-2009 , Hutchins-Peters-2010 , Huttlin-Gygi-2015

I

I2D-BIND-Fly2Human , I2D-BIND-Mouse2Human , I2D-BIND-Rat2Human , I2D-BIND-Worm2Human , I2D-BIND-Yeast2Human , I2D-BioGRID-Fly2Human , I2D-BioGRID-Mouse2Human , I2D-BioGRID-Rat2Human , I2D-BioGRID-Worm2Human , I2D-BioGRID-Yeast2Human , I2D-Chen-Pawson-2009-PiwiScreen-Mouse2Human , I2D-Formstecher-Daviet-2005-Embryo-Fly2Human , I2D-Giot-Rothbert-2003-Low-Fly2Human , I2D-INNATEDB-Mouse2Human , I2D-IntAct-Fly2Human , I2D-IntAct-Mouse2Human , I2D-IntAct-Rat2Human , I2D-IntAct-Worm2Human , I2D-IntAct-Yeast2Human , I2D-Krogan-Greenblatt-2006-Core-Yeast2Human , I2D-Krogan-Greenblatt-2006-NonCore-Yeast2Human , I2D-Li-Vidal-2004-CORE-1-Worm2Human , I2D-Li-Vidal-2004-non-core-Worm2Human , I2D-Manual-Mouse2Human , I2D-Manual-Rat2Human , I2D-MGI-Mouse2Human , I2D-MINT-Fly2Human , I2D-MINT-Mouse2Human , I2D-MINT-Rat2Human , I2D-MINT-Worm2Human , I2D-MINT-Yeast2Human , I2D-Ptacek-Snyder-2005-Yeast2Human , I2D-Tarassov-PCA-Yeast2Human , I2D-Tewari-Vidal-2004-TGFb-Worm2Human , I2D-vonMering-Bork-2002-High-Yeast2Human , I2D-vonMering-Bork-2002-Low-Yeast2Human , I2D-vonMering-Bork-2002-Medium-Yeast2Human , I2D-Wang-Orkin-2006-EScmplx-Mouse2Human , I2D-Wang-Orkin-2006-EScmplxlow-Mouse2Human , I2D-Yu-Vidal-2008-GoldStd-Yeast2Human , IMID , Ingham-Pawson-2005 , Innocenti-Brown-2011 , INTERPRO , IREF-BIND , IREF-BIOGRID , IREF-DIP , IREF-HPRD , IREF-INTACT , IREF-MATRIXDB , IREF-MPPI , IREF-PUBMED , IREF-SMALL-SCALE-STUDIES , IREF-SMALL-SCALE-STUDIES

J

Jeronimo-Coulombe-2007 , Jin-Pawson-2004 , Johnson-Kerner-Wichterle-2015 , Johnson-Shoemaker-2003 , Jones-MacBeath-2006 , Joshi-Cristea-2013 , Jäger-Krogan-2011

K

Kahle-Zoghbi-2011 , Kaltenbach-Hughes-2007 , Katsogiannou-Rocchi-2014 , Kim-Gygi-2011 , Kim-Major-2015 , Kneissl-Grummt-2003 , Koch-Hermeking-2007 , Kotlyar-Jurisica-2015 , Kristensen-Foster-2012 , Kärblane-Sarmiento-2015 , Kırlı-Görlich-2015

L

Lambert-Gingras-2015 , Lamoliatte-Thibault-2014 , Lau-Ronai-2012 , Lee-Songyang-2011 , Lehner-Sanderson-2004 A , Lehner-Sanderson-2004 B , Leng-Wang-2014 , Leung-Jones-2014 , Li-Chen-2015 , Li-Dorf-2011 A , Li-Dorf-2011 B , Li-Dorf-2014 , Li-Haura-2013 , Lim-Zoghbi-2006 , Lin-Smith-2010 , Lipp-Guthrie-2015 , Liu-Wang-2012 , Llères-Lamond-2010 , Loch-Strickler-2012 , Low-Heck-2014 , Lu-Zhang-2013 , Luo-Elledge-2009

M

Mak-Moffat-2010 , Mallon-McKay-2013 , Malovannaya-Qin-2010 , Markson-Sanderson-2009 , Maréchal-Zou-2014 , Matsumoto-Nakayama-2005 , McCracken-Blencowe-2005 , McFarland-Nussbaum-2008 , Meek-Piwnica-Worms-2004 , Milev-Mouland-2012 , Miyamoto-Sato-Yanagawa-2010 , Murakawa-Landthaler-2015

N

Nakayama-Ohara-2002 , Nakayasu-Adkins-2013 , Napolitano-Meroni-2011 , Narayan-Bennett-2012 , Nathan-Goldberg-2013 , NCI_NATURE , Neganova-Lako-2011 , Newman-Keating-2003 , Nicholson-Hupp-2014 , Noble-Diehl-2008

O

Oliviero-Cagney-2015 , Olma-Pintard-2009 , Oláh-Ovádi-2011 , Oshikawa-Nakayama-2012 , Ouyang-Gill-2009

P

Panigrahi-Pati-2012 , Papp-Lamia-2015 , Perez-Hernandez-Yáñez-Mó-2013 , Perou-Botstein-1999 , Perou-Botstein-2000 , Persaud-Rotin-2009 , Petschnigg-Stagljar-2014 , PFAM , Phillips-Corn-2013 , Pichlmair-Superti-Furga-2011 , Pichlmair-Superti-Furga-2012 , Pilot-Storck-Goillot-2010 , Povlsen-Choudhary-2012

R

Ramachandran-LaBaer-2004 , Raman-Harper-2015 , Ramaswamy-Golub-2001 , Ravasi-Hayashizaki-2010 , REACTOME , Reinke-Keating-2013 , Reyniers-Taymans-2014 , Richter-Chrzanowska-Lightowlers-2010 , Rieger-Chu-2004 , Rolland-Vidal-2014 , Rosenwald-Staudt-2001 , Roth-Zlotnik-2006 , Roux-Burke-2012 , Rowbotham-Mermoud-2011 , Roy-Pardo-2014 , Roy-Parent-2013 , Rual-Vidal-2005 A , Rual-Vidal-2005 B

S

Sang-Jackson-2011 , Sato-Conaway-2004 , Schadt-Shoemaker-2004 , Scholz-Taylor-2016 , Singh-Moore-2012 , Smirnov-Cheung-2009 , So-Colwill-2015 , Soler-López-Aloy-2011 , Sowa-Harper-2009 , Stehling-Lill-2012 , Stehling-Lill-2013 , Stelzl-Wanker-2005 , Stes-Gevaert-2014 , Stuart-Kim-2003 , Suter-Wanker-2013

T

Taipale-Lindquist-2012 , Taipale-Lindquist-2014 , Takahashi-Conaway-2011 , Tarallo-Weisz-2011 , Tatham-Hay-2011 , Teixeira-Gomes-2010 , Thalappilly-

T

Dusetti-2008 , Thompson-Luchansky-2014 , Tong-Moran-2014 , Toyoshima-Grandori-2012 , Tsai-Cristea-2012

U

Udeshi-Carr-2012

V

---

van Wijk-Timmers-2009 , Vandamme-Angrand-2011 , Varjosalo-Gstaiger-2013 , Varjosalo-Superti-Furga-2013 , Venkatesan-Vidal-2009 , Vermeulen-Mann-2010 , Vinayagam-Wanker-2011 , Virok-Fülöp-2011 , Vizeacoumar-Moffat-2013

W

Wagner-Choudhary-2011 , Wallach-Kramer-2013 , Wan-Emili-2015 , Wang-Balch-2006 , Wang-Cheung-2015 , Wang-He-2008 , Wang-Maris-2006 , Wang-Xu-2015 , Wang-Yang-2011 , Weimann-Stelzl-2013 A , Weimann-Stelzl-2013 B , Weinmann-Meister-2009 , Wen-Wu-2014 , Whisenant-Salomon-2015 , Wilker-Yaffe-2007 , Willingham-Muchowski-2003 , Witt-Labeit-2008 , Wong-O'Bryan-2012 , Woods-Monteiro-2012 , Woodsmith-Sanderson-2012 , Wu-Garvey-2007 , Wu-Li-2007 , Wu-Ma-2012 , Wu-Stein-2010 , Wu-Stein-2010

X

Xiao-Lefkowitz-2007 , Xie-Cong-2013 , Xie-Green-2012 , Xu-Ye-2012

Y

Yang-Chen-2010 , Yatim-Benkirane-2012 , Yu-Chow-2013 , Yu-Vidal-2011

Z

Zanon-Pichler-2013 , Zhang-Shang-2006 , Zhang-Zou-2011 , Zhao-Krug-2005 , Zhao-Yang-2011 , Zhou-Conrads-2004 , Zhou-Hanemann-2016

Genes

Gene	Description	Rank
FAM129B	family with sequence similarity 129 member B [Source:HGNC Symbol;Acc:HGNC:25282]	N/A
LIN7B	lin-7 homolog B, crumbs cell polarity complex component [Source:HGNC Symbol;Acc:HGNC:17788]	N/A
ABCA1	ATP binding cassette subfamily A member 1 [Source:HGNC Symbol;Acc:HGNC:29]	N/A
GLS	glutaminase [Source:HGNC Symbol;Acc:HGNC:4331]	N/A
TTR	transthyretin [Source:HGNC Symbol;Acc:HGNC:12405]	N/A
APOA1	apolipoprotein A1 [Source:HGNC Symbol;Acc:HGNC:600]	1
RBP4	retinol binding protein 4 [Source:HGNC Symbol;Acc:HGNC:9922]	2
HNF4A	hepatocyte nuclear factor 4 alpha [Source:HGNC Symbol;Acc:HGNC:5024]	3
RARA	retinoic acid receptor alpha [Source:HGNC Symbol;Acc:HGNC:9864]	4
MT3	metallothionein 3 [Source:HGNC Symbol;Acc:HGNC:7408]	5
GLUL	glutamate-ammonia ligase [Source:HGNC Symbol;Acc:HGNC:4341]	6
ASIC3	acid sensing ion channel subunit 3 [Source:HGNC Symbol;Acc:HGNC:101]	7
APBA1	amyloid beta precursor protein binding family A member 1 [Source:HGNC Symbol;Acc:HGNC:578]	8
HTATSF1	HIV-1 Tat specific factor 1 [Source:HGNC Symbol;Acc:HGNC:5276]	9
TBC1D19	TBC1 domain family member 19 [Source:HGNC Symbol;Acc:HGNC:25624]	10
CASK	calcium/calmodulin dependent serine protein kinase [Source:HGNC Symbol;Acc:HGNC:1497]	11
SIRT5	sirtuin 5 [Source:HGNC Symbol;Acc:HGNC:14933]	12
KCNJ4	potassium voltage-gated channel subfamily J member 4 [Source:HGNC Symbol;Acc:HGNC:6265]	13
SP1	Sp1 transcription factor [Source:HGNC Symbol;Acc:HGNC:11205]	14
NFATC2	nuclear factor of activated T-cells 2 [Source:HGNC Symbol;Acc:HGNC:7776]	15
ARHGEF12	Rho guanine nucleotide exchange factor 12 [Source:HGNC Symbol;Acc:HGNC:14193]	16
APP	amyloid beta precursor protein [Source:HGNC Symbol;Acc:HGNC:620]	17

Gene	Description	Rank
MPP6	membrane palmitoylated protein 6 [Source:HGNC Symbol;Acc:HGNC: 18167]	18
LIN7C	lin-7 homolog C, crumbs cell polarity complex component [Source:HGNC Symbol;Acc:HGNC:17789]	19
MPP2	membrane palmitoylated protein 2 [Source:HGNC Symbol;Acc:HGNC: 7220]	20

Networks

Physical Interactions 67.64%

Matsumoto-Nakayama-2005 5.54%

Large-scale analysis of the human ubiquitin-related proteome. Matsumoto et al (2005). *Proteomics*

Physical Interactions with 311 interactions from BioGRID

Singh-Moore-2012 4.12%

The cellular EJC interactome reveals higher-order mRNP structure and an EJC-SR protein nexus. Singh et al (2012). *Cell*

Physical Interactions with 301 interactions from iRefIndex

Petschnigg-Stagljar-2014 3.90%

The mammalian-membrane two-hybrid assay (MaMTH) for probing membrane-protein interactions in human cells. Petschnigg et al (2014). *Nat Methods*

Physical Interactions with 122 interactions from BioGRID

Whisenant-Salomon-2015 2.70%

The Activation-Induced Assembly of an RNA/Protein Interactome Centered on the Splicing Factor U2AF2 Regulates Gene Expression in Human CD4 T Cells. Whisenant et al (2015). *PLoS One*

Physical Interactions with 237 interactions from BioGRID

Lu-Zhang-2013 2.43%

The HECT type ubiquitin ligase NEDL2 is degraded by anaphase-promoting complex/cyclosome (APC/C)-Cdh1, and its tight regulation maintains the metaphase to anaphase transition. Lu et al (2013). *J Biol Chem*

Physical Interactions with 281 interactions from iRefIndex

Phillips-Corn-2013 1.88%

Conformational dynamics control ubiquitin-deubiquitinase interactions and influence in vivo signaling. Phillips et al (2013). *Proc Natl Acad Sci U S A*

Physical Interactions with 134 interactions from BioGRID

Wagner-Choudhary-2011 1.86%

A proteome-wide, quantitative survey of in vivo ubiquitylation sites reveals widespread regulatory roles. Wagner et al (2011). *Mol Cell Proteomics*

Physical Interactions with 1,158 interactions from iRefIndex

Behzadnia-Lührmann-2007 1.55%

Composition and three-dimensional EM structure of double affinity-purified, human prespliceosomal A complexes. Behzadnia et al (2007). *EMBO J*

Physical Interactions with 112 interactions from iRefIndex

Leung-Jones-2014 1.43%

Enhanced prediction of Src homology 2 (SH2) domain binding potentials using a fluorescence polarization-derived c-Met, c-Kit, ErbB, and androgen receptor interactome. Leung et al (2014). *Mol Cell Proteomics*

Physical Interactions with 190 interactions from iRefIndex

Maréchal-Zou-2014 1.41%

PRP19 transforms into a sensor of RPA-ssDNA after DNA damage and drives ATR activation via a ubiquitin-mediated circuitry. Maréchal et al (2014). *Mol Cell*

Physical Interactions with 976 interactions from iRefIndex

Berggård-James-2006 1.40%

Physical Interactions 67.64%

Berggård-James-2006

140 mouse brain proteins identified by Ca2+-calmodulin affinity chromatography and tandem mass spectrometry. Berggård et al (2006). *J Proteome Res*

Physical Interactions with 152 interactions from iRefIndex

Neganova-Lako-2011 1.28%

An important role for CDK2 in G1 to S checkpoint activation and DNA damage response in human embryonic stem cells. Neganova et al (2011). *Stem Cells*

Physical Interactions with 393 interactions from iRefIndex

McCracken-Blencowe-2005 1.21%

Proteomic analysis of SRm160-containing complexes reveals a conserved association with cohesin. McCracken et al (2005). *J Biol Chem*

Physical Interactions with 184 interactions from iRefIndex

Mak-Moffat-2010 1.16%

A lentiviral functional proteomics approach identifies chromatin remodeling complexes important for the induction of pluripotency. Mak et al (2010). *Mol Cell Proteomics*

Physical Interactions with 110 interactions from BioGRID

Murakawa-Landthaler-2015 1.03%

RC3H1 post-transcriptionally regulates A20 mRNA and modulates the activity of the IKK/NF- B pathway. Murakawa et al (2015). *Nat Commun*

Physical Interactions with 155 interactions from BioGRID

Llères-Lamond-2010 1.00%

Direct interaction between hnRNP-M and CDC5L/PLRG1 proteins affects alternative splice site choice. Llères et al (2010). *EMBO Rep*

Physical Interactions with 848 interactions from BioGRID

Nicholson-Hupp-2014 0.99%

A systems wide mass spectrometric based linear motif screen to identify dominant in-vivo interacting proteins for the ubiquitin ligase MDM2. Nicholson et al (2014). *Cell Signal*

Physical Interactions with 382 interactions from iRefIndex

Jones-MacBeath-2006 0.93%

A quantitative protein interaction network for the ErbB receptors using protein microarrays. Jones et al (2006). *Nature*

Physical Interactions with 151 interactions from iRefIndex

McFarland-Nussbaum-2008 0.87%

Proteomics analysis identifies phosphorylation-dependent alpha-synuclein protein interactions. McFarland et al (2008). *Mol Cell Proteomics*

Physical Interactions with 157 interactions from iRefIndex

Guarani-Harper-2014 0.83%

TIMMDC1/C3orf1 functions as a membrane-embedded mitochondrial complex I assembly factor through association with the MCIA complex. Guarani et al (2014). *Mol Cell Biol*

Physical Interactions with 323 interactions from BioGRID

Hill-Livingston-2014 0.83%

Systematic screening reveals a role for BRCA1 in the response to transcription-associated DNA damage. Hill et al (2014). *Genes Dev*

Physical Interactions with 125 interactions from iRefIndex

Physical Interactions 67.64%

Bett-Hay-2013 0.79%

The P-body component USP52/PAN2 is a novel regulator of HIF1A mRNA stability. Bett et al (2013). *Biochem J*

Physical Interactions with 319 interactions from iRefIndex

Stes-Gevaert-2014 0.73%

A COFRADIC protocol to study protein ubiquitination. Stes et al (2014). *J Proteome Res*

Physical Interactions with 1,327 interactions from iRefIndex

Danielsen-Nielsen-2011 0.67%

Mass spectrometric analysis of lysine ubiquitylation reveals promiscuity at site level. Danielsen et al (2011). *Mol Cell Proteomics*

Physical Interactions with 2,479 interactions from iRefIndex

Jeronimo-Coulombe-2007 0.65%

Systematic analysis of the protein interaction network for the human transcription machinery reveals the identity of the 7SK capping enzyme. Jeronimo et al (2007). *Mol Cell*

Physical Interactions with 699 interactions from BioGRID

Xu-Ye-2012 0.65%

SGTA recognizes a noncanonical ubiquitin-like domain in the Bag6-Ubl4A-Trc35 complex to promote endoplasmic reticulum-associated degradation. Xu et al (2012). *Cell Rep*

Physical Interactions with 225 interactions from iRefIndex

Gloeckner-Ueffing-2007 0.62%

A novel tandem affinity purification strategy for the efficient isolation and characterisation of native protein complexes. Gloeckner et al (2007). *Proteomics*

Physical Interactions with 100 interactions from BioGRID

Fogeron-Lange-2013 0.61%

LGALS3BP regulates centriole biogenesis and centrosome hypertrophy in cancer cells. Fogeron et al (2013). *Nat Commun*

Physical Interactions with 1,492 interactions from BioGRID

Freibaum-Taylor-2010 0.60%

Global analysis of TDP-43 interacting proteins reveals strong association with RNA splicing and translation machinery. Freibaum et al (2010). *J Proteome Res*

Physical Interactions with 216 interactions from iRefIndex

Zhou-Conrads-2004 0.59%

"An investigation into the human serum ""interactome""." Zhou et al (2004). *Electrophoresis*

Physical Interactions with 158 interactions from iRefIndex

Brehme-Superti-Furga-2009 0.59%

Charting the molecular network of the drug target Bcr-Abl. Brehme et al (2009). *Proc Natl Acad Sci U S A*

Physical Interactions with 578 interactions from iRefIndex

Yu-Chow-2013 0.59%

VCP phosphorylation-dependent interaction partners prevent apoptosis in Helicobacter pylori-infected gastric epithelial cells. Yu et al (2013). *PLoS One*

Physical Interactions with 272 interactions from iRefIndex

Hegele-Stelzl-2012 B 0.55%

Dynamic protein-protein interaction wiring of the human spliceosome. Hegele et al (2012). *Mol Cell*

Physical Interactions with 600 interactions from BioGRID

Physical Interactions 67.64%

Weinmann-Meister-2009 0.54%

Importin 8 is a gene silencing factor that targets argonaute proteins to distinct mRNAs. Weinmann et al (2009). *Cell*

Physical Interactions with 96 interactions from BioGRID

Narayan-Bennett-2012 0.53%

Short-chain 3-hydroxyacyl-coenzyme A dehydrogenase associates with a protein super-complex integrating multiple metabolic pathways. Narayan et al (2012). *PLoS One*

Physical Interactions with 110 interactions from BioGRID

Udeshi-Carr-2012 0.52%

Methods for quantification of in vivo changes in protein ubiquitination following proteasome and deubiquitinase inhibition. Udeshi et al (2012). *Mol Cell Proteomics*

Physical Interactions with 554 interactions from iRefIndex

Kristensen-Foster-2012 0.51%

A high-throughput approach for measuring temporal changes in the interactome. Kristensen et al (2012). *Nat Methods*

Physical Interactions with 7,115 interactions from BioGRID

Agrawal-Sedivy-2010 0.50%

Proteomic profiling of Myc-associated proteins. Agrawal et al (2010). *Cell Cycle*

Physical Interactions with 104 interactions from iRefIndex

Varjosalo-Superti-Furga-2013 0.49%

Interlaboratory reproducibility of large-scale human protein-complex analysis by standardized AP-MS. Varjosalo et al (2013). *Nat Methods*

Physical Interactions with 483 interactions from BioGRID

Rowbotham-Mermoud-2011 0.46%

Maintenance of silent chromatin through replication requires SWI/SNF-like chromatin remodeler SMARCAD1. Rowbotham et al (2011). *Mol Cell*

Physical Interactions with 114 interactions from iRefIndex

Arroyo-Aloy-2014 0.44%

Charting the molecular links between driver and susceptibility genes in colorectal cancer. Arroyo et al (2014). *Biochem Biophys Res Commun*

Physical Interactions with 598 interactions from iRefIndex

Barr-Knapp-2009 0.43%

Large-scale structural analysis of the classical human protein tyrosine phosphatome. Barr et al (2009). *Cell*

Physical Interactions with 164 interactions from iRefIndex

Emdal-Olsen-2015 0.41%

Temporal proteomics of NGF-TrkA signaling identifies an inhibitory role for the E3 ligase Cbl-b in neuroblastoma cell differentiation. Emdal et al (2015). *Sci Signal*

Physical Interactions with 1,919 interactions from BioGRID

Cox-Rizzino-2013 0.40%

The SOX2-interactome in brain cancer cells identifies the requirement of MSI2 and USP9X for the growth of brain tumor cells. Cox et al (2013). *PLoS One*

Physical Interactions with 280 interactions from iRefIndex

Giannone-Liu-2010 0.39%

Physical Interactions 67.64%

Giannone-Liu-2010

The protein network surrounding the human telomere repeat binding factors TRF1, TRF2, and POT1. Giannone et al (2010). *PLoS One*

Physical Interactions with 279 interactions from iRefIndex

Havugimana-Emili-2012 0.39%

A census of human soluble protein complexes. Havugimana et al (2012). *Cell*

Physical Interactions with 13,716 interactions from BioGRID

IREF-DIP 0.39%

Physical Interactions with 4,470 interactions from iRefIndex

Li-Dorf-2011 A 0.39%

Mapping a dynamic innate immunity protein interaction network regulating type I interferon production. Li et al (2011). *Immunity*

Physical Interactions with 400 interactions from BioGRID

Persaud-Rotin-2009 0.38%

Comparison of substrate specificity of the ubiquitin ligases Nedd4 and Nedd4-2 using proteome arrays. Persaud et al (2009). *Mol Syst Biol*

Physical Interactions with 239 interactions from iRefIndex

Foerster-Ritter-2013 0.37%

Characterization of the EGFR interactome reveals associated protein complex networks and intracellular receptor dynamics. Foerster et al (2013). *Proteomics*

Physical Interactions with 159 interactions from iRefIndex

Zhao-Krug-2005 0.36%

Human ISG15 conjugation targets both IFN-induced and constitutively expressed proteins functioning in diverse cellular pathways. Zhao et al (2005). *Proc Natl Acad Sci U S A*

Physical Interactions with 150 interactions from iRefIndex

Wan-Emili-2015 0.36%

Panorama of ancient metazoan macromolecular complexes. Wan et al (2015). *Nature*

Physical Interactions with 16,682 interactions from BioGRID

Fenner-Prehn-2010 0.36%

Expanding the substantial interactome of NEMO using protein microarrays. Fenner et al (2010). *PLoS One*

Physical Interactions with 103 interactions from iRefIndex

Roux-Burke-2012 0.35%

A promiscuous biotin ligase fusion protein identifies proximal and interacting proteins in mammalian cells. Roux et al (2012). *J Cell Biol*

Physical Interactions with 115 interactions from iRefIndex

Loch-Strickler-2012 0.33%

A microarray of ubiquitylated proteins for profiling deubiquitylase activity reveals the critical roles of both chain and substrate. Loch et al (2012). *Biochim Biophys Acta*

Physical Interactions with 145 interactions from iRefIndex

IREF-MATRIXDB 0.32%

Physical Interactions with 249 interactions from iRefIndex

Physical Interactions 67.64%

Diner-Cristea-2015 0.32%

Interactions of the Antiviral Factor Interferon Gamma-Inducible Protein 16 (IFI16) Mediate Immune Signaling and Herpes Simplex Virus-1 Immunosuppression. Diner et al (2015). *Mol Cell Proteomics*

Physical Interactions with 332 interactions from BioGRID

Hein-Mann-2015 0.31%

A human interactome in three quantitative dimensions organized by stoichiometries and abundances. Hein et al (2015). *Cell*

Physical Interactions with 27,044 interactions from BioGRID

Lipp-Guthrie-2015 0.30%

SR protein kinases promote splicing of nonconsensus introns. Lipp et al (2015). *Nat Struct Mol Biol*

Physical Interactions with 386 interactions from BioGRID

Li-Chen-2015 0.30%

Proteomic analyses reveal distinct chromatin-associated and soluble transcription factor complexes. Li et al (2015). *Mol Syst Biol*

Physical Interactions with 1,809 interactions from BioGRID

Wang-Xu-2015 0.29%

Interaction of amyotrophic lateral sclerosis/frontotemporal lobar degeneration-associated fused-in-sarcoma with proteins involved in metabolic and protein degradation pathways. Wang et al (2015). *Neurobiol Aging*

Physical Interactions with 192 interactions from iRefIndex

Roy-Pardo-2014 0.29%

hnRNPA1 couples nuclear export and translation of specific mRNAs downstream of FGF-2/S6K2 signalling. Roy et al (2014). *Nucleic Acids Res*

Physical Interactions with 386 interactions from BioGRID

Kotlyar-Jurisica-2015 0.28%

In silico prediction of physical protein interactions and characterization of interactome orphans. Kotlyar et al (2015). *Nat Methods*

Physical Interactions with 121 interactions from BioGRID

Barrios-Rodiles-Wrana-2005 0.27%

High-throughput mapping of a dynamic signaling network in mammalian cells. Barrios-Rodiles et al (2005). *Science*

Physical Interactions with 552 interactions from iRefIndex

BIOGRID-SMALL-SCALE-STUDIES 0.27%

Physical Interactions with 58,871 interactions from BioGRID

Sowa-Harper-2009 0.26%

Defining the human deubiquitinating enzyme interaction landscape. Sowa et al (2009). *Cell*

Physical Interactions with 1,509 interactions from BioGRID

Ramachandran-LaBaer-2004 0.26%

Self-assembling protein microarrays. Ramachandran et al (2004). *Science*

Physical Interactions with 112 interactions from iRefIndex

Oshikawa-Nakayama-2012 0.26%

Proteome-wide identification of ubiquitylation sites by conjugation of engineered lysine-less ubiquitin. Oshikawa et al (2012). *J Proteome Res*

Physical Interactions with 116 interactions from iRefIndex

Bonacci-Soubeyran-2014 0.26%

Physical Interactions 67.64%

Bonacci-Soubeyran-2014

Identification of new mechanisms of cellular response to chemotherapy by tracking changes in post-translational modifications by ubiquitin and ubiquitin-like proteins. Bonacci et al (2014). *J Proteome Res*

Physical Interactions with 937 interactions from iRefIndex

Kim-Gygi-2011 0.26%

Systematic and quantitative assessment of the ubiquitin-modified proteome. Kim et al (2011). *Mol Cell*

Physical Interactions with 1,345 interactions from iRefIndex

Taipale-Lindquist-2012 0.26%

Quantitative analysis of HSP90-client interactions reveals principles of substrate recognition. Taipale et al (2012). *Cell*

Physical Interactions with 716 interactions from iRefIndex

Wilker-Yaffe-2007 0.25%

14-3-3sigma controls mitotic translation to facilitate cytokinesis. Wilker et al (2007). *Nature*

Physical Interactions with 110 interactions from iRefIndex

Jin-Pawson-2004 0.24%

Proteomic, functional, and domain-based analysis of in vivo 14-3-3 binding proteins involved in cytoskeletal regulation and cellular organization. Jin et al (2004). *Curr Biol*

Physical Interactions with 236 interactions from iRefIndex

Oliviero-Cagney-2015 0.24%

The variant Polycomb Repressor Complex 1 component PCGF1 interacts with a pluripotency sub-network that includes DPPA4, a regulator of embryogenesis. Oliviero et al (2015). *Sci Rep*

Physical Interactions with 675 interactions from BioGRID

IREF-BIND 0.24%

Physical Interactions with 3,659 interactions from iRefIndex

Hutchins-Peters-2010 0.24%

Systematic analysis of human protein complexes identifies chromosome segregation proteins. Hutchins et al (2010). *Science*

Physical Interactions with 1,783 interactions from BioGRID

Tong-Moran-2014 0.23%

Proteomic analysis of the epidermal growth factor receptor (EGFR) interactome and post-translational modifications associated with receptor endocytosis in response to EGF and stress. Tong et al (2014). *Mol Cell Proteomics*

Physical Interactions with 271 interactions from iRefIndex

IREF-MPPI 0.23%

Physical Interactions with 382 interactions from iRefIndex

Li-Haura-2013 0.21%

Perturbation of the mutated EGFR interactome identifies vulnerabilities and resistance mechanisms. Li et al (2013). *Mol Syst Biol*

Physical Interactions with 403 interactions from BioGRID

Woods-Monteiro-2012 0.21%

Charting the landscape of tandem BRCT domain-mediated protein interactions. Woods et al (2012). *Sci Signal*

Physical Interactions with 919 interactions from iRefIndex

Xie-Cong-2013 0.19%

Physical Interactions 67.64%

Xie-Cong-2013

Deubiquitinase FAM/USP9X interacts with the E3 ubiquitin ligase SMURF1 protein and protects it from ligase activity-dependent self-degradation. Xie et al (2013). *J Biol Chem*

Physical Interactions with 168 interactions from iRefIndex

Malovannaya-Qin-2010 0.19%

Streamlined analysis schema for high-throughput identification of endogenous protein complexes. Malovannaya et al (2010). *Proc Natl Acad Sci U S A*

Physical Interactions with 224 interactions from iRefIndex

Chen-Zhang-2013 0.18%

Quantitative study of the interactome of PKC involved in the EGF-induced tumor cell chemotaxis. Chen et al (2013). *J Proteome Res*

Physical Interactions with 180 interactions from iRefIndex

Koch-Hermeking-2007 0.17%

Large-scale identification of c-MYC-associated proteins using a combined TAP/MudPIT approach. Koch et al (2007). *Cell Cycle*

Physical Interactions with 175 interactions from iRefIndex

Xiao-Lefkowitz-2007 0.17%

Functional specialization of beta-arrestin interactions revealed by proteomic analysis. Xiao et al (2007). *Proc Natl Acad Sci U S A*

Physical Interactions with 402 interactions from iRefIndex

Napolitano-Meroni-2011 0.17%

Functional interactions between ubiquitin E2 enzymes and TRIM proteins. Napolitano et al (2011). *Biochem J*

Physical Interactions with 81 interactions from BioGRID

IREF-PUBMED 0.16%

Physical Interactions with 571 interactions from iRefIndex

Varjosalo-Gstaiger-2013 0.16%

The protein interaction landscape of the human CMGC kinase group. Varjosalo et al (2013). *Cell Rep*

Physical Interactions with 936 interactions from iRefIndex

So-Colwill-2015 0.15%

Integrative analysis of kinase networks in TRAIL-induced apoptosis provides a source of potential targets for combination therapy. So et al (2015). *Sci Signal*

Physical Interactions with 647 interactions from BioGRID

Scholz-Taylor-2016 0.14%

FIH Regulates Cellular Metabolism through Hydroxylation of the Deubiquitinase OTUB1. Scholz et al (2016). *PLoS Biol*

Physical Interactions with 134 interactions from BioGRID

Huttlin-Gygi-2015 0.14%

The BioPlex Network: A Systematic Exploration of the Human Interactome. Huttlin et al (2015). *Cell*

Physical Interactions with 23,399 interactions from BioGRID

Ingham-Pawson-2005 0.14%

WW domains provide a platform for the assembly of multiprotein networks. Ingham et al (2005). *Mol Cell Biol*

Physical Interactions with 299 interactions from iRefIndex

Physical Interactions 67.64%

Tsai-Cristea-2012 0.13%

Functional proteomics establishes the interaction of SIRT7 with chromatin remodeling complexes and expands its role in regulation of RNA polymerase I transcription. Tsai et al (2012). *Mol Cell Proteomics*

Physical Interactions with 655 interactions from iRefIndex

Yatim-Benkirane-2012 0.12%

NOTCH1 nuclear interactome reveals key regulators of its transcriptional activity and oncogenic function. Yatim et al (2012). *Mol Cell*

Physical Interactions with 131 interactions from iRefIndex

Thompson-Luchansky-2014 0.12%

Quantitative Lys- -Gly-Gly (diGly) proteomics coupled with inducible RNAi reveals ubiquitin-mediated proteolysis of DNA damage-inducible transcript 4 (DDIT4) by the E3 ligase HUWE1. Thompson et al (2014). *J Biol Chem*

Physical Interactions with 552 interactions from iRefIndex

Floyd-Pagliarini-2016 0.12%

Mitochondrial Protein Interaction Mapping Identifies Regulators of Respiratory Chain Function. Floyd et al (2016). *Mol Cell*

Physical Interactions with 1,508 interactions from BioGRID

Bandyopadhyay-Ideker-2010 0.12%

A human MAP kinase interactome. Bandyopadhyay et al (2010). *Nat Methods*

Physical Interactions with 611 interactions from iRefIndex

van Wijk-Timmers-2009 0.11%

A comprehensive framework of E2-RING E3 interactions of the human ubiquitin-proteasome system. van Wijk et al (2009). *Mol Syst Biol*

Physical Interactions with 301 interactions from iRefIndex

IREF-INTACT 0.11%

Physical Interactions with 56,297 interactions from iRefIndex

Lee-Songyang-2011 0.11%

Genome-wide YFP fluorescence complementation screen identifies new regulators for telomere signaling in human cells. Lee et al (2011). *Mol Cell Proteomics*

Physical Interactions with 604 interactions from iRefIndex

Grossmann-Stelzl-2015 0.11%

Phospho-tyrosine dependent protein-protein interaction network. Grossmann et al (2015). *Mol Syst Biol*

Physical Interactions with 622 interactions from BioGRID

Liu-Wang-2012 0.11%

Proteomic identification of common SCF ubiquitin ligase FBXO6-interacting glycoproteins in three kinds of cells. Liu et al (2012). *J Proteome Res*

Physical Interactions with 586 interactions from iRefIndex

Povlsen-Choudhary-2012 0.10%

Systems-wide analysis of ubiquitylation dynamics reveals a key role for PAF15 ubiquitylation in DNA-damage bypass. Povlsen et al (2012). *Nat Cell Biol*

Physical Interactions with 562 interactions from iRefIndex

Oláh-Ovádi-2011 0.10%

Interactions of pathological hallmark proteins: tubulin polymerization promoting protein/p25, beta-amyloid, and alpha-synuclein. Oláh et al (2011). *J Biol Chem*

Physical Interactions 67.64%

Oláh-Ovádi-2011

Physical Interactions with 1,853 interactions from iRefIndex

Hayes-Urbé-2012 0.10%

Direct and indirect control of mitogen-activated protein kinase pathway-associated components, BRAP/IMP E3 ubiquitin ligase and CRAF/RAF1 kinase, by the deubiquitylating enzyme USP15. Hayes et al (2012). *J Biol Chem*

Physical Interactions with 107 interactions from iRefIndex

Joshi-Cristea-2013 0.10%

The functional interactome landscape of the human histone deacetylase family. Joshi et al (2013). *Mol Syst Biol*

Physical Interactions with 310 interactions from iRefIndex

Roy-Parent-2013 0.10%

Novel, gel-free proteomics approach identifies RNF5 and JAMP as modulators of GPCR stability. Roy et al (2013). *Mol Endocrinol*

Physical Interactions with 155 interactions from iRefIndex

Ewing-Figeys-2007 0.10%

Large-scale mapping of human protein-protein interactions by mass spectrometry. Ewing et al (2007). *Mol Syst Biol*

Physical Interactions with 5,362 interactions from iRefIndex

Zanon-Pichler-2013 0.09%

Profiling of Parkin-binding partners using tandem affinity purification. Zanon et al (2013). *PLoS One*

Physical Interactions with 187 interactions from iRefIndex

Brajenovic-Drewes-2004 0.09%

Comprehensive proteomic analysis of human Par protein complexes reveals an interconnected protein network. Brajenovic et al (2004). *J Biol Chem*

Physical Interactions with 141 interactions from iRefIndex

Greco-Cristea-2011 0.09%

Nuclear import of histone deacetylase 5 by requisite nuclear localization signal phosphorylation. Greco et al (2011). *Mol Cell Proteomics*

Physical Interactions with 240 interactions from iRefIndex

Wu-Li-2007 0.09%

Systematic identification of SH3 domain-mediated human protein-protein interactions by peptide array target screening. Wu et al (2007). *Proteomics*

Physical Interactions with 927 interactions from iRefIndex

Behrends-Harper-2010 0.09%

Network organization of the human autophagy system. Behrends et al (2010). *Nature*

Physical Interactions with 751 interactions from iRefIndex

Nakayasu-Adkins-2013 0.09%

Evaluation of selected binding domains for the analysis of ubiquitinated proteomes. Nakayasu et al (2013). *J Am Soc Mass Spectrom*

Physical Interactions with 880 interactions from iRefIndex

Ouyang-Gill-2009 0.08%

Direct binding of CoREST1 to SUMO-2/3 contributes to gene-specific repression by the LSD1/CoREST1/HDAC complex. Ouyang et al (2009). *Mol Cell*

Physical Interactions 67.64%

Ouyang-Gill-2009

Physical Interactions with 105 interactions from BioGRID

Perez-Hernandez-Yáñez-Mó-2013 0.08%

The intracellular interactome of tetraspanin-enriched microdomains reveals their function as sorting machineries toward exosomes. Perez-Hernandez et al (2013). *J Biol Chem*

Physical Interactions with 450 interactions from iRefIndex

Reyniers-Taymans-2014 0.08%

Differential protein-protein interactions of LRRK1 and LRRK2 indicate roles in distinct cellular signaling pathways. Reyniers et al (2014). *J Neurochem*

Physical Interactions with 102 interactions from iRefIndex

Bruderer-Hay-2011 0.08%

Purification and identification of endogenous polySUMO conjugates. Bruderer et al (2011). *EMBO Rep*

Physical Interactions with 106 interactions from iRefIndex

Couzens-Gingras-2013 0.08%

Protein interaction network of the mammalian Hippo pathway reveals mechanisms of kinase-phosphatase interactions. Couzens et al (2013). *Sci Signal*

Physical Interactions with 364 interactions from BioGRID

Bennett-Harper-2010 0.08%

Dynamics of cullin-RING ubiquitin ligase network revealed by systematic quantitative proteomics. Bennett et al (2010). *Cell*

Physical Interactions with 4,367 interactions from BioGRID

Christianson-Kopito-2011 0.07%

Defining human ERAD networks through an integrative mapping strategy. Christianson et al (2011). *Nat Cell Biol*

Physical Interactions with 260 interactions from iRefIndex

Albers-Koegl-2005 0.07%

Automated yeast two-hybrid screening for nuclear receptor-interacting proteins. Albers et al (2005). *Mol Cell Proteomics*

Physical Interactions with 238 interactions from iRefIndex

Thalappilly-Dusetti-2008 0.07%

Identification of multi-SH3 domain-containing protein interactome in pancreatic cancer: a yeast two-hybrid approach. Thalappilly et al (2008). *Proteomics*

Physical Interactions with 104 interactions from iRefIndex

Gupta-Pelletier-2015 0.07%

A Dynamic Protein Interaction Landscape of the Human Centrosome-Cilium Interface. Gupta et al (2015). *Cell*

Physical Interactions with 307 interactions from BioGRID

Vinayagam-Wanker-2011 0.06%

A directed protein interaction network for investigating intracellular signal transduction. Vinayagam et al (2011). *Sci Signal*

Physical Interactions with 2,576 interactions from BioGRID

Li-Dorf-2014 0.06%

TRIM65 regulates microRNA activity by ubiquitination of TNRC6. Li et al (2014). *Proc Natl Acad Sci U S A*

Physical Interactions with 470 interactions from iRefIndex

Katsogiannou-Rocchi-2014 0.06%

Physical Interactions 67.64%

Katsogiannou-Rocchi-2014

The functional landscape of Hsp27 reveals new cellular processes such as DNA repair and alternative splicing and proposes novel anticancer targets. Katsogiannou et al (2014). *Mol Cell Proteomics*

Physical Interactions with 217 interactions from iRefIndex

Arbuckle-Grant-2010 0.05%

The SH3 domain of postsynaptic density 95 mediates inflammatory pain through phosphatidylinositol-3-kinase recruitment. Arbuckle et al (2010). *EMBO Rep*

Physical Interactions with 268 interactions from iRefIndex

Bantscheff-Drewes-2011 0.05%

Chemoproteomics profiling of HDAC inhibitors reveals selective targeting of HDAC complexes. Bantscheff et al (2011). *Nat Biotechnol*

Physical Interactions with 103 interactions from BioGRID

Low-Heck-2014 0.04%

A systems-wide screen identifies substrates of the SCF TrCP ubiquitin ligase. Low et al (2014). *Sci Signal*

Physical Interactions with 221 interactions from BioGRID

Lau-Ronai-2012 0.04%

PKC promotes oncogenic functions of ATF2 in the nucleus while blocking its apoptotic function at mitochondria. Lau et al (2012). *Cell*

Physical Interactions with 134 interactions from iRefIndex

Kırlı-Görlich-2015 0.04%

A deep proteomics perspective on CRM1-mediated nuclear export and nucleocytoplasmic partitioning. Kırlı et al (2015). *Elife*

Physical Interactions with 1,036 interactions from BioGRID

Woodsmith-Sanderson-2012 0.04%

Systematic analysis of dimeric E3-RING interactions reveals increased combinatorial complexity in human ubiquitination networks. Woodsmith et al (2012). *Mol Cell Proteomics*

Physical Interactions with 212 interactions from iRefIndex

Zhang-Zou-2011 0.03%

A bead-based approach for large-scale identification of in vitro kinase substrates. Zhang et al (2011). *Proteomics*

Physical Interactions with 162 interactions from iRefIndex

Leng-Wang-2014 0.03%

A proteomics strategy for the identification of FAT10-modified sites by mass spectrometry. Leng et al (2014). *J Proteome Res*

Physical Interactions with 144 interactions from iRefIndex

Bogachek-Weigel-2014 0.02%

Sumoylation pathway is required to maintain the basal breast cancer subtype. Bogachek et al (2014). *Cancer Cell*

Physical Interactions with 134 interactions from iRefIndex

Hanson-Clayton-2014 0.02%

Identifying biological pathways that underlie primordial short stature using network analysis. Hanson et al (2014). *J Mol Endocrinol*

Physical Interactions with 1,687 interactions from iRefIndex

Markson-Sanderson-2009 0.02%

Analysis of the human E2 ubiquitin conjugating enzyme protein interaction network. Markson et al (2009). *Genome Res*

Physical Interactions 67.64%

Markson-Sanderson-2009

Physical Interactions with 700 interactions from iRefIndex

Golebiowski-Hay-2009 0.02%

System-wide changes to SUMO modifications in response to heat shock. Golebiowski et al (2009). *Sci Signal*

Physical Interactions with 351 interactions from iRefIndex

Yang-Chen-2010 0.02%

Proteomic dissection of cell type-specific H2AX-interacting protein complex associated with hepatocellular carcinoma. Yang et al (2010). *J Proteome Res*

Physical Interactions with 100 interactions from BioGRID

Ravasi-Hayashizaki-2010 0.02%

An atlas of combinatorial transcriptional regulation in mouse and man. Ravasi et al (2010). *Cell*

Physical Interactions with 635 interactions from iRefIndex

Arroyo-Aloy-2015 0.02%

Systematic identification of molecular links between core and candidate genes in breast cancer. Arroyo et al (2015). *J Mol Biol*

Physical Interactions with 600 interactions from iRefIndex

IREF-HPRD 0.02%

Physical Interactions with 34,206 interactions from iRefIndex

Pichlmair-Superti-Furga-2012 0.02%

Viral immune modulators perturb the human molecular network by common and unique strategies. Pichlmair et al (2012). *Nature*

Physical Interactions with 14 interactions from BioGRID

Virok-Fülöp-2011 0.02%

Protein array based interactome analysis of amyloid- indicates an inhibition of protein translation. Virok et al (2011). *J Proteome Res*

Physical Interactions with 299 interactions from BioGRID

Vermeulen-Mann-2010 0.01%

Quantitative interaction proteomics and genome-wide profiling of epigenetic histone marks and their readers. Vermeulen et al (2010). *Cell*

Physical Interactions with 131 interactions from iRefIndex

Grant-2010 0.01%

Identification of SUMOylated proteins in neuroblastoma cells after treatment with hydrogen peroxide or ascorbate. Grant (2010). *BMB Rep*

Physical Interactions with 114 interactions from iRefIndex

Meek-Piwnica-Worms-2004 0.01%

Comprehensive proteomic analysis of interphase and mitotic 14-3-3-binding proteins. Meek et al (2004). *J Biol Chem*

Physical Interactions with 359 interactions from iRefIndex

Soler-López-Aloy-2011 0.01%

Interactome mapping suggests new mechanistic details underlying Alzheimer's disease. Soler-López et al (2011). *Genome Res*

Physical Interactions with 312 interactions from iRefIndex

Yu-Vidal-2011 0.01%

Next-generation sequencing to generate interactome datasets. Yu et al (2011). *Nat Methods*

Physical Interactions — 67.64%

Yu-Vidal-2011

Physical Interactions with 1,108 interactions from BioGRID

Weimann-Stelzl-2013 A — 0.00%

A Y2H-seq approach defines the human protein methyltransferase interactome. Weimann et al (2013). *Nat Methods*

Physical Interactions with 114 interactions from BioGRID

Wong-O'Bryan-2012 — 0.00%

Intersectin (ITSN) family of scaffolds function as molecular hubs in protein interaction networks. Wong et al (2012). *PLoS One*

Physical Interactions with 111 interactions from iRefIndex

Kim-Major-2015 — 0.00%

Substrate trapping proteomics reveals targets of the TrCP2/FBXW11 ubiquitin ligase. Kim et al (2015). *Mol Cell Biol*

Physical Interactions with 114 interactions from iRefIndex

Co-expression — 13.50%

Ramaswamy-Golub-2001 — 1.02%

Multiclass cancer diagnosis using tumor gene expression signatures. Ramaswamy et al (2001). *Proc Natl Acad Sci U S A*

Co-expression with 275,113 interactions from supplementary material

Wang-Maris-2006 — 0.95%

Integrative genomics identifies distinct molecular classes of neuroblastoma and shows that multiple genes are targeted by regional alterations in DNA copy number. Wang et al (2006). *Cancer Res*

Co-expression with 264,023 interactions from GEO

Mallon-McKay-2013 — 0.86%

StemCellDB: the human pluripotent stem cell database at the National Institutes of Health. Mallon et al (2013). *Stem Cell Res*

Co-expression with 585,265 interactions from GEO

Bild-Nevins-2006 B — 0.85%

Oncogenic pathway signatures in human cancers as a guide to targeted therapies. Bild et al (2006). *Nature*

Co-expression with 280,683 interactions from GEO

Burington-Shaughnessy-2008 — 0.80%

Tumor cell gene expression changes following short-term in vivo exposure to single agent chemotherapeutics are related to survival in multiple myeloma. Burington et al (2008). *Clin Cancer Res*

Co-expression with 290,538 interactions from GEO

Dobbin-Giordano-2005 — 0.78%

Interlaboratory comparability study of cancer gene expression analysis using oligonucleotide microarrays. Dobbin et al (2005). *Clin Cancer Res*

Co-expression with 444,931 interactions from GEO

Bahr-Bowler-2013 — 0.70%

Peripheral blood mononuclear cell gene expression in chronic obstructive pulmonary disease. Bahr et al (2013). *Am J Respir Cell Mol Biol*

Co-expression with 274,949 interactions from GEO

Alizadeh-Staudt-2000 — 0.69%

Distinct types of diffuse large B-cell lymphoma identified by gene expression profiling. Alizadeh et al (2000). *Nature*

Co-expression with 90,336 interactions from supplementary material

Co-expression 13.50%

Innocenti-Brown-2011 0.69%

Identification, replication, and functional fine-mapping of expression quantitative trait loci in primary human liver tissue. Innocenti et al (2011). *PLoS Genet*

Co-expression with 603,765 interactions from GEO

Rieger-Chu-2004 0.66%

Toxicity from radiation therapy associated with abnormal transcriptional responses to DNA damage. Rieger et al (2004). *Proc Natl Acad Sci U S A*

Co-expression with 259,974 interactions from GEO

Noble-Diehl-2008 0.65%

Regional variation in gene expression in the healthy colon is dysregulated in ulcerative colitis. Noble et al (2008). *Gut*

Co-expression with 661,539 interactions from GEO

Roth-Zlotnik-2006 0.61%

Gene expression analyses reveal molecular relationships among 20 regions of the human CNS. Roth et al (2006). *Neurogenetics*

Co-expression with 669,062 interactions from GEO

Boldrick-Relman-2002 0.60%

Stereotyped and specific gene expression programs in human innate immune responses to bacteria. Boldrick et al (2002). *Proc Natl Acad Sci U S A*

Co-expression with 111,707 interactions from supplementary material

Perou-Botstein-2000 0.59%

Molecular portraits of human breast tumours. Perou et al (2000). *Nature*

Co-expression with 185,068 interactions from supplementary material

Smirnov-Cheung-2009 0.58%

Genetic analysis of radiation-induced changes in human gene expression. Smirnov et al (2009). *Nature*

Co-expression with 461,500 interactions from GEO

Wang-Cheung-2015 0.57%

Genetic variation in insulin-induced kinase signaling. Wang et al (2015). *Mol Syst Biol*

Co-expression with 411,047 interactions from GEO

Chen-Brown-2002 0.51%

Gene expression patterns in human liver cancers. Chen et al (2002). *Mol Biol Cell*

Co-expression with 282,241 interactions from supplementary material

Perou-Botstein-1999 0.50%

Distinctive gene expression patterns in human mammary epithelial cells and breast cancers. Perou et al (1999). *Proc Natl Acad Sci U S A*

Co-expression with 65,069 interactions from supplementary material

Wu-Garvey-2007 0.48%

The effect of insulin on expression of genes and biochemical pathways in human skeletal muscle. Wu et al (2007). *Endocrine*

Co-expression with 267,109 interactions from GEO

Rosenwald-Staudt-2001 0.40%

Relation of gene expression phenotype to immunoglobulin mutation genotype in B cell chronic lymphocytic leukemia. Rosenwald et al (2001). *J Exp Med*

Co-expression with 114,694 interactions from supplementary material

**Predicted**	6.35%
I2D-vonMering-Bork-2002-High-Yeast2Human<br>Comparative assessment of large-scale data sets of protein-protein interactions. von Mering et al (2002). *Nature*<br>Predicted with 1,196 interactions from I2D	0.89%
I2D-vonMering-Bork-2002-Medium-Yeast2Human<br>Comparative assessment of large-scale data sets of protein-protein interactions. von Mering et al (2002). *Nature*<br>Predicted with 3,009 interactions from I2D	0.59%
I2D-BioGRID-Yeast2Human<br>BioGRID: a general repository for interaction datasets. Stark et al (2006). *Nucleic Acids Res*<br>Predicted with 13,434 interactions from I2D	0.50%
I2D-Chen-Pawson-2009-PiwiScreen-Mouse2Human<br>Mouse Piwi interactome identifies binding mechanism of Tdrkh Tudor domain to arginine methylated Miwi. Chen et al (2009). *Proc Natl Acad Sci U S A*<br>Predicted with 31 interactions from I2D	0.46%
I2D-INNATEDB-Mouse2Human<br>InnateDB: facilitating systems-level analyses of the mammalian innate immune response. Lynn et al (2008). *Mol Syst Biol*<br>Predicted with 1,451 interactions from I2D	0.42%
I2D-Tarassov-PCA-Yeast2Human<br>An in vivo map of the yeast protein interactome. Tarassov et al (2008). *Science*<br>Predicted with 440 interactions from I2D	0.40%
I2D-vonMering-Bork-2002-Low-Yeast2Human<br>Comparative assessment of large-scale data sets of protein-protein interactions. von Mering et al (2002). *Nature*<br>Predicted with 16,063 interactions from I2D	0.38%
Wu-Stein-2010<br>A human functional protein interaction network and its application to cancer data analysis. Wu et al (2010). *Genome Biol*<br>Predicted with 87,829 interactions from supplementary material	0.34%
I2D-IntAct-Mouse2Human<br>The IntAct molecular interaction database in 2010. Aranda et al (2010). *Nucleic Acids Res*<br>Predicted with 3,427 interactions from I2D	0.31%
Stuart-Kim-2003<br>A gene-coexpression network for global discovery of conserved genetic modules. Stuart et al (2003). *Science*<br>Predicted with 24,872 interactions from supplementary material	0.27%
I2D-Yu-Vidal-2008-GoldStd-Yeast2Human<br>High-quality binary protein interaction map of the yeast interactome network. Yu et al (2008). *Science*<br>Predicted with 386 interactions from I2D	0.22%
I2D-Krogan-Greenblatt-2006-Core-Yeast2Human<br>Global landscape of protein complexes in the yeast Saccharomyces cerevisiae. Krogan et al (2006). *Nature*<br>Predicted with 1,823 interactions from I2D	0.19%
I2D-BIND-Rat2Human<br>BIND--a data specification for storing and describing biomolecular interactions, molecular complexes and pathways. Bader et al	0.19%

Predicted 6.35%

I2D-BIND-Rat2Human

(2000). *Bioinformatics*

Predicted with 548 interactions from I2D

I2D-BioGRID-Worm2Human 0.18%

BioGRID: a general repository for interaction datasets. Stark et al (2006). *Nucleic Acids Res*

Predicted with 952 interactions from I2D

I2D-MINT-Rat2Human 0.17%

MINT: a Molecular INTeraction database. Zanzoni et al (2002). *FEBS Lett*

Predicted with 572 interactions from I2D

I2D-MINT-Mouse2Human 0.15%

MINT: a Molecular INTeraction database. Zanzoni et al (2002). *FEBS Lett*

Predicted with 971 interactions from I2D

I2D-BIND-Mouse2Human 0.14%

BIND--a data specification for storing and describing biomolecular interactions, molecular complexes and pathways. Bader et al (2000). *Bioinformatics*

Predicted with 1,186 interactions from I2D

I2D-BioGRID-Mouse2Human 0.12%

BioGRID: a general repository for interaction datasets. Stark et al (2006). *Nucleic Acids Res*

Predicted with 286 interactions from I2D

I2D-BIND-Yeast2Human 0.11%

BIND--a data specification for storing and describing biomolecular interactions, molecular complexes and pathways. Bader et al (2000). *Bioinformatics*

Predicted with 1,541 interactions from I2D

I2D-Krogan-Greenblatt-2006-NonCore-Yeast2Human 0.09%

Global landscape of protein complexes in the yeast Saccharomyces cerevisiae. Krogan et al (2006). *Nature*

Predicted with 1,786 interactions from I2D

I2D-IntAct-Fly2Human 0.09%

The IntAct molecular interaction database in 2010. Aranda et al (2010). *Nucleic Acids Res*

Predicted with 3,912 interactions from I2D

I2D-Formstecher-Daviet-2005-Embryo-Fly2Human 0.07%

Protein interaction mapping: a Drosophila case study. Formstecher et al (2005). *Genome Res*

Predicted with 491 interactions from I2D

I2D-MGI-Mouse2Human 0.07%

Ontological visualization of protein-protein interactions. Drabkin et al (2005). *BMC Bioinformatics*

Predicted with 726 interactions from I2D

I2D-IntAct-Rat2Human 0.01%

The IntAct molecular interaction database in 2010. Aranda et al (2010). *Nucleic Acids Res*

Predicted with 1,052 interactions from I2D

I2D-MINT-Worm2Human 0.00%

Predicted 6.35%

I2D-MINT-Worm2Human

MINT: a Molecular INTeraction database. Zanzoni et al (2002). *FEBS Lett*

Predicted with 1,178 interactions from I2D

Co-localization 6.17%

Zhang-Shang-2006 2.58%

The catalytic subunit of the proteasome is engaged in the entire process of estrogen receptor-regulated transcription. Zhang et al (2006). *EMBO J*

Co-localization with 53 interactions from BioGRID

Schadt-Shoemaker-2004 1.65%

A comprehensive transcript index of the human genome generated using microarrays and computational approaches. Schadt et al (2004). *Genome Biol*

Co-localization with 60,126 interactions from GEO

Johnson-Shoemaker-2003 1.11%

Genome-wide survey of human alternative pre-mRNA splicing with exon junction microarrays. Johnson et al (2003). *Science*

Co-localization with 426,332 interactions from GEO

Chen-Huang-2014 0.82%

Using an in situ proximity ligation assay to systematically profile endogenous protein-protein interactions in a pathway network. Chen et al (2014). *J Proteome Res*

Co-localization with 559 interactions from BioGRID

Pathway 4.35%

Wu-Stein-2010 1.54%

A human functional protein interaction network and its application to cancer data analysis. Wu et al (2010). *Genome Biol*

Pathway with 78,010 interactions from supplementary material

REACTOME 1.33%

Pathway with 24,913 interactions from Pathway Commons

NCI_NATURE 0.58%

Pathway with 10,122 interactions from Pathway Commons

CELL_MAP 0.46%

Pathway with 598 interactions from Pathway Commons

IMID 0.41%

Pathway with 1,073 interactions from Pathway Commons

HUMANCYC 0.03%

Pathway with 680 interactions from Pathway Commons

Genetic Interactions 1.40%

Vizeacoumar-Moffat-2013 0.41%

A negative genetic interaction map in isogenic cancer cell lines reveals cancer cell vulnerabilities. Vizeacoumar et al (2013). *Mol Syst Biol*

Genetic Interactions with 201 interactions from BioGRID

BIOGRID-SMALL-SCALE-STUDIES 0.38%

Genetic Interactions 1.40%

BIOGRID-SMALL-SCALE-STUDIES

Genetic Interactions with 489 interactions from BioGRID

Toyoshima-Grandori-2012 0.28%

Functional genomics identifies therapeutic targets for MYC-driven cancer. Toyoshima et al (2012). *Proc Natl Acad Sci U S A*

Genetic Interactions with 101 interactions from BioGRID

IREF-SMALL-SCALE-STUDIES 0.24%

Genetic Interactions with 35 interactions from iRefIndex

Blomen-Brummelkamp-2015 0.08%

Gene essentiality and synthetic lethality in haploid human cells. Blomen et al (2015). *Science*

Genetic Interactions with 127 interactions from BioGRID

Willingham-Muchowski-2003 0.00%

Yeast genes that enhance the toxicity of a mutant huntingtin fragment or alpha-synuclein. Willingham et al (2003). *Science*

Genetic Interactions with 37 interactions from BioGRID

Shared protein domains 0.59%

INTERPRO 0.38%

Shared protein domains with 608,863 interactions from InterPro

PFAM 0.21%

Shared protein domains with 457,054 interactions from Pfam
