# Supplementary material for: Bayesian Inference Associates Rare KDR Variants With Specific Phenotypes in Pulmonary Arterial Hypertension
Source: Circ Genom Precis Med. 2020 Dec 15;14(1):e003155. doi: 10.1161/CIRCGEN.120.003155 (PMC7892262; doi:10.1161/CIRCGEN.120.003155)
Supplement: Supplementary file 2 [file hcg-14-e003155-s002.pdf]

DOI: 10.1161/CIRCGEN.120.003011

Epigenomic Profiles of African American *Transthyretin* Val122Ile Carriers Reveals Putatively Dysregulated Amyloid Mechanisms

Running title: *Pathak et al.; Methylation study of TTR Val122Ile carriers*

Gita A. Pathak, PhD[1,2]; Frank R. Wendt, PhD[1,2]; Antonella De Lillo, PhD[3];

Yaira Z. Nunez, BS[1,2]; Aranyak Goswami, PhD[1,2]; Flavio De Angelis, PhD[1-3];

Maria Fuciarelli, PhD[3]; Henry R. Kranzler, MD[4]; Joel Gelernter, MD[1,2];

Renato Polimanti, PhD[1,2]

[1]Department of Psychiatry, Yale School of Medicine, Yale University, New Haven; [2]Veteran Affairs Connecticut Healthcare System, West Haven, CT; [3]Department of Biology, University of Rome Tor Vergata, Rome, Italy; [4]University of Pennsylvania Perelman School of Medicine & VISN 4 MIRECC, Crescenz VAMC, Philadelphia, PA

Circulation: Genomic and Precision Medicine

Correspondence:

Renato Polimanti, PhD

Veteran Affairs Connecticut Healthcare System

VA CT 116A2, 950 Campbell Avenue

West Haven, CT 06516

Tel: +1 (203) 937-5711 x5745

E-mail: renato.polimanti@yale.edu

Journal Subject Terms: Epigenetics; Cardiomyopathy

This article is published in its accepted form; it has not been copyedited and has not appeared in an issue of the journal. Preparation for inclusion in an issue of *Circulation: Genomic and Precision Medicine* involves copyediting, typesetting, proofreading, and author review, which may lead to differences between this accepted version of the manuscript and the final published version.

Abstract:

Background - The Val122Ile mutation in *Transthyretin* (*TTR*) gene causes a rare, difficult to diagnose hereditary form of cardiac amyloidosis. This mutation is most common in the United States and mainly present in people of African descent. The carriers have an increased risk of congestive heart failure, peripheral edema, and several other non-cardiac phenotypes such as carpal tunnel syndrome, and arthroplasty which are top reasons for ambulatory/outpatient surgeries in the country.

Methods - We conducted first-ever epigenome-wide association study using the Illumina's EPIC array, in Val122Ile carriers of African descent for heart disease (HD) and multiple outpatient surgeries (OS) - an early disease indicator. Differential methylation across genome wide CpG sites was tested between carriers with and without HD and OS. Significant CpG sites were investigated for cis-mQTLs loci, followed by gene ontology and protein-protein interaction (PPI) network. We also investigated the significant CpG sites in a secondary cohort of carriers for replication.

Results - Five differentially methylated sites ($p \leq 2.1e-08$) in genes – *FAM129B*, *SKI*, *WDR27*, *GLS*, and an intergenic site near RP11-550A5.2, and one differentially methylated region containing *KCNA6* and *GALNT3* (p=1.1e-12) were associated with HD. For OS, we observe four sites – two sites in *UBE2E3* and *SEC14L5*, and other two in intergenic regions ($p \leq 1.8e-07$) and three regions overlapping *SH3D21*, *EVA1B*, *LTB4R2* and *CIDEB* ($p \leq 3.9e-07$). Functional protein-interaction module analysis identified *ABCA1* (p=0.001) for HD. Six cis-mQTLs were associated with one of the significant CpG sites (*FAM129B*; p=4.1e-24). We replicated two CpG sites (cg18546846 and cg06641417; $p<0.05$) in an external cohort of biopsy- confirmed cases of TTR amyloidosis. The genes identified are involved in transport and clearance of amyloid deposits (*GLS*, *ABCA1*, *FAM129B*); cardiac fibrosis (*SKI*); and muscle tissue regulation (*SKI*, *FAM129B*).

Conclusions - These findings highlight the link between a complex amyloid circuit and diverse symptoms of Val122Ile.

Key words: amyloid; race and ethnicity; epigenetics; transthyretin; methylation; cardiac amyloidosis

Circulation: Genomic and Precision Medicine

This article is published in its accepted form; it has not been copyedited and has not appeared in an issue of the journal. Preparation for inclusion in an issue of *Circulation: Genomic and Precision Medicine* involves copyediting, typesetting, proofreading, and author review, which may lead to differences between this accepted version of the manuscript and the final published version.

DOI: 10.1161/CIRCGEN.120.003011

Nonstandard Abbreviations and Acronyms

TTR - Transthyretin

OS – Outpatient surgery

HD – Heart Disease

CpG – Cytosine-phosphate Guanine

hATTR - hereditary TTR amyloidosis

SNP – Single Nucleotide Polymorphisms

TSS – Transcription Start Site

Introduction

Circulation: Genomic and Precision Medicine

Hereditary transthyretin amyloidosis, caused by specific disease-causing mutations, is due to a gradual extracellular deposition of amyloid in multiple tissues primarily leading to several clinical signs and symptoms[1]. There are 113 known mutations in the transthyretin (*TTR*) gene[2] giving rise to hereditary form of *TTR* amyloidosis. The tetrameric structure of TTR protein dissociates into dimers and monomers resulting in formation of fibrils. The heterogeneous symptoms of hereditary TTR amyloidosis (hATTR) arises from amyloid deposition in different tissues and organs[2,3]. Val122Ile mutation (NP_000362.1:p.Val142Ile; rs76992529) is the most prevalent *TTR* amyloidogenic variant in the United States and is primarily observed in populations of African descent[4]. This point mutation results in the substitution of an isoleucine with a valine at 122 position of TTR mature protein. According to latest report by International Society of Amyloidosis, the recommended nomenclature is named based on the substitution or deletion of the TTR protein, hence here we use Val122ILe [5]. Extensive amyloid deposition seems to resemble hypertrophic cardiomyopathy such as enlargement or wall thickening leading

This article is published in its accepted form; it has not been copyedited and has not appeared in an issue of the journal. Preparation for inclusion in an issue of *Circulation: Genomic and Precision Medicine* involves copyediting, typesetting, proofreading, and author review, which may lead to differences between this accepted version of the manuscript and the final published version.

to heart failure and atrial fibrillation[6]. These symptoms often are attributed to other population-prevalent cardiovascular risk factors resulting in underestimation of the clinical penetrance of the Val122Ile[7]. The estimated age of onset for non-cardiac precursor phenotypes for hereditary transthyretin amyloidosis is between 30 to 40 years of age[8]. In two retrospective studies, carpal tunnel syndrome preceded hereditary transthyretin amyloidosis diagnosis by 9-10 years[9–11]. According to The Transthyretin Amyloid Outcome Survey (THAOS), several cardiac, gait, gastrointestinal, neurological and renal disorders are prevalent in Val122Ile carriers[4]. Parallel to these findings, another study reported atrial fibrillation, myopathy related to ventricular thickness, gastrointestinal and kidney dysfunction including nausea, vomiting, and neuromuscular dysfunction to be associated with *TTR* mutations[12].

Circulation: Genomic and Precision Medicine

We previously investigated medical history phenotypes in the Yale-Penn cohort associated with Val122Ile carriers of African descent and found heart disease history and having had 10 or more outpatient (ambulatory) surgeries significantly associated with this amyloidogenic variant[13]. One of the top reasons for ambulatory surgery in the United States is arthroplasty[14], which occurs in TTR-carriers years before the expected cardiac dystrophy at advanced ages[15]. These epidemiological findings indicate that atypical phenotypes occurring earlier in life could be connected to the risk of heart failure in Val122Ile carriers. Findings from several studies including ours raise the possibility of non-regulatory molecular factors contributing to the genotype-phenotype correlation[12,16–19]. Therefore, understanding the underlying biological changes in Val122Ile carriers is key to explaining the symptom heterogeneity and earlier onset of atypical phenotypes.

DNA methylation is a heritable non-coding regulatory mechanism causing phenotypic variation[18]. Epigenetic modifications arising from the addition of methyl groups on cytosine-

This article is published in its accepted form; it has not been copyedited and has not appeared in an issue of the journal. Preparation for inclusion in an issue of *Circulation: Genomic and Precision Medicine* involves copyediting, typesetting, proofreading, and author review, which may lead to differences between this accepted version of the manuscript and the final published version.

DOI: 10.1161/CIRCGEN.120.003011

phosphate-guanosine (CpG) sites[20] could contribute to molecular mechanisms involved in *TTR* amyloidosis. So far, no study has investigated this hypothesis. Aberrant methylation profiles have been implicated in increasing accelerating the progression of common and rare diseases[21] . The accumulation of amyloid-fibrils within or around cellular structures of the tissue result in damage invoking an immune response[22]. The inter-individual variation in response to site of damage invokes an acute phase response[22]. DNA methylation profiles have the potential to capture individual-level variability and highlight mechanisms involved in *TTR* amyloidosis[23]. Thus, we conducted the first epigenome-wide association study of *TTR* Val122Ile carriers to investigate the association of methylation changes with medical history of heart disease and outpatient surgeries.

American Heart Association.

Methods

Circulation: Genomic and Precision Medicine

The intensity files of methylation data have been uploaded to Gene Expression Omnibus (GSE154683). The authors declare that all supporting data are available within the article [and its online supplementary files]. For further inquiries, please contact the corresponding author.

Author contributions

Yale-Penn study was approved by the institutional review boards at each participating site including receiving participants consent. The current study was approved under the protocol 2000023750 by the institutional review board (IRB) at Yale University School of Medicine. All intensity files of methylation data have been made publicly available at the GSE154683 and can be accessed at https://www.ncbi.nlm.nih.gov/geo/. The authors declare that all supporting data are available within the article [and its supplementary files]. Detailed methods are available in the Supplementary section.

This article is published in its accepted form; it has not been copyedited and has not appeared in an issue of the journal. Preparation for inclusion in an issue of *Circulation: Genomic and Precision Medicine* involves copyediting, typesetting, proofreading, and author review, which may lead to differences between this accepted version of the manuscript and the final published version.

Results

Differentially methylated sites

We investigated differentially methylated sites with respect to two binary outcomes: – a) self-reported heart disease and b) a history of 10 or more outpatient surgeries. After performing the recommended quality control procedure, we investigated 737,385 sites in 96 individuals. In addition to performing standard association analysis, we permuted the phenotypes (p_{perm}: p-value from permutation), which accounted for the case-control imbalance, yielding nine significant CpG sites (Figure 1). Five sites were hypomethylated in individuals with heart disease: cg06641417 (*FAM129B*; logFC=-1.822; p_{perm}=1.6e-08), cg26033908 (*SKI*; logFC=-1.615; p_{perm}=1.7e-08), cg14890866 (*WDR27;* logFC=-2.028; p_{perm}=3.0e-08), cg15522719 (*GLS;* logFC=-1.731; p_{perm}=4.7e-08) and cg18546846 (RP11-550A5.2; intergenic; logFC=-0.786; p_{perm}=2.2e-08). The CpG sites mapped to *FAM129B* and *SKI* are located in gene bodies, cg14890866 is between the 5'UTR (Un-Translated Region) and TSS200 (− 200 nt upstream of Transcription start site[24]) of *WDR27*, while cg15522719 is in TSS150 at *GLS*. Four methylation sites were associated with 10 or more outpatient surgeries: cg13998023 (*UBE2E3*; logFC=-2.632; p_{perm}=1.8e-07), cg05189127 (intergenic; logFC=1.885; p_{perm}=1.4e-07), cg03718655 (*SEC14L5*; logFC=-2.673; p_{perm}=1.5e-07) and cg25814327 (intergenic; logFC=-2.075; p_{perm}=3e-08). Three sites were hypomethylated, while cg05189127 (intergenic) was hypermethylated. The two sites that were annotated to genes were in the 5'UTR (*UBE2E3*) and TSS200 (*SEC14L5*). Details of the association result and annotation are reported in Supplementary file (Table S1).

Differentially methylated regions

For heart disease, one region on chromosome 12 overlapping *KCNA6* and *GALNT3* (p=1.1e-12) was differentially methylated. Associations with more than 10 outpatient surgeries were

Circulation: Genomic and Precision Medicine

This article is published in its accepted form; it has not been copyedited and has not appeared in an issue of the journal. Preparation for inclusion in an issue of *Circulation: Genomic and Precision Medicine* involves copyediting, typesetting, proofreading, and author review, which may lead to differences between this accepted version of the manuscript and the final published version.

identified on chromosome 1 (*SH3D21; EVA1B;* p=1.3e-09), chromosome 10 (intergenic region; p=1.7e-08) and chromosome 14 (*LTB4R2; CIDEB*; p=3.9e-07). Methylation levels among all sites were positively correlated within each region. (Figure 2; Supplementary file; Table S2)

Overrepresented gene ontology and PPI networks

Differentially methylated sites and regions were annotated to their respective genes using UCSC RefGene: hg19 genome build. The gene ontology (GO) analysis identified 15 significantly enriched pathways in GO's biological process. *GLS, SKI*, *GALNT8,* and *KCNA6* are involved in protein oligomerization ($FDR_{p\text{-}value}$=4.8e-03) and *KCNA6* and *GALNT8*, which are located near one another, are involved in potassium ion transport ($FDR_{p\text{-}value}$=4.9e-02). *FAM12B* and *SKI* ($FDR_{p\text{-}value}$=4.8e-03 to 3.2e-02) participate in the development of various tissue types – myotubules, and skeletal and striated muscles (Figure 3; Supplementary file, Table S3).

We also investigated the methylation sites for differentially methylated functional modules using the R package *FEM* [25]. The CpG sites are weighted based on their location in the genes, which are then mapped to a protein-protein interaction (PPI) network. For each module (i.e. PPI network) identified, the seed gene is the primary gene to which other functionally related genes are connected. For heart disease, we found the *ABCA1* module to be significant (p=0.001) and target genes identified within the module were: *ABCA1*, *SNTB2, BLOC1S2* and *LIN7B* (p<0.05). The *EXOSC4* gene module was associated with the phenotype of 10 or more outpatient surgeries, and it was the only gene that was a target (Figure 4; Supplementary file, Table S4).

Local quantitative trait loci for methylated sites (mQTL)

We tested SNP associations with nine methylation sites that were epigenome-wide significant with the two phenotypes investigated. The cis-mQTL loci were defined as SNPs within ±1 Mb of

American Heart Association.

Circulation: Genomic and Precision Medicine

This article is published in its accepted form; it has not been copyedited and has not appeared in an issue of the journal. Preparation for inclusion in an issue of *Circulation: Genomic and Precision Medicine* involves copyediting, typesetting, proofreading, and author review, which may lead to differences between this accepted version of the manuscript and the final published version.

the significant CpG site. The sites were considered significant based on an $FDR_{p\text{-value}} < 0.05$ and genomic corrected p-value ($p_{gc}<0.05$). We found six SNPs, rs192528579, rs182192023, rs114553373, rs187644239, rs114896522, and rs139996037 significantly associated (Figure 5; p=4.1e-24; Table S5) with site cg06641417. The SNPs are in high linkage disequilibrium (LD>0.8), rs192528579 is in the intronic region of neighboring gene –- *GARNL3*; rs182192023, rs114553373, rs187644239 and rs114896522 map to *LRSAM1*. Rs139996037 is a non-coding transcript variant of the *FAM129B*.

Epigenetic age

The epigenetic age (DNAm) was measured using the biological clock developed by Horvath and colleagues, which uses 353 CpG sites[26] and also with a second clock based on 71 CpG sites from Hannum and colleagues[27]. The 'Horvath' clock is considered to be a pan-tissue epigenetic clock, while the 'Hannum' clock is considered to be accurate for whole-blood tissue[28]. Both clocks estimated that carriers with heart disease are of older epigenetic age than carriers without heart disease ($p_{Horvath}$=0.007 and p_{Hannum}=0.0009) (Supplementary file; Table S6). However, the (delta) ΔDNAm age (difference in chronological and biological age) was not significant between the two groups ($p_{\Delta Horvath}$=0.31 and $p_{\Delta Hannum}$=0.57; Supplementary file; Table S7).

Replication of methylation sites in the Italian cohort

We tested the nine CpG sites identified in Val122Ile carriers in an independent cohort of biopsy-confirmed *TTR* amyloidosis cases and healthy controls. We replicated cg18546846 (intergenic; near to RP11-550A5.2; p= 0.021) and cg06641417 (*FAM129B*; p=0.016) at nominal significance (p<0.05).

Circulation: Genomic and Precision Medicine

This article is published in its accepted form; it has not been copyedited and has not appeared in an issue of the journal. Preparation for inclusion in an issue of *Circulation: Genomic and Precision Medicine* involves copyediting, typesetting, proofreading, and author review, which may lead to differences between this accepted version of the manuscript and the final published version.

DOI: 10.1161/CIRCGEN.120.003011

Discussion

The clinical consequences of the *TTR* Val122Ile mutation remain underappreciated and the syndrome that accompanies this risk mutation, under-diagnosed. Individuals exhibiting early *TTR*-amyloidosis symptoms are more likely to be diagnosed with another condition prior to receiving the diagnosis of *TTR*-amyloidosis[29]. There is nonetheless a greater burden over time towards developing ventricular hypertrophy, reduced left ventricular ejection fraction, and atrial dilation, at a later age[3,6]. We previously showed that African-American carriers of the Val122Ile mutation had a higher prevalence of heart disease and having multiple outpatient surgeries than individuals without the mutation[13]. In the present study, we identified methylation changes associated with these same phenotypes in Val122Ile carriers. We also replicated two CpG sites (RP11-550A5.2; cg18546846 and *FAM129B;* cg06641417) at nominal significance in an external cohort including biopsy confirmed cases of *TTR* amyloidosis[30]. Thus, we hypothesize that the epigenetic changes associated with the pathogenesis heart disease differs from the methylation profile of carriers who are not affected by the disease. Lastly, we used GeneMANIA[31] to interpret the interaction among the significant genes (Supplementary file). We observed that major genes identified in the present study physically interact and share pathways with *TTR* (Figure 6).

ABCA1 (ATP binding cassette transporter A1) identified via the functional network analysis encodes a transporter of cholesterol from apolipoproteins[32]. *ABCA1* regulates Apolipoprotein E (ApoE) levels, with lower expression of *ABCA1* reducing ApoE levels. However, ApoE with ApoA1 (Apolipoprotein A) reduces amyloid deposition twice as fast as inhibition of the expression of *ApoE*. Additionally, amyloid-beta levels were the lowest for the dual-knockout of *APP* (which encodes amyloid precursor protein) and *ABCA1*[33].

American Heart Association.

Circulation: Genomic and Precision Medicine

This article is published in its accepted form; it has not been copyedited and has not appeared in an issue of the journal. Preparation for inclusion in an issue of *Circulation: Genomic and Precision Medicine* involves copyediting, typesetting, proofreading, and author review, which may lead to differences between this accepted version of the manuscript and the final published version.

GLS (glutaminase) is a key contributor to the metabolizing of glutamine to glutamate[34]. Amyloid-beta-treated neurons show elevated glutaminase expression, which increases glutamate levels and disrupts calcium neural regulation[35]. Additionally, neurofibrillary tangles have been shown to coexist with higher glutaminase activity[36]. The hypomethylated site in the transcription start site of the *GLS* gene may indicate its potential involvement in the central nervous system, which supports the recent finding of cerebral amyloid angiopathy in individuals with mutated TTR cardiac amyloidosis[37]. *FAM129B* (aliases; *MEG-3* and *NIBAN2*) is downregulated in tissues with amyloid deposition and animal studies have shown that enhancing the expression of *FAM129B* reduces oxidative damage by reducing amyloid-beta production via PI3K/Akt signaling[38]. Cardiac hypertrophy increases the risk of heart failure. *FAM129B* is overexpressed in heart failure samples, and rodent experiments indicate a potential role of the gene in the apoptosis of cardiac myocytes after myocardial infarction[39]. Rescuing the expression levels of *FAM129B* reverses hypertrophic responses, thus the hypomethylation of the CpG site in *FAM129B* observed in our finding supports the overexpression of the gene in heart failure[40]. The African-American population has a high prevalence of diabetes[41]. *FAM129B* is also overexpressed in cardiomyocytes under high glucose concentration reflecting its role in diabetic cardiomyopathy[42]. Although *SKI* is an inhibitor of TGF-beta-induced fibrosis and is under expressed in cardiac fibrosis, other epigenetic modulators such as miRNAs-34a and 93-c affect both *SKI* and *TGF-beta*, targeting the inhibitory factors of *SKI*, which could rescue cardiac fibrosis[43]. The gene enrichment analysis identified a role for *FAM129B* and *SKI* in the development of myotube cells and skeletal muscle fiber and organ, and striated muscle cell development.

Circulation: Genomic and Precision Medicine

This article is published in its accepted form; it has not been copyedited and has not appeared in an issue of the journal. Preparation for inclusion in an issue of *Circulation: Genomic and Precision Medicine* involves copyediting, typesetting, proofreading, and author review, which may lead to differences between this accepted version of the manuscript and the final published version.

One of the clinical findings associated with cardiac amyloidosis is increased left ventricular wall thickness, which can lead to heart failure[44]. Electrical perturbations resulting from lower potassium repolarizing current leads to a prolonged action potential in heart failure[45]. The role of *KCNA6* and *GALNT8* is associated with potassium ion transport and the transmembrane transport complexes. One of the cardiovascular symptoms of the *TTR* amyloidosis is pronounced diastolic hypertension[7], and diastolic dysfunction is one of the symptoms associated with transthyretin amyloidosis[46] . *WDR27* was reported to be differentially methylated in individuals with significant differences in diastolic blood pressure[26].

Aging is a common denominator to the symptomology of Val122Ile and DNA methylation[47]. Age-related methylation changes measured via "epigenetic clocks" help to identify molecular aging and its disconnect with chronological age. The Horvath clock based on 353 CpG sites and the Hannum clock based on 71 CpG sites have been extensively replicated in various tissues[48]. While these clocks were developed using blood tissues[26,27], Horvath's clock is validated across multiple tissues, while Hannum's clock is more consistent in samples originating from blood tissues. Higher epigenetically derived age has been associated with several cardiovascular disease traits. Hypermethylation of genes that are protective against heart disease, lead to cardiovascular aging and increased risk for coronary disease[49]. The dysregulation of the *ABCA1* gene, the product of which is involved in the transport of cholesterol from the periphery to liver tissue[50] has been associated with different cardiovascular pathologies. The hypermethylation of the *ABCA1* promoter region silences its expression and is associated with coronary artery disease[51]. In contrast, the increased expression of *ABCA1* regulated by ApoA1 leads to reverse cholesterol efflux in hepatic tissue [52]. Elevated high density lipoprotein (HDL) in the liver is a target site for serum amyloid A, an acute phase response protein that is

Circulation: Genomic and Precision Medicine

This article is published in its accepted form; it has not been copyedited and has not appeared in an issue of the journal. Preparation for inclusion in an issue of *Circulation: Genomic and Precision Medicine* involves copyediting, typesetting, proofreading, and author review, which may lead to differences between this accepted version of the manuscript and the final published version.

expressed during amyloidosis[53]. The observed hypomethylation of *ABCA1* and putative increase in gene expression underscores its likely involvement in shifting the methylation milieu and could perhaps explain the cardiac symptomology in a comparatively younger group of Val122Ile carriers. These findings reflect the observational symptomology of carpal tunnel, a common denominator to arthroplasty and hATTR[54].

These findings provide unique insights into epigenomic contrasts related to symptomology in Val122Ile carriers. However, our study has limitations. First, we investigated the Val122Ile polymorphism only for heart disease, though it is possible that we could identify additional differences with individuals who are non-Val122Ile carriers or who present with wild-type transthyretin amyloidosis. Additionally, due to the low frequency of the disease-causing mutation investigated, our study suffers from an imbalance in the ratio of cases to controls. Although the permutation analysis accounting for this imbalance confirmed our results and we replicated two associations in an independent cohort (with mostly different risk variants). Our findings would benefit from replication in a larger, more balanced study to further dissect the underlying disease mechanisms. While the DNAm age was significantly different in the two groups, the ΔDNAm age was not different. It is possible that our study is underpowered to detect delta-DNAm age. Additionally, there may be some biases in applying DNAm age measures developed largely on individuals of European descent to individuals of other ancestries, and such ancestry-stratified DNAm differences have been reported by other studies as well [55–57].

American Heart Association.
Circulation: Genomic and Precision Medicine

Conclusions

Our study is the first to explore the epigenetic changes in *TTR* Val122Ile carriers. Certain Val122Ile carriers in our study presented with heart disease earlier than usually reported by individuals affected by cardiac amyloidosis. This could be due to modifier effects accelerating

This article is published in its accepted form; it has not been copyedited and has not appeared in an issue of the journal. Preparation for inclusion in an issue of *Circulation: Genomic and Precision Medicine* involves copyediting, typesetting, proofreading, and author review, which may lead to differences between this accepted version of the manuscript and the final published version.

the pathogenicity of Val122Ile mutation. Due to the underestimated clinical penetrance of the mutation in the African American population, we leveraged an external secondary dataset with confirmed clinical phenotype as a random population sample. The purpose of this study was to understand possible non-coding mechanisms that may explain the heterogeneous phenotypes observed in Val122Ile carriers with history of heart disease. The epigenetic changes identified affect the regulation of genes involved in the transport of amyloid and regulating striated and smooth muscle, which form key components of amyloidosis and cardiac tissue susceptibility. These findings provide higher resolution on mechanisms underlying the TTR-Val122Ile mutation.

American Heart Association.

Circulation: Genomic and Precision Medicine

Sources of Funding: The study was supported by 'Global ASPIRE TTR Amyloidosis Competitive Grant' from Pfizer Inc. We are grateful to the participants of the Yale-Penn cohort, which was funded under grants RC2 DA028909, R01 DA12690, R01 DA12849, R01 DA18432, R01AA11330, and R01 AA017535. The investigation conducted in the Italian cohort was supported by an Investigator-Initiated Research from Pfizer Inc. to the University of Rome "Tor Vergata". The content reported in the manuscript is solely the responsibility of the authors and does not represent the official views of the NIH or Pfizer. The funding agencies had no role in the study design, data analysis, and results interpretation of the present study.

Disclosures: H.R.K. is a member of the American Society of Clinical Psychopharmacology's Alcohol Clinical Trials Initiative, which over the last three years was sponsored by Alkermes, Ethypharm, Indivior, Lilly, Lundbeck, Otsuka, Pfizer, Arbor Pharmaceuticals, and Amygdala Neurosciences, Inc. H.R.K. and J.G. are named as inventors on PCT patent application #15/878,640 entitled: "Genotype-guided dosing of opioid agonists," filed on 24 January 2018. The other authors report no conflict of interest.

This article is published in its accepted form; it has not been copyedited and has not appeared in an issue of the journal. Preparation for inclusion in an issue of *Circulation: Genomic and Precision Medicine* involves copyediting, typesetting, proofreading, and author review, which may lead to differences between this accepted version of the manuscript and the final published version.

References:

1. Kristen AV, Maurer MS, Rapezzi C, Mundayat R, Suhr OB, Damy T, THAOS investigators. Impact of genotype and phenotype on cardiac biomarkers in patients with transthyretin amyloidosis - Report from the Transthyretin Amyloidosis Outcome Survey (THAOS). *Plos One*. 2017;12:e0173086.

2. Jacobson DR, Alexander AA, Tagoe C, Garvey WT, Williams SM, Tishkoff S, Modiano D, Sirima SB, Kalidi I, Toure A, Buxbaum JN. The prevalence and distribution of the amyloidogenic transthyretin (TTR) V122I allele in Africa. *Mol Genet Genomic Med.* 2016;4:548–556.

3. Buxbaum JN, Ruberg FL. Transthyretin V122I (pV142I)* cardiac amyloidosis: an age-dependent autosomal dominant cardiomyopathy too common to be overlooked as a cause of significant heart disease in elderly African Americans. *Genet Med*. 2017;19:733–742.

4. Maurer MS, Hanna M, Grogan M, Dispenzieri A, Witteles R, Drachman B, Judge DP, Lenihan DJ, Gottlieb SS, Shah SJ, et al. Genotype and phenotype of transthyretin cardiac amyloidosis: THAOS (transthyretin amyloid outcome survey). *J Am Coll Cardiol.* 2016;68:161–172.

5. Benson MD, Buxbaum JN, Eisenberg DS, Merlini G, Saraiva MJM, Sekijima Y, Sipe JD, Westermark P. Amyloid nomenclature 2018: recommendations by the International Society of Amyloidosis (ISA) nomenclature committee. *Amyloid*. 2018;25:215–219.

6. Mankad AK, Shah KB. Transthyretin Cardiac Amyloidosis. *Curr Cardiol Rep.* 2017;19:97.

7. Shah KB, Mankad AK, Castano A, Akinboboye OO, Duncan PB, Fergus IV, Maurer MS. Transthyretin cardiac amyloidosis in black americans. *Circ Heart Fail*. 2016;9:e002558.

8. Jercan A, Ene A, Jurcut R, Draghici M, Badelita S, Dragomir M, Dobrea C, Popescu M, Jardan D, Stoica E, Iacob S, Codita I, Stan C, Coriu D. Clinical characteristics in patients with hereditary amyloidosis with Glu54Gln transthyretin identified in the Romanian population. *Orphanet J Rare Dis*. 2020;15:34.

9. Karam C, Dimitrova D, Christ M, Heitner SB. Carpal tunnel syndrome and associated symptoms as first manifestation of hATTR amyloidosis. *Neurol Clin Pract*. 2019;9:309–313.

10. Milandri A, Farioli A, Gagliardi C, Longhi S, Salvi F, Curti S, Foffi S, Caponetti AG, Lorenzini M, Ferlini A, Rimessi P, Mattioli S, Violante FS, Rapezzi C. Carpal tunnel syndrome in cardiac amyloidosis: implications for early diagnosis and prognostic role across the spectrum of aetiologies. *Eur J Heart Fail.* 2020;22:507-515.

11. Sekijima Y, Uchiyama S, Tojo K, Sano K, Shimizu Y, Imaeda T, Hoshii Y, Kato H, Ikeda S. High prevalence of wild-type transthyretin deposition in patients with idiopathic carpal tunnel

Circulation: Genomic and Precision Medicine

This article is published in its accepted form; it has not been copyedited and has not appeared in an issue of the journal. Preparation for inclusion in an issue of *Circulation: Genomic and Precision Medicine* involves copyediting, typesetting, proofreading, and author review, which may lead to differences between this accepted version of the manuscript and the final published version.

syndrome: a common cause of carpal tunnel syndrome in the elderly. *Hum Pathol*. 2011;42:1785–1791.

12. De Lillo A, De Angelis F, Di Girolamo M, Luigetti M, Frusconi S, Manfellotto D, Fuciarelli M, Polimanti R. Phenome-wide association study of TTR and RBP4 genes in 361,194 individuals reveals novel insights in the genetics of hereditary and wildtype transthyretin amyloidoses. *Hum Genet*. 2019;138:1331–1340.

13. Polimanti R, Nuñez YZ, Gelernter J. Increased Risk of Multiple Outpatient Surgeries in African-American Carriers of Transthyretin Val122Ile Mutation Is Modulated by Non-Coding Variants. *J Clin Med*. 2019;8:269.

14. Steiner CA, Karaca Z, Moore BJ, Imshaug MC, Pickens G. Surgeries in Hospital-Based Ambulatory Surgery and Hospital Inpatient Settings, 2014: Statistical Brief #223. - In: Healthcare Cost and Utilization Project (HCUP) Statistical Briefs [Internet]. Rockville (MD): Agency for Healthcare Research and Quality (US); 2006 Feb.
2017 May [updated 2020 Jul 20].

15. Rubin J, Alvarez J, Teruya S, Castano A, Lehman RA, Weidenbaum M, Geller JA, Helmke S, Maurer MS. Hip and knee arthroplasty are common among patients with transthyretin cardiac amyloidosis, occurring years before cardiac amyloid diagnosis: can we identify affected patients earlier? *Amyloid*. 2017;24:226–230.

16. Iorio A, De Lillo A, De Angelis F, Di Girolamo M, Luigetti M, Sabatelli M, Pradotto L, Mauro A, Mazzeo A, Stancanelli C, et al. Non-coding variants contribute to the clinical heterogeneity of TTR amyloidosis. *Eur J Hum Genet*. 2017;25:1055–1060.

17. Iorio A, De Angelis F, Di Girolamo M, Luigetti M, Pradotto LG, Mazzeo A, Frusconi S, My F, Manfellotto D, Fuciarelli M, Polimanti R. Population diversity of the genetically determined TTR expression in human tissues and its implications in TTR amyloidosis. *BMC Genomics*. 2017;18:254.

18. Polimanti R, Di Girolamo M, Manfellotto D, Fuciarelli M. In silico analysis of TTR gene (coding and non-coding regions, and interactive network) and its implications in transthyretin-related amyloidosis. *Amyloid*. 2014;21:154–162.

19. Polimanti R, Di Girolamo M, Manfellotto D, Fuciarelli M. Functional variation of the transthyretin gene among human populations and its correlation with amyloidosis phenotypes. *Amyloid*. 2013;20:256–262.

20. Pathak GA, Silzer TK, Sun J, Zhou Z, Daniel AA, Johnson L, O'Bryant S, Phillips NR, Barber RC. Genome-Wide Methylation of Mild Cognitive Impairment in Mexican Americans Highlights Genes Involved in Synaptic Transport, Alzheimer's Disease-Precursor Phenotypes, and Metabolic Morbidities. *J Alzheimers Dis*. 2019;72:733–749.

Circulation: Genomic and Precision Medicine

This article is published in its accepted form; it has not been copyedited and has not appeared in an issue of the journal. Preparation for inclusion in an issue of *Circulation: Genomic and Precision Medicine* involves copyediting, typesetting, proofreading, and author review, which may lead to differences between this accepted version of the manuscript and the final published version.

21. Zoghbi HY, Beaudet AL. Epigenetics and human disease. *Cold Spring Harb Perspect Biol.* 2016;8:a019497.

22. Planté-Bordeneuve V, Said G. Familial amyloid polyneuropathy. *Lancet Neurol.* 2011;10:1086–1097.

23. Hachiya T, Furukawa R, Shiwa Y, Ohmomo H, Ono K, Katsuoka F, Nagasaki M, Yasuda J, Fuse N, Kinoshita K, et al. Genome-wide identification of inter-individually variable DNA methylation sites improves the efficacy of epigenetic association studies. *NPJ Genom Med.* 2017;2:11.

24. Bibikova M, Barnes B, Tsan C, Ho V, Klotzle B, Le JM, Delano D, Zhang L, Schroth GP, Gunderson KL, Fan J-B, Shen R. High density DNA methylation array with single CpG site resolution. *Genomics*. 2011;98:288–295.

25. Jiao Y, Widschwendter M, Teschendorff AE. A systems-level integrative framework for genome-wide DNA methylation and gene expression data identifies differential gene expression modules under epigenetic control. *Bioinformatics*. 2014;30:2360–2366.

26. Horvath S. DNA methylation age of human tissues and cell types. *Genome Biol.* 2013;14:R115.

27. Hannum G, Guinney J, Zhao L, Zhang L, Hughes G, Sadda S, Klotzle B, Bibikova M, Fan J-B, Gao Y, et al. Genome-wide methylation profiles reveal quantitative views of human aging rates. *Mol Cell*. 2013;49:359–367.

28. Zhang Q, Vallerga CL, Walker RM, Lin T, Henders AK, Montgomery GW, He J, Fan D, Fowdar J, Kennedy M, et al. Improved precision of epigenetic clock estimates across tissues and its implication for biological ageing. *Genome Med*. 2019;11:54.

29. Bishop E, Brown EE, Fajardo J, Barouch LA, Judge DP, Halushka MK. Seven factors predict a delayed diagnosis of cardiac amyloidosis. *Amyloid*. 2018;25:174–179.

30. De Lillo A, Pathak G, De Angelis F, Di Girolamo M, Luigetti M, Sabatelli M, Perfetto F, Frusconi S, Manfellotto D, Fuciarelli M, Polimanti R. Epigenetic profiling of Italian patients identified methylation sites associated with hereditary Transthyretin amyloidosis. *Clin Epigenetics*. 2020;12:176.

31. Montojo J, Zuberi K, Rodriguez H, Bader GD, Morris Q. GeneMANIA: Fast gene network construction and function prediction for Cytoscape. *F1000Res*. 2014;3:153.

32. Sasaki M, Komatsu T, Ikewaki K. Impact of Hepatic ABCA1 (ATP-Binding Cassette Transporter A1) Deletion on Reverse Cholesterol Transport A New Clue in Solving Complex HDL (High-Density Lipoprotein) Metabolism. *Arterioscler Thromb Vasc Biol.* 2019;39:1699–1701.

This article is published in its accepted form; it has not been copyedited and has not appeared in an issue of the journal. Preparation for inclusion in an issue of *Circulation: Genomic and Precision Medicine* involves copyediting, typesetting, proofreading, and author review, which may lead to differences between this accepted version of the manuscript and the final published version.

33. Koldamova R, Staufenbiel M, Lefterov I. Lack of ABCA1 considerably decreases brain ApoE level and increases amyloid deposition in APP23 mice. *J Biol Chem*. 2005;280:43224–43235.

34. Cooper AJL, Jeitner TM. Central role of glutamate metabolism in the maintenance of nitrogen homeostasis in normal and hyperammonemic brain. *Biomolecules*. 2016;6:16.

35. Revett TJ, Baker GB, Jhamandas J, Kar S. Glutamate system, amyloid ß peptides and tau protein: functional interrelationships and relevance to Alzheimer disease pathology. *J Psychiatry Neurosci*. 2013;38:6–23.

36. Fuchsberger T, Martínez-Bellver S, Giraldo E, Teruel-Martí V, Lloret A, Viña J. Aβ Induces Excitotoxicity Mediated by APC/C-Cdh1 Depletion That Can Be Prevented by Glutaminase Inhibition Promoting Neuronal Survival. *Sci Rep*. 2016;6:31158.

37. Schaich CL, Maurer MS, Nadkarni NK. Amyloidosis of the brain and heart: two sides of the same coin? *JACC Heart Fail*. 2019;7:129–131.

38. Yi J, Chen B, Yao X, Lei Y, Ou F, Huang F. Upregulation of the lncRNA MEG3 improves cognitive impairment, alleviates neuronal damage, and inhibits activation of astrocytes in hippocampus tissues in Alzheimer’s disease through inactivating the PI3K/Akt signaling pathway. *J Cell Biochem*. 2019;120:18053–18065.

39. Wu H, Zhao Z-A, Liu J, Hao K, Yu Y, Han X, Li J, Wang Y, Lei W, Dong N et.al. Long noncoding RNA Meg3 regulates cardiomyocyte apoptosis in myocardial infarction. *Gene Ther*. 2018;25:511–523.

40. Zhang J, Liang Y, Huang X, Guo X, Liu Y, Zhong J, Yuan J. STAT3-induced upregulation of lncRNA MEG3 regulates the growth of cardiac hypertrophy through miR-361-5p/HDAC9 axis. *Sci Rep*. 2019;9:460.

41. Chow EA, Foster H, Gonzalez V, McIver L. The disparate impact of diabetes on racial/ethnic minority populations. *Clin Diabetes*. 2012;30:130–133.

42. Chen Y, Zhang Z, Zhu D, Zhao W, Li F. Long non-coding RNA MEG3 serves as a ceRNA for microRNA-145 to induce apoptosis of AC16 cardiomyocytes under high glucose condition. *Biosci Rep*. 2019;39:BSR20190444.

43. Zhang C, Zhang Y, Zhu H, Hu J, Xie Z. MiR-34a/miR-93 target c-Ski to modulate the proliferaton of rat cardiac fibroblasts and extracellular matrix deposition in vivo and in vitro. *Cell Signal*. 2018;46:145–153.

44. Suresh R, Grogan M, Maleszewski JJ, Pellikka PA, Hanna M, Dispenzieri A, Pereira NL. Advanced cardiac amyloidosis associated with normal interventricular septal thickness: an uncommon presentation of infiltrative cardiomyopathy. *J Am Soc Echocardiogr*. 2014;27:440–447.

Circulation: Genomic and Precision Medicine

This article is published in its accepted form; it has not been copyedited and has not appeared in an issue of the journal. Preparation for inclusion in an issue of *Circulation: Genomic and Precision Medicine* involves copyediting, typesetting, proofreading, and author review, which may lead to differences between this accepted version of the manuscript and the final published version.

DOI: 10.1161/CIRCGEN.120.003011

45. Yang K-C, Nerbonne JM. Mechanisms contributing to myocardial potassium channel diversity, regulation and remodeling. *Trends Cardiovasc Med*. 2016;26:209–218.

46. González-Duarte A, Barroso F, Mundayat R, Shapiro B. Blood pressure and orthostatic hypotension as measures of autonomic dysfunction in patients from the transthyretin amyloidosis outcomes survey (THAOS). *Auton Neurosci*. 2019;222:102590.

47. Maurer MS, Mundayat R, Rapezzi C. Reply: Val122Ile mt-ATTR Has a Worse Survival Than wt-ATTR Cardiac Amyloidosis. *J Am Coll Cardiol*. 2017;69:758–759.

48. Unnikrishnan A, Freeman WM, Jackson J, Wren JD, Porter H, Richardson A. The role of DNA methylation in epigenetics of aging. *Pharmacol Ther*. 2019;195:172–185.

49. Zhang W, Song M, Qu J, Liu G-H. Epigenetic modifications in cardiovascular aging and diseases. *Circ Res*. 2018;123:773–786.

50. Ghaznavi H, Mahmoodi K, Soltanpour MS. A preliminary study of the association between the ABCA1 gene promoter DNA methylation and coronary artery disease risk. *Mol Biol Res Commun*. 2018;7:59–65.

51. Guay S-P, Légaré C, Houde A-A, Mathieu P, Bossé Y, Bouchard L. Acetylsalicylic acid, aging and coronary artery disease are associated with ABCA1 DNA methylation in men. *Clin Epigenetics*. 2014;6:14.

52. Vaisman BL, Lambert G, Amar M, Joyce C, Ito T, Shamburek RD, Cain WJ, Fruchart-Najib J, Neufeld ED, Remaley AT, et al. ABCA1 overexpression leads to hyperalphalipoproteinemia and increased biliary cholesterol excretion in transgenic mice. *J Clin Invest*. 2001;108:303–309.

53. Yang M, Liu Y, Dai J, Li L, Ding X, Xu Z, Mori M, Miyahara H, Sawashita J, Higuchi K. Apolipoprotein A-II induces acute-phase response associated AA amyloidosis in mice through conformational changes of plasma lipoprotein structure. *Sci Rep*. 2018;8:5620.

54. Aus dem Siepen F, Hein S, Prestel S, Baumgärtner C, Schönland S, Hegenbart U, Röcken C, Katus HA, Kristen AV. Carpal tunnel syndrome and spinal canal stenosis: harbingers of transthyretin amyloid cardiomyopathy? *Clin Res Cardiol*. 2019;108:1324–1330.

55. Tajuddin SM, Hernandez DG, Chen BH, Noren Hooten N, Mode NA, Nalls MA, Singleton AB, Ejiogu N, Chitrala KN, Zonderman AB, et al. Novel age-associated DNA methylation changes and epigenetic age acceleration in middle-aged African Americans and whites. *Clin Epigenetics*. 2019;11:119.

56. Li C, Wang Z, Hardy T, Huang Y, Hui Q, Crusto CA, Wright ML, Taylor JY, Sun YV. Association of Obesity with DNA Methylation Age Acceleration in African American Mothers from the InterGEN Study. *Int J Mol Sci*. 2019;20:4273.

American Heart Association.

Circulation: Genomic and Precision Medicine

This article is published in its accepted form; it has not been copyedited and has not appeared in an issue of the journal. Preparation for inclusion in an issue of *Circulation: Genomic and Precision Medicine* involves copyediting, typesetting, proofreading, and author review, which may lead to differences between this accepted version of the manuscript and the final published version.

57. Horvath S, Gurven M, Levine ME, Trumble BC, Kaplan H, Allayee H, Ritz BR, Chen B, Lu AT, Rickabaugh TM, et al. An epigenetic clock analysis of race/ethnicity, sex, and coronary heart disease. *Genome Biol*. 2016;17:171.

Figure Legends:

Figure 1: Differentially methylated sites in African American TTR-Val122Ile carriers. (A) Methylation sites that were significantly associated with medical history of heart disease. (B) Methylation sites that were significantly associated for having had 10 or more outpatient surgeries. Each CpG site is represented as a data point, with the x-axis being the genomic location, grouped by chromosome and wherein colors represent alternating chromosomes. The y-axis is the -log10 of the p-value of the CpG site. Significant sites are shown as triangles and labelled with CpG probe name and genic annotation in parentheses, triangles pointing upwards signify hypermethylation, whereas triangles pointing downwards signify hypomethylation.

Figure 2: Differentially methylated regions in TTR-Val122Ile carriers. (A) Regional association with heart disease (B-D) Regional association with 10 or more outpatient surgeries. Each panel displays the association of sites within each region, followed by genomic location, ENSEMBL gene name, DNAse, regulation, SNP tracks (from UCSC browser) and correlation of CpG sites shown as a heatmap.

American Heart Association.

Circulation: Genomic and Precision Medicine

This article is published in its accepted form; it has not been copyedited and has not appeared in an issue of the journal. Preparation for inclusion in an issue of *Circulation: Genomic and Precision Medicine* involves copyediting, typesetting, proofreading, and author review, which may lead to differences between this accepted version of the manuscript and the final published version.

Figure 3: Enriched gene ontology (GO) biological processes. The dendrogram shows the FDRpvalue of the pathway associations and are grouped by similarity of function. The genes involved in each of the processes are highlighted in orange bars.

Figure 4: Functional Protein-Protein Interaction (PPI) Networks. The differentially methylated modules consist of a network of genes based on their functional connectivity using protein-protein interaction. Each module has primary gene which is connected to other target genes in the network. Each module was significant $p<0.05$ using the FEM method (see methods). The genes in blue show hypermethylation and yellow represents hypomethylation. A) ABCA1 module was associated with heart disease and the significant target genes in addition to ABCA1 were SNTB2, BLOC1S2 and LIN7B. B) EXOSC4 module was associated with outpatient surgeries and also was the only significant gene in the network.

American Heart Association.
Circulation: Genomic and Precision Medicine

Figure 5: Local-mQTL associated with site – cg06641417 mapped to the FAM129B gene. The top panel displays single nucleotide polymorphisms (SNPs) associated with CpG site – cg06641417 as a data point and color coded based on linkage disequilibrium with the top lead SNP in purple. The x-axis shows gene annotation (hg19) of the region and the y-axis displays the -log10 of p-value. The following panels present various annotations of the reported SNPs i.e. CADD - Combined Annotation Dependent Depletion, and RegulomeDB – score to identify regulatory variants. The bottom panel highlights the chromatin states of various regulatory features being putatively affected from chromatin markers observed in aorta tissue cell line. Visualization made in FUMA.

This article is published in its accepted form; it has not been copyedited and has not appeared in an issue of the journal. Preparation for inclusion in an issue of *Circulation: Genomic and Precision Medicine* involves copyediting, typesetting, proofreading, and author review, which may lead to differences between this accepted version of the manuscript and the final published version.

DOI: 10.1161/CIRCGEN.120.003011

Figure 6: Gene network of significant genic hits and their relationship with TTR. The genes circled in red are the query genes that were identified from the reported analysis. The network shows intermediate genes that connect the query genes based on different interactions as shown in the legend. The lines are colored based on the type of network domain shown in the figure.

American Heart Association.

Circulation: Genomic and Precision Medicine

This article is published in its accepted form; it has not been copyedited and has not appeared in an issue of the journal. Preparation for inclusion in an issue of *Circulation: Genomic and Precision Medicine* involves copyediting, typesetting, proofreading, and author review, which may lead to differences between this accepted version of the manuscript and the final published version.

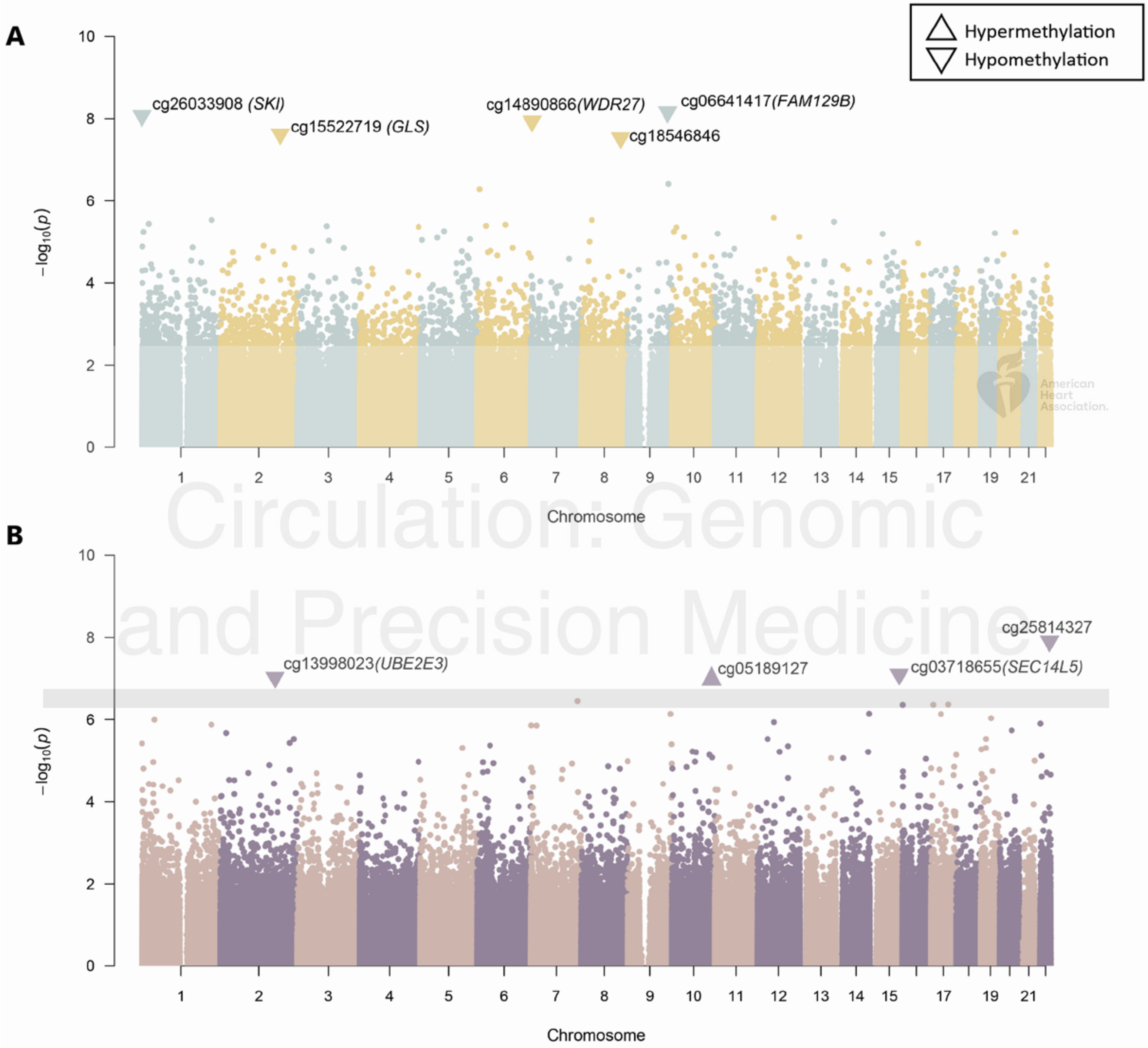

Circulation: Genomic and Precision Medicine
American Heart Association.

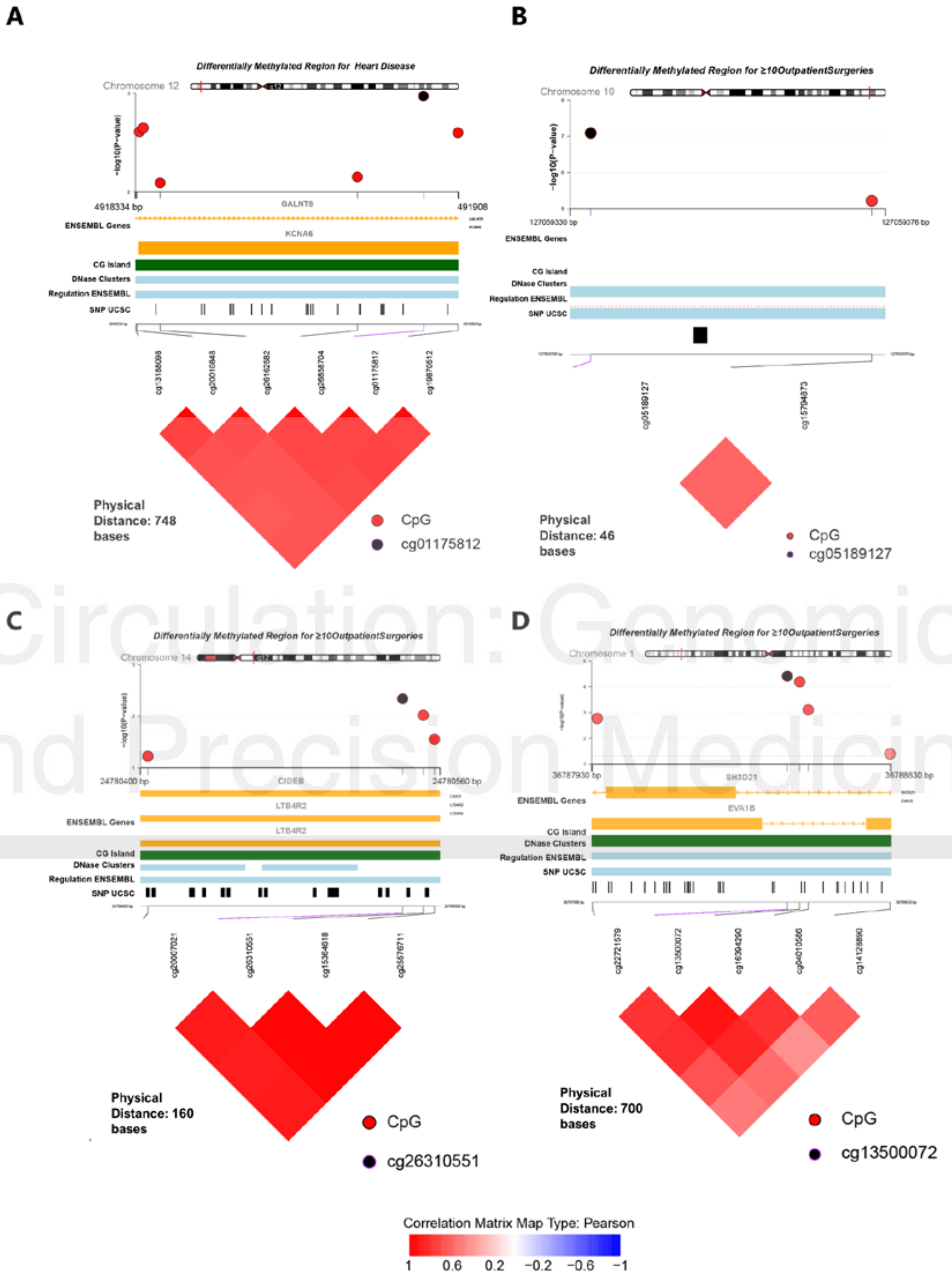

American Heart Association.
Circulation: Genomic and Precision Medicine

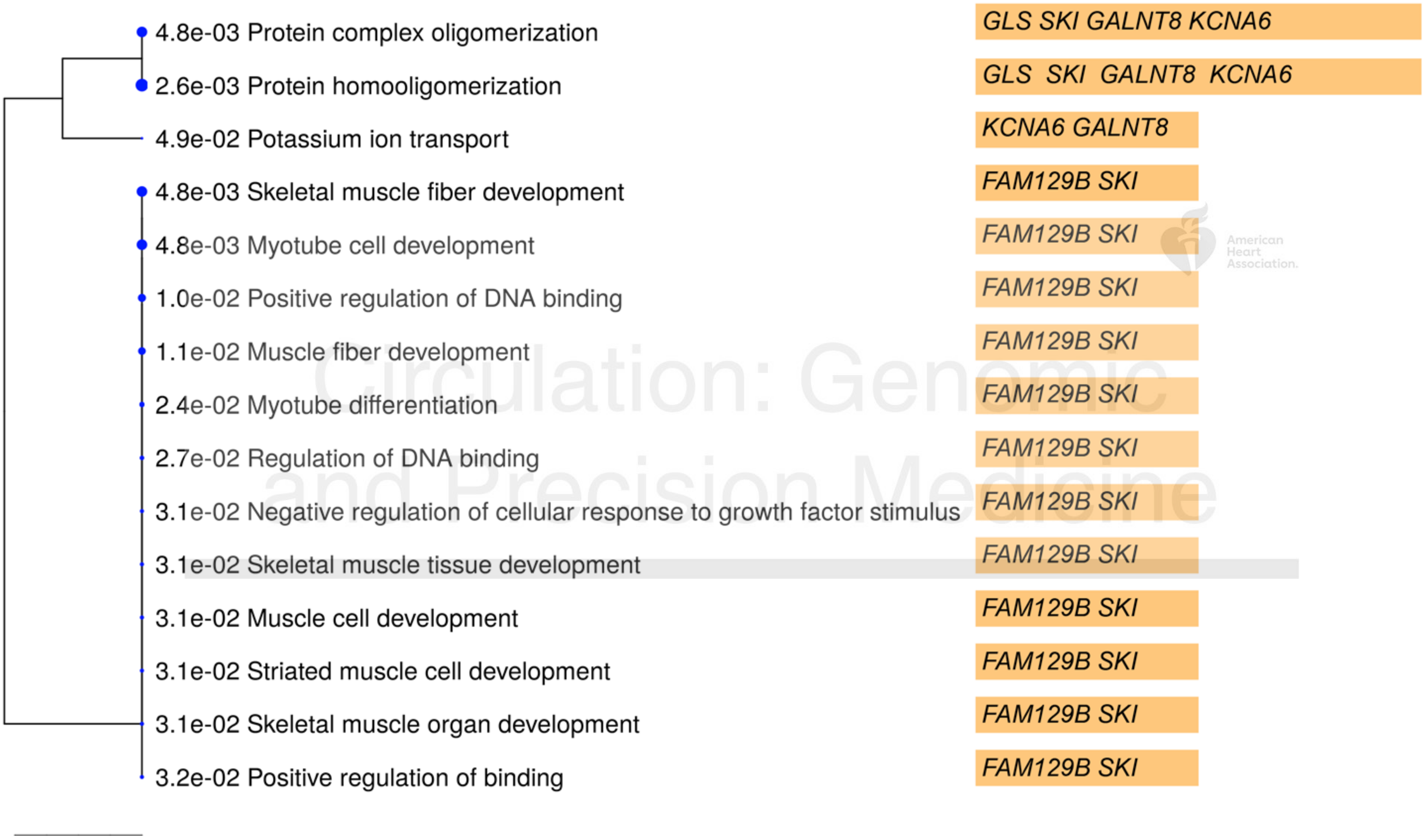

American Heart Association.
Circulation: Genomic and Precision Medicine

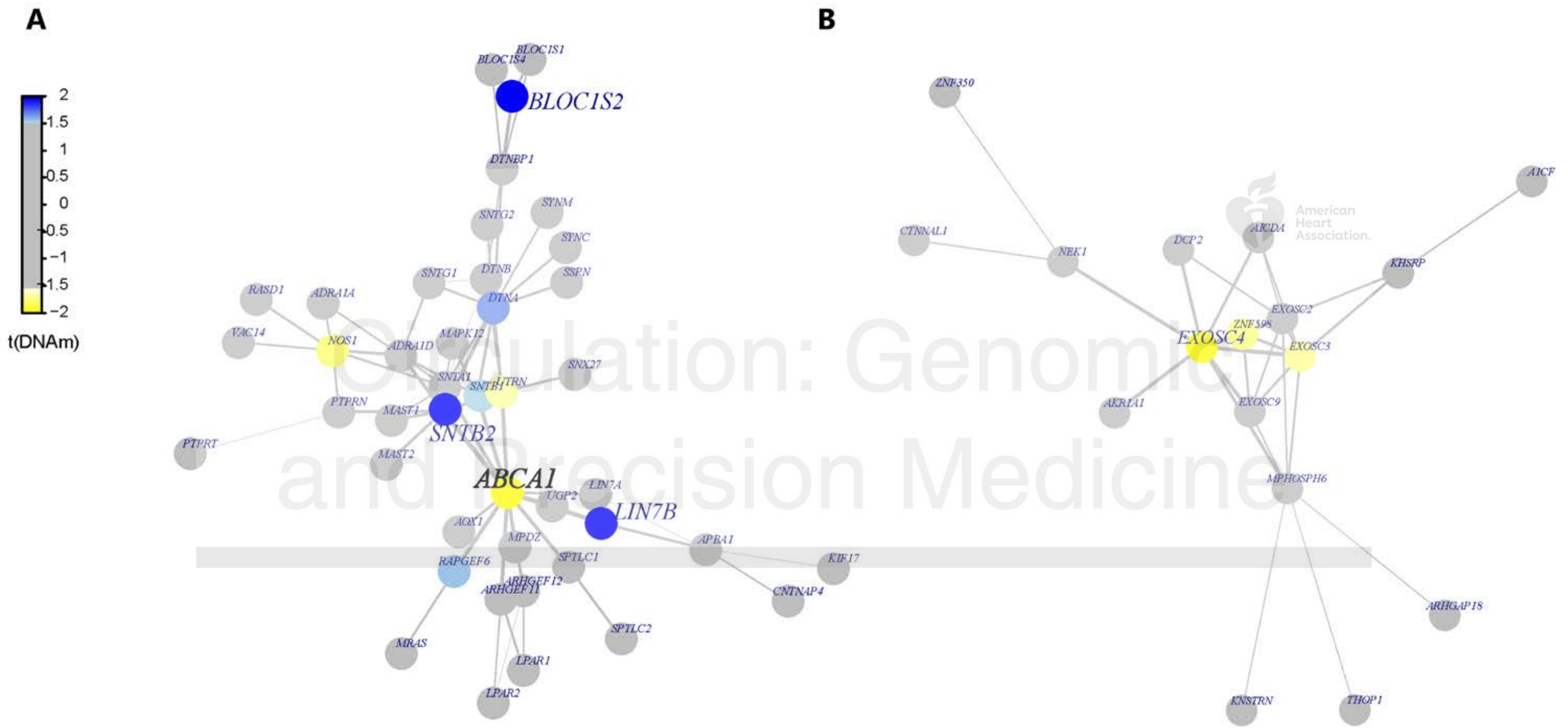

American Heart Association.
Circulation: Genomic and Precision Medicine

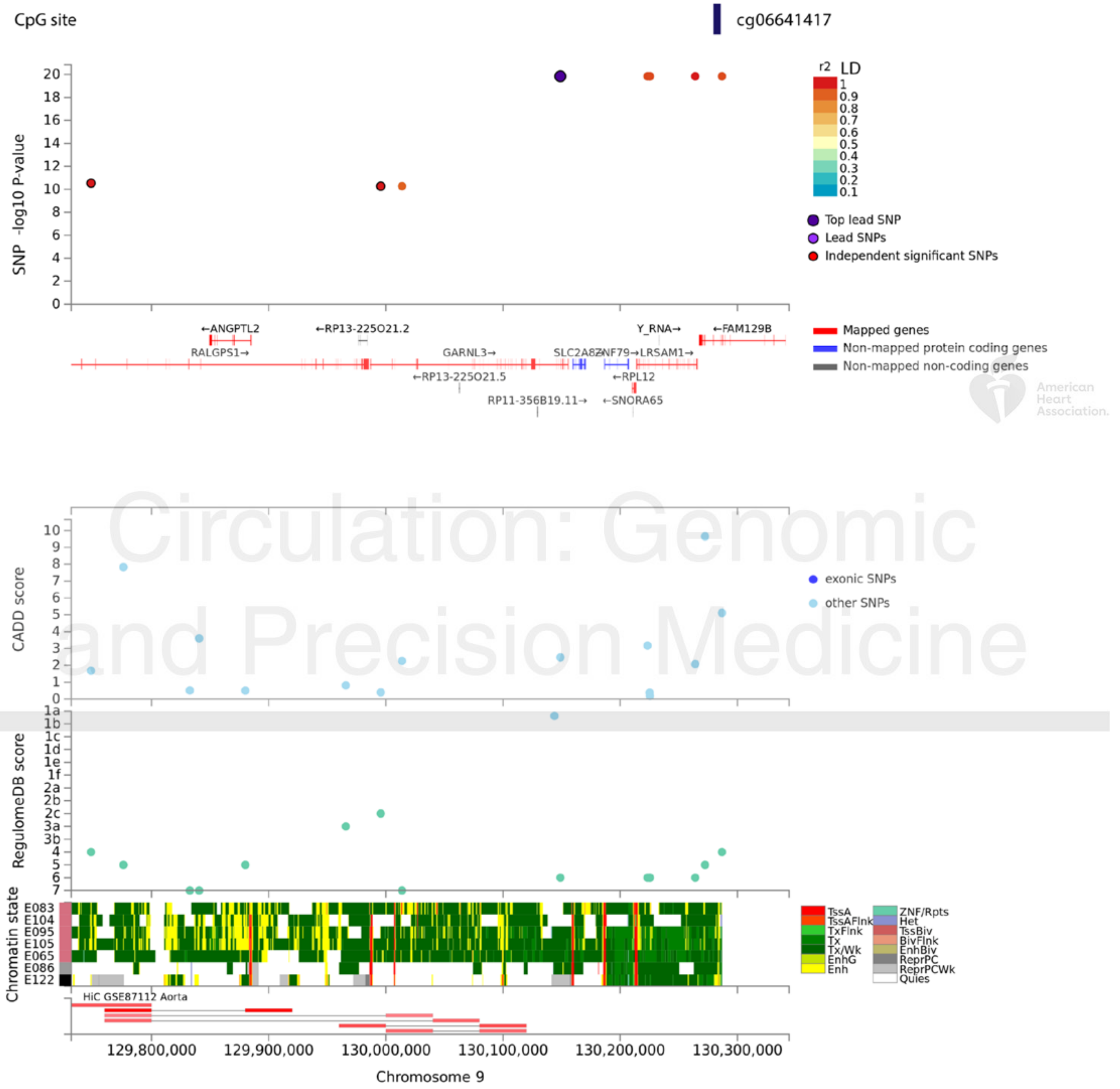

Circulation: Genomic and Precision Medicine

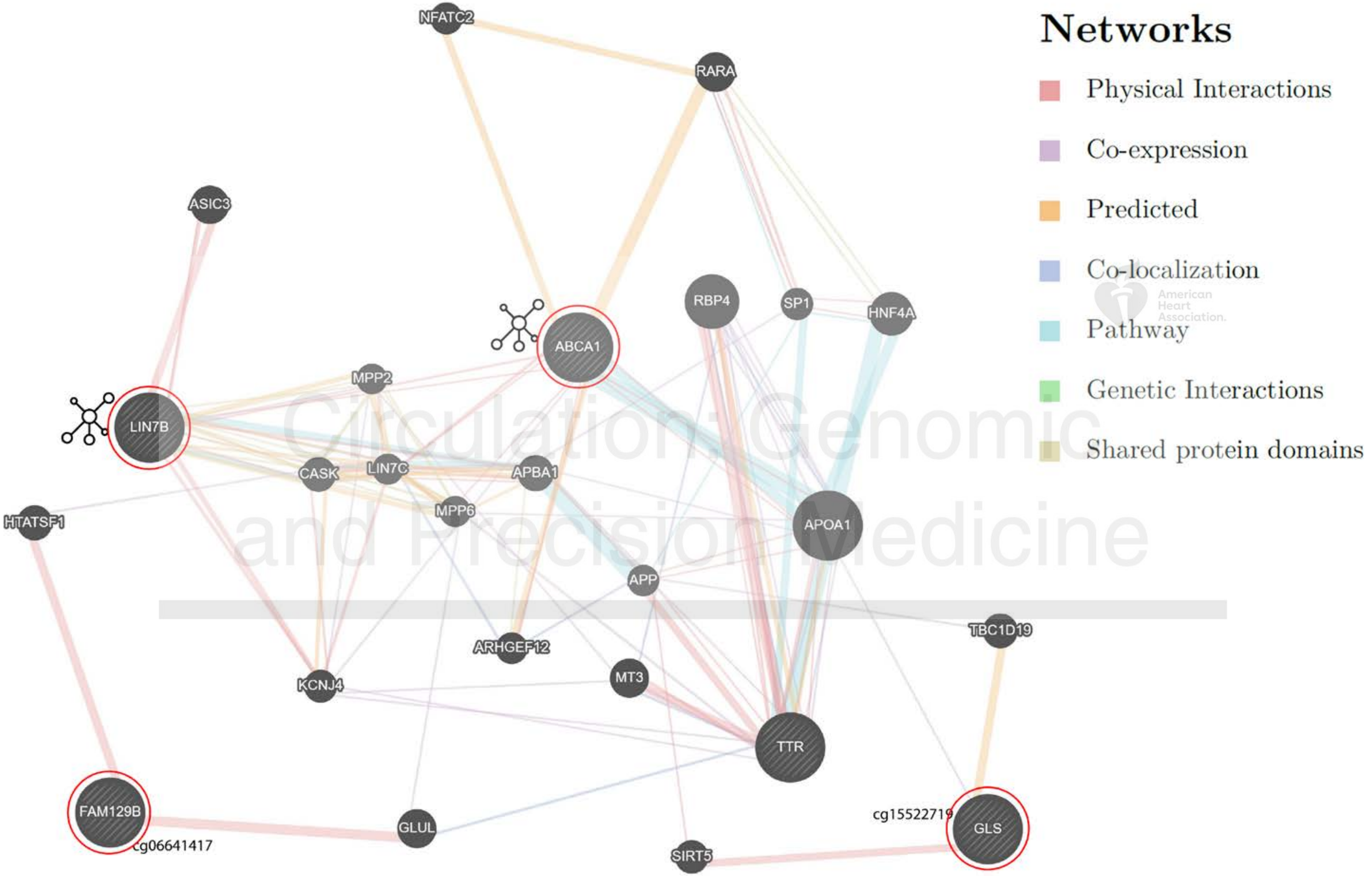

Circulation: Genomic and Precision Medicine
American Heart Association.
